# Supplementary material for: Clinical experiences, current approaches, opinions and awareness of healthcare professionals regarding the audio-vestibular consequences of individuals with traumatic brain injury: a cross-sectional online survey study
Source: BMJ Open. 2024 Jul 8;14(7):e078017. doi: 10.1136/bmjopen-2023-078017 (PMC11256030; doi:10.1136/bmjopen-2023-078017)
Supplement: Supplementary data [file bmjopen-2023-078017supp001.pdf]

Supplemental Appendix 1. English and Turkish version of the survey

English Version of Survey

University of Nottingham
UK | CHINA | MALAYSIA

Healthcare professionals and Audio- vestibular consequences of Traumatic Brain Injury

Page 1: Information and Consent Page for an OnlineSurvey/Questionnaire

Information and Consent page for an Online Survey/Questionnaire

Study Title: Experiences and opinions of healthcare professionals about the audio-vestibular findings of individuals with traumatic brain injury

Research Team:

Name of supervisors:

Kathryn Fackrell, Senior Research Fellow

Laura Edwards, Clinical Associate Professor in the Faculty of Medicine and Health Sciences

Co-investigator:

Kubra Bolukbas, PhD in Hearing Sciences

Faculty of Medicine and Health Sciences Research Ethics Ref: FMHS 462-0222

This study investigates the opinions, experiences and approaches of healthcare professionals ***(excluding ENT/audiology specialists)*** who deal with adults with traumatic brain injury, with a focus on the auditory and balance status of these patients.

Thank you for your interest. You are invited to take part because you are a healthcare professional ***(excluding ENT/audiology specialists)*** who works with adult traumatic brain injury patients. Please read through the following information before agreeing to participate. You can ask any questions before deciding by contacting the researchers (details below). Taking part is entirely voluntary.

What will I be asked to do?

After clicking the 'next' button at the end of this information page you will be asked to provide informed consent to participate in this study. You will then be asked to provide some basic demographic information related to your profession (i.e. profession, years of occupation, type of institution) and will also answer some questions about your experiences, opinions, and approaches in relation to audio-vestibular findings of traumatic brain injury patients. It should take you a maximum of 20 minutes to complete. We would like you to answer all questions as honestly and completely as possible. You can withdraw at any point during the questionnaire for any reason, before submitting your answers by clicking the Exit button/closing the browser. The data will only be uploaded on completion of the questionnaire by clicking the FINISH button on the final page. At this point, it will not be possible to withdraw your answers.

What are the disadvantages of taking part?

We are asking you to give up your valuable time to take part and this is appreciated.

What are the advantages of taking part?

Your contribution, along with others, will contribute to researchers' understanding of the awareness of healthcare professionals about the auditory and balance status of patients with traumatic brain injury. It will also contribute to the understanding of the international determination of the approaches to audio-vestibular problems in these patients. Future research may also be conducted based on the results of this study.

Who will know I have taken part in the study?

No one will know you have taken part in this study because we will not ask for your name or any other personal identification details (ID) during this survey. Your IP address will not be visible to or stored by the research team because an online survey platform is being used which receives and stores an IP address but enables this detail to be filtered out before it is transferred to the research team. As with any online related activity, the risk of breach is possible but this risk is being minimised by using a platform on an encrypted webpage.

What will happen to your data?

When you have clicked the submit button at the end of the questionnaire, it will be uploaded into a password-protected database with a code number. The research team will not be able to see who it is from and for this reason, it will not be possible to withdraw the data at this point. Your data (research data) will be stored in a password-protected folder on a restricted-access server at the university under the terms of its data protection policy. Data is kept for a minimum of 7 years and then destroyed.

This survey is for a PhD project and the answers received from all participants will be combined in a password-protected database ready for analysis. The results will be written up as a dissertation and may be used in academic publications and presentations. The overall anonymised data from this study may be shared for use in future research and teaching (with research ethics approval).

The only personal data we will receive is your e-mail if you contact us to ask further questions or need support. This will be received and handled separately from your completed questionnaire and it will not be possible to link the sets of data. Your e-mail address will only be kept as long as needed to resolve your problem. It will then be destroyed. For further information about how the university processes personal data please see: https://www.nottingham.ac.uk/utilities/privacy.aspx/

Who will have access to your data?

The University of Nottingham is the data controller (legally responsible for data security) and the Supervisor of this study (named above) is the data custodian (manages access to the data) and as such will determine how your data is used in the study. Your research and personal data will be used for the purposes of the research only. Research is a task that we perform in the public interest.

Responsible members of the University of Nottingham may be given access to data for monitoring and/or audit of the study to ensure it is being carried out correctly.

If you have any questions or concerns about this project, please contact:

Kubra Bolukbas, E-mail: kubra.bolukbas@nottingham.ac.uk or

if you have any concerns about any aspect of this study please contact the Research Supervisor:

Kathryn Fackrell, Email: kathryn.fackrell@nottingham.ac.uk;

Laura Edwards, Email: laura.edwards@nottingham.ac.uk

BMJ Publishing Group Limited (BMJ) disclaims all liability and responsibility arising from any reliance placed on this supplemental material which has been supplied by the author(s)

If you remain unhappy and wish to complain formally, you should then contact the FMHS Research Ethics Committee Administrator E-mail:

FMHS-ResearchEthics@nottingham.ac.uk

Page 2: Consent Form

Please confirm each box to continue. If you do not accept the consent form, please close the browser. ✱ *Required*

Please select at least 5 answer(s).

- [ ] I confirm that I have read and understood the information on the previous page
- [ ] I am a healthcare professionals dealing with adult traumatic brain injury patients (other than audiologist and/or ENT specialist)
- [ ] I understand that my participation is voluntary and I can end the study at any time and withdraw my data by clicking the EXIT button
- [ ] I understand that my answers are anonymous
- [ ] I understand the overall anonymized data from this study may be used in the future for research (with research ethics approval) and teaching purposes

Page 3: Experiences and opinions of healthcare professionals about the audio-vestibular findings of individuals with traumatic brain injury

1. What is your profession? ✱ *Required*

- ◯ Neurologist
- ◯ Neuropsychiatrist
- ◯ Neurosurgeon
- ◯ General Surgeon
- ◯ Physiatrist or Rehabilitation Medicine Doctor
- ◯ Geriatrician
- ◯ Orthopaedic Surgeon
- ◯ Rehabilitation Nurse
- ◯ Neuropsychologist
- ◯ Speech and Language Therapist
- ◯ Occupational Therapist
- ◯ Physiotherapist
- ◯ Psychologist
- ◯ Other

1.a. If you selected Other, please specify:

2. How many years have you been in your occupation? ✱ *Required*

- ◯ Less than 5 years
- ◯ 5-10 years
- ◯ 10-20 years

- More than 20 years

3. How many years have you been working with adult patients with traumatic brain injury? ✱ *Required*

- Less than 5 years
- 5-10 years
- 10-20 years
- More than 20 years

4. What country do you work in? ✱ *Required*

- Australia
- Azerbaijan
- Belgium
- Canada
- China
- Cyprus
- Denmark
- France
- Germany
- Greece
- India
- Ireland
- Israel
- Italy
- Japan
- Netherlands
- Poland

- Saudi Arabia
- Spain
- Sweden
- Switzerland
- Thailand
- Türkiye
- United Kingdom
- United States of America
- Other

4.a. If you selected Other, please specify:

5. Is your place of work private or state-owned? * Required

- Private
- State-owned

6. How many hours per week do you spend working with traumatic brain injury adult patients? * Required

- Less than an hour
- 1-5 hours
- 5-10 hours
- 10-20 hours
- More than 20 hours

7. The following symptoms may be reported by people who have had a traumatic brain injury. Please indicate how frequently your adult patients, who experienced trauma more than 6 months ago, describe these to you ✱ Required

Please don't select more than 1 answer(s) per row.

Please select at least 12 answer(s).

	All of my patients describe this	Most of my patients describe this	Some of my patients describe this	A few of my patients describe this	None of my patients describe this	Do not know
Hearing loss	☐	☐	☐	☐	☐	☐
Inability to understand speech in quiet	☐	☐	☐	☐	☐	☐
Inability to understand speech in noise	☐	☐	☐	☐	☐	☐
Inability to identify where the sound is coming from	☐	☐	☐	☐	☐	☐
Inability to capture conversations	☐	☐	☐	☐	☐	☐
Tinnitus (ringing or buzzing in the ears)	☐	☐	☐	☐	☐	☐
Hyperacusis (an unusual tolerance to ordinary environmental sounds)	☐	☐	☐	☐	☐	☐
Dizziness	☐	☐	☐	☐	☐	☐
Vertigo	☐	☐	☐	☐	☐	☐

Inability to walk in the dark without a physical problem	☐	☐	☐	☐	☐	☐
Dizziness on different surfaces	☐	☐	☐	☐	☐	☐
Feeling dizzy when asleep or awake due to movement	☐	☐	☐	☐	☐	☐

8. The following symptoms may be reported and/or observed by people who have had a traumatic brain injury. Please indicate how frequently, adults who experienced trauma more than 6 months ago, describe these to you, ✱Required

Please don't select more than 1 answer(s) per row.

Please select at least 10 answer(s).

	All of my patients	More than half of my patients	Some of my patients	A few of my patients	None of my patients	Do not know
Asks you to repeat your speech frequently during your interviews	☐	☐	☐	☐	☐	☐
Reading your lips while you speak	☐	☐	☐	☐	☐	☐
Does not understand you when you are not facing her/him	☐	☐	☐	☐	☐	☐
Difficulty understanding you on the phone	☐	☐	☐	☐	☐	☐

Relatives report complaints that the volume is too high while he/she is watching TV	☐	☐	☐	☐	☐	☐
Avoids coming to appointments because they are concerned they will not understand what you are talking about	☐	☐	☐	☐	☐	☐
Is disturbed by environmental/external sounds during their appointment with you	☐	☐	☐	☐	☐	☐
Reports an inability to tolerate certain sounds	☐	☐	☐	☐	☐	☐
Although their physical and mental problems are partially healed, audio-vestibular impairments affect their activities, such as doing sports, dancing and housework	☐	☐	☐	☐	☐	☐
Being afraid to leave the house without an accompanying person because of their balance disorders	☐	☐	☐	☐	☐	☐

9. Do you ask your adult patients with traumatic brain injury (which occurred more than 6 months ago) about their balance status (dizziness, vertigo etc.) at your appointments? ✱ *Required*

- Yes, I ask everybody
- If their balance status prevents me from performing our appointments and/or my job, I ask
- I prefer not to ask because it is not my expertise
- I do not ask anything if there are no complaints about it
- Do not know

10. If you notice dizziness or balance disorders in your adult patients with traumatic brain injury (which occurred more than 6 months ago), which of the screening methods would you use? You can choose multiple options ✱ *Required*

- Past Pointing test
- Finger-to-Nose test
- Unterberger test (Fukuda step test)
- Romberg test
- Gait analysis
- Eye movements
- Head impulse test
- I prefer not to assess this because it is not my expertise
- Do not know
- Other

10.a. If you selected Other, please specify:

11. In which situation would you consider referring your traumatic brain injury patient (where the trauma occurred more than 6 months ago) to the ENT and/or audiology service? You can choose multiple options ✱ *Required*

- [ ] When I think he/she don't understand my speech and/or don’t hear, immediately
- [ ] When I observe dizziness and/or vertigo, immediately
- [ ] If she/he reports tinnitus or hyperacusis, immediately
- [ ] I would wait for our next appointment to be sure, even if I notice his/her condition
- [ ] Unless the patient complains, I do not refer
- [ ] I think it is an issue that can be ignored as there are many impairments they are struggling with
- [ ] Do not know

12. How many of your adult patients who have had a traumatic brain injury (which occurred more than 6 months ago) do you refer to the audiology or ENT department? * Required

- ○ All of my patients
- ○ More than half of my patients
- ○ Some of my patients
- ○ A few of my patients
- ○ None of my patients
- ○ Do not know

13. How do you check the external ear condition of your adult patients with traumatic brain injury (which occurred more than 6 months ago)? * Required

- ○ Referring to the ENT and/or audiology service
- ○ I prefer to evaluate it myself with otoscopy
- ○ I do not do anything if there are no complaints about it
- ○ I am not interested because it is not my area of expertise
- ○ Do not know

14. Do you think that your traumatic brain injury patients (where the trauma occurred more than 6 months ago) should use hearing aids if they have hearing loss?
* Required

- Yes, definitely
- If they would like, yes
- No need
- I think it is an issue that can be ignored as there are many impairments they are struggling with
- Do not know

15. In which situations can you help your adult patients with traumatic brain injury, who use hearing aids, during their appointment with you? You can choose multiple options
* Required

- I can change the battery of hearing aids
- I can notice whether or not it is working
- I can notice any feedback problem
- I can check the hearing aid ear mould and/or dome functionality
- I do not know anything about hearing aids

16. Did your patient group, who frequently report auditory and/or vestibular complaints after trauma, have a specific aetiology of traumatic brain injury? Can you generalize the trauma aetiology of these patients with auditory and/or vestibular complaints?
* Required

- Yes
- No
- Do not know

16.a. If you answered yes to question 16, what was this aetiology?

- Falls
- Vehicle-related collisions
- Violence
- Sport Injuries
- Other Trauma (Please describe)

16.a.i. If you selected Other, please specify:

Turkish Version of Survey

Sağlık Çalışanları ve Travmatik Beyin Hasarının İşitsel-vestibüler Sonuçları

Page 1: Bilgilendirme Formu

Bilgilendirme ve Onam Formu

Final version 1.2: 04.07.2022

Araştırma Başlığı: Travmatik beyin hasarı olan bireylerin odyo-vestibüler bulgularına ilişkinsağlık profesyonellerinin deneyimleri ve görüşleri

Araştırma Ekibi:

Danışmanlar:

Dr. Kathryn Fackrell, İşitme Bilimleri

Dr. Laura Edwards, Tıp ve Sağlık Bilimleri

Araştırmacı:

Uzm Ody. Kübra Bölükbaş, İşitme Bilimleri Doktora Öğrencisi

Tıp ve Sağlık Bilimleri Fakültesü Araştırma Etiği Ref. No: FMHS 462-0222

Bu çalışma, ***KBB ve odyoloji uzmanlarının dışındaki***, travmatik beyin hasarı olan erişkinlerleilgilenen sağlık profesyonellerinin görüş, deneyim ve yaklaşımlarını bu hastaların işitsel ve denge durumlarına odaklanarak incelemektedir.

İlgiliniz için teşekkür ederiz. ***KBB/odyoloji uzmanları dışında*** travmatik beyin hasarı olan yetişkin hastalarla ilgilenen bir sağlık çalışanı olduğunuz için çalışmamıza katılmaya davetlisiniz. Lütfen katılımcı olmadan önce bilgilendirme ve onam formunu dikkatlice okuyunuz.Herhangi bir sorunuz varsa aşağıda yer alan iletişim kanalları

aracılığıyla araştırmacılara ulaşmaktan lütfen çekinmeyin. Araştırmaya katılım gönüllülük esasına dayanmaktadır.

Ne yapmanızı isteyeceğiz?

Bilgilendirme sayfasının sonundaki "Next (İleri)" butonuna tıkladıktan sonra, bu çalışmaya katılabilmeniz için onam formunu kabul etmeniz istenecektir. Daha sonra, mesleğinizle ilgili demografik bilgiler (mesleğiniz, çalışma süreniz, kurum türü gibi) içeren birkaç soru ve travmatikbeyin hasarı olan yetişkin hastalarınızın işitsel-vestibüler bulgularıyla ilgili deneyimleriniz, görüşleriniz ve yaklaşımlarınızı öğrenmek amacıyla hazırlanan soruları cevaplamanız istenecektir. Tüm anket sorularını cevaplamanız en fazla yirmi (20) dakikanızı alacaktır. Tüm soruları mümkün olduğunca dürüst ve eksiksiz olarak cevaplamanızı rica ediyoruz.
Cevaplarınızı göndermeden önce, "Çıkış" butonuna tıklayarak ve/veya tarayıcınızı kaparatarak herhangi bir noktada, herhangi bir nedenle anketten çekilebilirsiniz. Ancak, son
sayfada bulunan "Bitir (Finish)" butonuna tıkladıktan sonra cevaplarınız tarafımıza gönderilecekve cevaplarınızı geri çekmeniz mümkün olmayacaktır.

Katılımcı olmanızın dezavantajları nelerdir?

Katılımcı olmanız için kıymetli zamanınızdan birkaç dakikanızı anketimize ayırmanız gerekecektir.

Katılımcı olmanızın avantajları nelerdir?

Diğer katılımcılar ve sizin katkınız sayesinde araştırmacılar, travmatik beyin hasarı olan bireylerin işitsel ve vestibüler durumları hakkında KBB ve odyoloji uzmanları haricindeki sağlık uzmanlarının görüşleri, yaklaşımları ve farkındalıkları hakkında bilgi edineceklerdir. Ayrıca, bu bireylerdeki işitsel-vestibüler sorunlara yaklaşımın uluslararası düzeyde nasıl olduğunun tespit edilmesine katkı sağlanacaktır. Böylece, çalışmanın sonuçlarından yola çıkılarak konuya ilişkingelecek araştırmaların da önü açılabilir.

Araştırmacılar çalışmaya katıldığınızı bilecekler mi?

İsim, soyisim ve kimlik bilgisi gibi kişisel verileriniz sorulmadığı için hiç kimse araştırmaya katıldığınızı bilmeyecektir. Ayrıca, cevaplarınız araştırma ekibine iletilirken kullanılan platformunfiltreleme sistemi sayesinde IP adresiniz gözükmeyecek ve saklanmayacaktır. Tüm çevrimiçi platformlarda ihlal riski mümkün olduğu gibi bu platformda da mümkündür. Ancak kişisel veri alınmaması ve şifreli bir sistemin kullanılması sayesinde bu risk minimuma düşürülmüştür.

Verilerinize ne olacak?

Anketin sonundaki "Bitir (Finish)" butonuna tıkladığınızda, anket sonuçlarınız şifre korumalı birveri tabanına kod numarası ile yüklenecektir. Araştırma ekibi anketin kim tarafından cevaplandığını göremeyecek ve bu nedenle, bu noktada, verileri geri çekmeniz

mümkün olmayacaktır. Araştırma verileriniz, Nottingham Üniversitesinin veri koruma politikasışartlarına göre sınırlı erişimli bir sunucuda şifre korumalı bir klasörde saklanacaktır. Veriler enaz 7 yıl saklandıktan sonra imha edilecektir.

Bu anket bir doktora projesi için hazırlanmıştır. Tüm katılımcılardan elde edilen yanıtlar analizedilmeye hazır şifre korumalı bir veritabanında birleştirilecektir. Sonuçlar doktora kapsamındabir tez olarak yazılmasının yanısıra akademik yayınlarda ve sunumlarda kullanılabilir. Bu çalışmadan elde edilen anonimleştirilmiş veriler, etik onayı ile gelecekteki araştırma ve öğrenimde kullanılmak üzere paylaşılabilir.

Daha fazla soru sormak için veya desteğe ihtiyaç duyduğunuzda bizimle iletişime geçerseniz, alacağımız tek kişisel veri e-postanız olacaktır. Ancak bu, tamamlamış olduğunuz anketinizdenayrı olarak ele alınacaktır ve verilerinizi birbirine bağlamak mümkün olmayacaktır. E-posta adresiniz yalnızca sorununuzu çözmek için gerektiği kadar saklanacak ve daha sonra imha edilecektir. Üniversitenin kişisel verileri nasıl işlediği hakkında daha fazla bilgi için lütfen şu adresi ziyaret ediniz: https://www.nottingham.ac.uk/utilities/privacy.aspx/

Verilerinize kim erişebilecek?

Nottingham Üniversitesi veri denetleyicisidir (veri güvenliğinden yasal olarak sorumludur) ve buçalışmanın danışmanları (yukarıda adı geçen) veri sorumlusudur (verilere erişimi yönetmektedirler) ve bu nedenle verilerinizin çalışmada nasıl kullanılacağını belirleyeceklerdir. Yanıtlarınız ve verileriniz yalnızca araştırma amacıyla kullanılacaktır. Araştırmamız, kamu yararını gözeterek gerçekleştirdiğimiz bir projedir.

Nottingham Üniversitesi'nin sorumlu üyelerine, çalışmanın doğru bir şekilde yürütüldüğündenemin olmak için izleme ve/veya denetleme için verilere erişim izni verilebilir.

Bu projeyle ilgili herhangi bir sorunuz veya endişeniz varsa, lütfen iletişime geçiniz:

Kübra Bölükbaş, E-mail: kubra.bolukbas@nottingham.ac.uk or

Bu çalışmanın herhangi bir yönü hakkında bir endişeniz varsa, lütfen araştırma danışmanları ileiletişime geçiniz:

Kathryn Fackrell,

Email: kathryn.fackrell@nottingham.ac.uk

Laura Edwards,

Email: laura.edwards@nottingham.ac.uk

Resmi olarak şikayette bulunmak isterseniz, FMHS Araştırma Etik Kurulu Yöneticisi ile iletişimegeçiniz:

FMHS-ResearchEthics@nottingham.ac.uk

Page 2: Onam Formu

Please select at least 5 answer(s).

- ☐ Önceki sayfadaki bilgileri okuduğumu ve anladığımı onaylıyorum
- ☐ Odyolog ve/veya KBB uzmanı dışında, travmatik beyin hasarı olan yetişkinlerle ilgilenen bir sağlık çalışanıyım
- ☐ Katılımımın gönüllü olduğunu ve çalışmayı istediğim zaman sonlandırabileceğimi ve sayfayı kapatarak verilerimi geri çekebileceğimi anlıyorum
- ☐ Yanıtlarımın anonim olarak görüneceğini anlıyorum
- ☐ Bu çalışmadan elde edilen anonimleştirilmiş verilerin gelecekte etik onayı ile araştırma ve öğrenim amaçları için kullanılabileceğini anlıyor ve kabul ediyorum

Devam edebilmek için lütfen her bir kutucuğu onaylayın. Bilgilendirme ve onam formunukabul etmiyorsanız, lütfen tarayıcınızı kapatarak çıkış yapınız. ✱*Required*

Page 3: Travmatik Beyin Hasarı olan Bireylerin Odyo-vestibülerBulgularına İlişkin Sağlık Profesyonellerinin Deneyimleri ve Görüşleri

1. Mesleğiniz nedir? ✱ *Required*

- Nörolog
- Nöropsikiyatrist
- Beyin Cerrahı
- Genel Cerrahi Uzmanı
- Fiziksel Tıp ve Rehabilitasyon Uzmanı
- Geriatri Uzmanı
- Ortopedi ve Travmatoloji Uzmanı
- Hemşire
- Nöropsikolog
- Dil ve Konuşma Terapisti
- Ergoterapist
- Fizyoterapist
- Psikolog
- Diğer Sağlık Profesyoneli (Lütfen açıklayınız)

1.a. Diğer seçeneğini seçtiyseniz, lütfen açıklayın:

2. Kaç yıldır mesleğinizi yapıyorsunuz?✱ *Required*

- 5 yıldan az
- 5 ile 10 yıl arası
- 10 ile 20 yıl arası
- 20 yılı aşkın süredir

3. Travmatik beyin hasarı olan hastalarla kaç yıldır çalışıyorsunuz? ✱ *Required*

- 5 yıldan az
- 5 ile 10 yıl arası
- 10 ile 20 yıl arası
- 20 yılı aşkın süredir

4. Hangi ülkede çalışıyorsunuz? ✱ *Required*

- Almanya
- Amerika
- Avustralya
- Azerbaycan
- Belçika
- Birleşik Krallık
- Çin
- Danimarka
- Hindistan
- Hollanda
- İrlanda
- İspanya
- İsrail
- İsveç
- İsviçre
- İtalya
- Japonya
- Kanada
- Kıbrıs
- Polonya
- Suudi Arabistan
- Türkiye

- Yunanistan
- Diğer

4.a. Diğer seçeneğini seçtiyseniz, lütfen açıklayın:

5. İş yeriniz özel mi yoksa devlete mi ait? ✱ *Required*

- Özel
- Devlet

6. Travmatik beyin hasarı olan yetişkin hastalarla haftada kaç saat çalışıyorsunuz? ✱ *Required*

- 1 saatten az
- 1 ile 5 saat arası
- 5 ile 10 saat arası
- 10 ile 20 saat arası
- 20 saatten fazla

7. Aşağıdaki şikayetler travmatik beyin hasarı geçirmiş kişiler tarafından bildirilebilir. Lütfen 6 aydan daha uzun süre önce kafa travmasına maruz kalmış hastalarınızın bunları size ne sıklıklatarif ettiğini belirtiniz. ✱ *Required*

Please don't select more than 1 answer(s) per row.

Please select at least 12 answer(s).

	Bütün hastaları mbunu bildiriyor	Hastalarımı nçoğu bunu bildiriyor	Bazı hastaları mbunu bildiriyor	Birkaç hastam bunu bildiriyor	Hiçbir hastam bunu bildirmiyor	Bilmiyorum
İşitme kaybı	☐	☐	☐	☐	☐	☐
Sessiz ortamda konuşmayı anlayamama	☐	☐	☐	☐	☐	☐
Gürültülü ortamda konuşmayı anlayamama	☐	☐	☐	☐	☐	☐
Sesin nereden geldiğini tespit edememe	☐	☐	☐	☐	☐	☐
Konuşmaları yakalayamama	☐	☐	☐	☐	☐	☐
Kulak çınlaması (Tinnitus)	☐	☐	☐	☐	☐	☐
Normal çevresel seslere karşı olağan dışı hassasiyet, tahammülsüzlük (Hiperakuzi)	☐	☐	☐	☐	☐	☐
Dengesizlik, Sersemlik (Dizziness)	☐	☐	☐	☐	☐	☐
Baş Dönmesi (Vertigo)	☐	☐	☐	☐	☐	☐
Fiziksel bir problemi olmaksızın karanlıkta yürüyememe	☐	☐	☐	☐	☐	☐

Farklı yüzeylerde yürürken dengesizlik (dizziness)	☐	☐	☐	☐	☐	☐
Harekete bağlı olarak uykuda veya uyanıkken baş dönmesi hissetmek	☐	☐	☐	☐	☐	☐

8. Aşağıdaki belirtiler travmatik beyin hasarı geçirmiş kişiler tarafından bildirilebilir ve/veyagözlemlenebilir. Lütfen 6 aydan daha uzun süre önce kafa travmasına maruz kalmış hastalarınızın bunları size ne sıklıkla bildirdiğini belirtiniz ✱*Required*

Please don't select more than 1 answer(s) per row.

Please select at least 10 answer(s).

	Hastalarımın hepsi	Hastalarımın yarısından fazlası	Bazı hastalarım	Birkaç hastam	Hastalarımın hiçbiri bunu bildirmiyor	Bilmiyorum
Görüşmeleriniz sırasında konuşmanızı sıksık tekrarlamanızı ister	☐	☐	☐	☐	☐	☐
Konuşurken dudaklarınızı okur	☐	☐	☐	☐	☐	☐
Yüzünüz ona dönük olmadığında sizianlamaz	☐	☐	☐	☐	☐	☐
Telefonda sizi anlamakta zorluk çeker	☐	☐	☐	☐	☐	☐

Hasta yakınları hastanın çok yüksek sesle televizyon izlemeye başladığından şikayet eder	☐	☐	☐	☐	☐	☐
Ne hakkında konuştuğunuzu anlayamamaktan endişelendiği için randevularınıza gelmekten kaçınır	☐	☐	☐	☐	☐	☐
Sizinle görüşmesi sırasında çevresel/dış seslerden rahatsız olur	☐	☐	☐	☐	☐	☐
Belirli seslere tahammül edemediğini bildirir	☐	☐	☐	☐	☐	☐
Fiziksel ve zihinsel sorunları kısmen iyileşmiş olsa da, işitsel-vestibüler bozukluklar spor yapma, dans etme ve ev işi yapma gibi aktivitelerini etkiler	☐	☐	☐	☐	☐	☐

Denge bozuklukları nedeniyle refakatçi olmadan evden çıkmaya korkar	☐	☐	☐	☐	☐	☐

9. 6 aydan daha uzun süre önce meydana gelen travmatik beyin hasarına maruz kalmış hastalarınıza randevularınızda denge durumlarının nasıl olduğunu (sersemlik, başdönmesi vb.) soruyor musunuz? ✱ *Required*

- ○ Evet, tüm hastalarıma sorarım
- ○ Denge durumları randevularımızı etkiliyorsa ve/veya işimi yapmamı engelliyorsa sorarım
- ○ Bu konu uzmanlık alanım olmadığı için sormamayı tercih ediyorum
- ○ Bu konuda herhangi bir şikayeti yoksa hiçbir şey sormam
- ○ Bilmiyorum

10. 6 aydan daha uzun süre önce travmatik beyin hasarına maruz kalan hastalarınızda baş dönmesi ve/veya denge bozukluğu fark ederseniz, aşağıdaki tarama yöntemlerinden hangisiniveya hangilerini kullanırsınız? Birden fazla yanıt seçebilirsiniz. ✱ *Required*

- ☐ Past Pointing testi
- ☐ Parmak Burun testi
- ☐ Unterberger testi (Fukuda adım
- ☐ testi)Romberg testi
- ☐ Gait analysis (Yürüme analizi)
- ☐ Göz hareketleri
- ☐ Head impulse testi
- ☐ Uzmanlık alanım olmadığı için değerlendirmeyi tercih etmiyorum
- ☐ Bilmiyorum
- ☐ Diğer

10.a. Diğer seçeneğini seçtiyseniz, lütfen açıklayın:

11. 6 aydan daha uzun süre önce travmatik beyin hasarına maruz kalmış hastanızı hangidurum veya durumlarda KBB ve/veya odyoloji departmanına sevk etmeyi düşünürsünüz? Birden fazla yanıt seçebilirsiniz.✱ *Required*

- ☐ Konuşmamı anlamadığını ve/veya duymadığını düşündüğümde hemen
- ☐ Dizziness ve/veya baş dönmesi gözlemlediğimde hemen
- ☐ Kulak çınlaması veya seslere karşı hassasiyet bildirirse hemen
- ☐ Durumunu fark etsem bile emin olmak için bir sonraki randevuyu beklerim
- ☐ Hasta herhangi bir şikayet bildirmedikçe sevk etmiyorum
- ☐ Bu hasta grubunun uğraştığı çok fazla problemi olduğu için görmezden gelinebilecek bir konu olduğunu düşünüyorum
- ☐ Bilmiyorum

12. 6 aydan daha uzun süre önce travmatik beyin hasarına maruz kalmış hastalarınızdantahmini olarak kaç tanesini odyoloji ve/veya KBB departmanına yönlendiriyorsunuz? ✱ *Required*

- ○ Hastalarımın hepsini
- ○ Hastalarımın yarısından
- ○ fazlasınıBazı hastalarımı
- ○ Birkaç hastamı
- ○ Hastalarımın hiçbirini yönlendirmiyorum
- ○ Bilmiyorum

13. 6 aydan daha uzun süre önce travmatik beyin hasarına maruz kalmış hastalarınızın gerektiğinde, dış kulaklarının durumunu nasıl kontrol edersiniz? ✱ *Required*

- ○ KBB hekimine ve/veya odyoloji departmanına konsülte ederim

- Otoskop ile kendim değerlendirmeyi tercih ederim
- Bu konuda şikayeti yoksa hiçbir şey yapmam
- Uzmanlık alanım olmadığı için ilgilenmiyorum
- Bilmiyorum

14. 6 aydan daha önce travmatik beyin hasarına maruz kalan hastanızın işitme kaybı varsaişitme cihazı kullanması gerektiğini düşünüyor musunuz? * Required

- Evet, kesinlikle kullanmalı
- İsterse, evet
- Gerek yok
- Çok fazla problemle uğraştıkları için görmezden gelinebilecek bir konu olduğunu düşünüyorum
- Bilmiyorum

15. İşitme cihazı kullanan travmatik beyin hasarına maruz kalmış hastalarınıza randevularısırasında işitme cihazlarıyla ilgili hangi durumlarda yardımcı olabilirsiniz? Birden fazla yanıt seçebilirsiniz. * Required

- İşitme cihazlarının pilini değiştirebilirim
- Hastamın işitme cihazının çalışıp çalışmadığını anlayabilirim
- Herhangi bir feedback (ötme sesi) sorunu varsa fark edebilirim
- İşitme cihazının kulak kalıbını ve/veya dome (kubbe) işlevini kontrol edebilirim
- İşitme cihazları hakkında hiçbir şey bilmiyorum

16. Travma sonrası sıklıkla işitsel ve/veya vestibüler şikayetler bildiren hasta grubunuz,travmatik beyin hasarının spesifik bir etiyolojisine sahip miydi? İşitsel ve/veya vestibüler şikayetleri olan bu hastaların travma etiyolojisini genelleyebilir misiniz ? * Required

- Evet

- Hayır
- Bilmiyorum

16.a. 17. soruya evet cevabı verdiyseniz, bu etiyoloji neydi?

- Düşme
- Trafik kazası
- Şiddet
- Spor yaralanmaları
- Diğer nedenler(Lütfen açıklaynız)

16.a.i. Diğer seçeneğini seçtiyseniz, lütfen açıklayın:

Supplemental Appendix 2. List of organisations that were contacted to share the surveys

Name of Organisations	Country
Speech Pathology Australia	Australia
Rehabilitation Medicine Society of Australia and New Zealand	Australia and New Zealand
the Royal Australian and New Zealand College of Psychiatrist	Australia and New Zealand
Speech-Language & Audiology Canada	Canada
Hellenic Neuropsychological Society	Greece
Logopedista	Italy
Association of Occupational Therapists of Ireland	Ireland
Irish Association of Speech & Language Therapists	Ireland
Headway The Brain Injury Association	United Kingdom
The British Neuropsychological Society	United Kingdom
British Psychological Society	United Kingdom
The British Neuropsychiatry Association	United Kingdom
Northampton Speech Therapy (Independent Therapist)	United Kingdom
British Society of Rehabilitation Medicine	United Kingdom
Royal College of Speech and Language Therapists	United Kingdom
Association of Speech and Language Therapists	Türkiye
World Federation of Occupational Therapists	

Supplemental Appendix 3. The results of statistical analysis on associations between audio-vestibular symptoms observed by HCPs or reported by patients' relatives to HCPs, and the years working with TBI adults, groups of country of residence and groups of occupations.

A statistically significant difference was observed in the years of working with TBI adults, in the case of 'being afraid to leave the house without an accompanying person because of their balance disorders' in the Kruskal-Wallis test ($p<0.05$). The statistical differences were identified between the groups working with adults with TBI for 5-10 years and 10-20 years (Appendix 3-Table 1), as well as between the groups working with adults with TBI for 5-10 years and more than 20 years in the Mann-Whitney U test with Bonferroni correction ($p<0.008$; Appendix 3-Table 2).

Ranks

	How many years have you been working with adult patients with traumatic brain injury?	N	Mean Rank	Sum of Ranks
Being afraid to leave the house without an accompanying person because of their balance disorders	5-10 years	20	14.88	297.50
	10-20 years	19	25.39	482.50
	Total	39		

Test Statistics[a]

	Being afraid to leave the house without an accompanying person because of their balance disorders
Mann-Whitney U	87.500
Wilcoxon W	297.500
Z	-2.970
Asymp. Sig. (2-tailed)	.003
Exact Sig. [2*(1-tailed Sig.)]	.003[b]

a. Grouping Variable: How many years have you been working with adult patients with traumatic brain injury?

Appendix 3-Table 1: The comparison of 5-10 years and 10-20 years groups in the case of 'being afraid to leave the house without an accompanying person because of their balance disorders' via the Mann-Whitney U test with Bonferroni correction

Ranks

	How many years have you been working with adult patients with traumatic brain injury?	N	Mean Rank	Sum of Ranks
Being afraid to leave the house without an accompanying person because of their balance disorders	5-10 years	20	13.75	275.00
	More than 20 years	15	23.67	355.00
	Total	35		

Test Statistics[a]

	Being afraid to leave the house without an accompanying person because of their balance disorders
Mann-Whitney U	65.000
Wilcoxon W	275.000
Z	-2.934
Asymp. Sig. (2-tailed)	.003
Exact Sig. [2*(1-tailed Sig.)]	.004[b]

a. Grouping Variable: How many years have you been working with adult patients with traumatic brain injury?

Appendix 3-Table 2: The comparison of 5-10 years and more than 20 years groups in the case of 'being afraid to leave the house without an accompanying person because of their balance disorders' via the Mann-Whitney U test with Bonferroni correction

Majority of HCPs (n=13, 19%) who work with TBI adults for 5-10 years stated that 'more than half' and 'some' of their patients reported 'being afraid to leave the house without an accompanying person because of their balance disorders', whilst most of the participants who work with TBI patients for 10-20 years (n=12, 17%) and more than 20 years (n=11, 16%) indicated that this case was observed in 'some' and 'a few' of their patients.

Furthermore, there were statistically significant differences for symptoms of 'asking to repeat their speech frequently during their interviews', 'relatives reporting complaints that the volume is too high while he/she is watching TV' and 'avoiding coming to appointments because TBI patients are concerned they will not understand what HCPs are talking about' based on groups of country of residence in the Kruskal-Wallis test (p<0.05). Statistically significant differences were noted between the UK and Asia for all three symptoms as seen in Appendix 3-Table 3.

Ranks

	What country do you work in?	N	Mean Rank	Sum of Ranks
Asks you to repeat your speech frequently during your interviews	Asia	15	15.00	225.00
	United Kingdom	30	27.00	810.00
	Total	45		
Relatives report complaints that the volume is too high while he/she is watching TV	Asia	15	12.87	193.00
	United Kingdom	30	28.07	842.00
	Total	45		
Avoids coming to appointments because they are concerned they will not understand what you are talking about	Asia	15	15.80	237.00
	United Kingdom	30	26.60	798.00
	Total	45		

Test Statistics[a]

	Asks you to repeat your speech frequently during your interviews	Relatives report complaints that the volume is too high while he/she is watching TV	Avoids coming to appointments because they are concerned they will not understand what you are talking about
Mann-Whitney U	105.000	73.000	117.000
Wilcoxon W	225.000	193.000	237.000
Z	-3.109	-3.803	-2.694
Asymp. Sig. (2-tailed)	.002	<.001	.007

a. Grouping Variable: What country do you work in?

Appendix 3-Table 3: The comparison of the UK and Asia groups in the cases of 'asking to repeat their speech frequently during their interviews', 'relatives reporting complaints that the volume is too high while he/she is watching TV' and 'avoiding coming to appointments because TBI patients are concerned they will not understand what HCPs are talking about' via the Mann-Whitney U test with Bonferroni correction

Similarly, differences were observed between Asia and Others for the symptoms 'asking to repeat their speech frequently during their interviews' and 'relatives reporting complaints that the volume is too high while he/she is watching TV' (p<0.008; Appendix 3-Table 4).

Ranks

	What country do you work in?	N	Mean Rank	Sum of Ranks
Asks you to repeat your speech frequently during your interviews	Asia	15	10.57	158.50
	Others	15	20.43	306.50
	Total	30		
Relatives report complaints that the volume is too high while he/she is watching TV	Asia	15	10.53	158.00
	Others	15	20.47	307.00
	Total	30		
Avoids coming to appointments because they are concerned they will not understand what you are talking about	Asia	15	12.33	185.00
	Others	15	18.67	280.00
	Total	30		

Test Statistics[a]

	Asks you to repeat your speech frequently during your interviews	Relatives report complaints that the volume is too high while he/she is watching TV	Avoids coming to appointments because they are concerned they will not understand what you are talking about
Mann-Whitney U	38.500	38.000	65.000
Wilcoxon W	158.500	158.000	185.000
Z	-3.197	-3.187	-2.045
Asymp. Sig. (2-tailed)	.001	.001	.041
Exact Sig. [2*(1-tailed Sig.)]	.001[b]	.001[b]	.050[b]

a. Grouping Variable: What country do you work in?

b. Not corrected for ties.

Appendix 3-Table 4: The comparison of the UK and Others groups in the cases of 'asking to repeat their speech frequently during their interviews', 'relatives reporting complaints that the volume is too high while he/she is watching TV' and 'avoiding coming to appointments because TBI patients are concerned they will not understand what HCPs are talking about' via the Mann-Whitney U test with Bonferroni correction

Additionally, a statistical difference was observed regarding the symptom 'relatives reporting complaints that the volume is too high while he/she is watching TV' between Europe and the UK in the Mann-Whitney U test with Bonferroni correction (p<0.008; Appendix 3-Table 5).

Ranks

	What country do you work in?	N	Mean Rank	Sum of Ranks
Asks you to repeat your speech frequently during your interviews	Europe	10	14.60	146.00
	United Kingdom	30	22.47	674.00
	Total	40		
Relatives report complaints that the volume is too high while he/she is watching TV	Europe	10	12.05	120.50
	United Kingdom	30	23.32	699.50
	Total	40		
Avoids coming to appointments because they are concerned they will not understand what you are talking about	Europe	10	15.75	157.50
	United Kingdom	30	22.08	662.50
	Total	40		

Test Statistics[a]

	Asks you to repeat your speech frequently during your interviews	Relatives report complaints that the volume is too high while he/she is watching TV	Avoids coming to appointments because they are concerned they will not understand what you are talking about
Mann-Whitney U	91.000	65.500	102.500
Wilcoxon W	146.000	120.500	157.500
Z	-2.013	-2.776	-1.542
Asymp. Sig. (2-tailed)	.044	.006	.123
Exact Sig. [2*(1-tailed Sig.)]	.067[b]	.007[b]	.140[b]

a. Grouping Variable: What country do you work in?

b. Not corrected for ties.

Appendix 3-Table 5: The comparison of the UK and Others groups in the cases of 'asking to repeat their speech frequently during their interviews', 'relatives reporting complaints that the volume is too high while he/she is watching TV' and 'avoiding coming to appointments because TBI patients are concerned they will not understand what HCPs are talking about' via the Mann-Whitney U test with Bonferroni correction

Most of participants in the UK (n=27, 39%) and Others (n=10, 14%) groups reported that the symptom of 'asking to repeat their speech frequently during their interviews' was observed in 'some' to 'a few' of their patients, most participants in Asia (n=13, 19%) reported that this symptom was observed in 'more than half' to 'some' of their patients. Most participants from Asia (n=7, 10%) and Europe (n=6, 9%) reported that 'some' of their patients' relatives complained about the volume being too high while they watch TV, whilst the majority of participants from the UK (n=15, 21%) mentioned that 'a few' of their patients' relatives reported this issue. Conversely, the majority of participants from the Others group (n=10, 14%) stated that 'a few' to 'none' of their patients' relatives reported this case. Most participants from Asia (n=6, 9%) reported that 'a few' of their patients avoided appointments because they were concerned that they would not understand what the HCPs were talking about. In contrast, most participants from the UK (n=11, 16%) reported that 'none' of their patients avoided appointments for this reason.

Another significant difference was observed between Groups 1 (physiotherapists) and 5 (doctors from other specialties) for the symptoms of 'relatives reporting complaints that the volume is too high while he/she is watching TV', 'avoiding coming to appointments because TBI patients are concerned they will not understand what HCPs are talking about', and 'an inability to tolerate certain sounds' in the Mann-Whitney U test with Bonferroni correction (p<0.003-Appendix 3-Table 6).

Test Statistics[a]

	Asks you to repeat your speech frequently during your interviews	Does not understand you when you are not facing her/him	Difficulty understanding you on the phone	Relatives report complaints that the volume is too high while he/she is watching TV	Avoids coming to appointments because they are concerned they will not understand what you are talking about	Reports an inability to tolerate certain sounds
Mann-Whitney U	76.500	73.000	45.000	44.500	34.000	46.000
Wilcoxon W	196.500	193.000	165.000	164.500	154.000	166.000
Z	-1.588	-1.713	-2.902	-2.949	-3.419	-2.996
Asymp. Sig. (2-tailed)	.112	.087	.004	.002	<.001	.002
Exact Sig. [2*(1-tailed Sig.)]	.137[b]	.106[b]	.004[b]	.004[b]	<.001[b]	.005[b]

a. Grouping Variable: What is your profession?

b. Not corrected for ties.

Appendix 3-Table 6: The comparison of the Groups 1and 5 in the cases of 'relatives reporting complaints that the volume is too high while he/she is watching TV', 'avoiding coming to appointments because TBI patients are concerned they will not understand what HCPs are talking about', and 'an inability to tolerate certain sounds' via the Mann-Whitney U test with Bonferroni correction

Most of the participants in Group 5 (n=8, 11%) stated that 'some' of their patients' relatives complained about the volume being too high while they watch TV, whilst the most participants of Group 1 (n=5, 7%) reported that 'none' of their patient relatives reported this problem. The majority of participants in Group 1 reported that 'none' of their TBI patients avoided coming to appointments because they were concerned, they would not understand what the HCPs were talking about. However, participants in Group 5 (n=4, 6% for each option) had equal percentages among the options of 'some', 'a few' and 'none' of their patients. The majority of participants in Group 1 (n=13, 19%) reported observing that 'some' to 'a few' of their patients reported an inability to tolerate certain sounds, whilst 17% of HCPs (n=12) in Group 5 reported observing this symptom in 'more than half' to 'some' of their patients.
