# Supplementary material for: Clinical experiences, current approaches, opinions and awareness of healthcare professionals regarding the audio-vestibular consequences of individuals with traumatic brain injury: a cross-sectional online survey study
Source: BMJ Open. 2024 Jul 8;14(7):e078017. doi: 10.1136/bmjopen-2023-078017 (PMC11256030; doi:10.1136/bmjopen-2023-078017)
Supplement: Author's manuscript [file bmjopen-2023-078017.draft_revisions.pdf]

BMJ Open

BMJ Open is committed to open peer review. As part of this commitment we make the peer review history of every article we publish publicly available.

When an article is published we post the peer reviewers' comments and the authors' responses online. We also post the versions of the paper that were used during peer review. These are the versions that the peer review comments apply to.

The versions of the paper that follow are the versions that were submitted during the peer review process. They are not the versions of record or the final published versions. They should not be cited or distributed as the published version of this manuscript.

BMJ Open is an open access journal and the full, final, typeset and author-corrected version of record of the manuscript is available on our site with no access controls, subscription charges or pay-per-view fees (http://bmjopen.bmj.com).

If you have any questions on BMJ Open's open peer review process please email info.bmjopen@bmj.com

BMJ Open

The Clinical Experiences and Opinions of Healthcare Professionals regarding the Audio-Vestibular Consequences of Individuals with Traumatic Brain Injury

Journal:	*BMJ Open*
Manuscript ID	bmjopen-2023-078017
Article Type:	Original research
Date Submitted by the Author:	21-Jul-2023

Complete List of Authors:	Bölükbaş, Kübra; University of Nottingham, Hearing sciences, Mental Health and Clinical Neuroscience, School of Medicine; NIHR Nottingham Biomedical Research Centre<br>Edwards, Laura; University Hospitals of Derby and Burton NHS Foundation Trust, Division of Rehabilitation Medicine; University of Nottingham, Injury, Inflammation and Recovery Sciences, School of Medicine<br>Baguley, David; University of Nottingham, Hearing sciences, Mental Health and Clinical Neuroscience, School of Medicine; University of Nottingham, NIHR Nottingham Biomedical Research Centre<br>Fackrell, Kathryn ; University of Nottingham, Hearing sciences, Mental Health and Clinical Neuroscience, School of Medicine; NIHR Nottingham Biomedical Research Centre

Keywords:	TRAUMA MANAGEMENT, Audiology < OTOLARYNGOLOGY, Surveys and Questionnaires, Patient-Centered Care, Physicians

SCHOLARONE™
Manuscripts

I, the Submitting Author has the right to grant and does grant on behalf of all authors of the Work (as defined in the below author licence), an exclusive licence and/or a non-exclusive licence for contributions from authors who are: i) UK Crown employees; ii) where BMJ has agreed a CC-BY licence shall apply, and/or iii) in accordance with the terms applicable for US Federal Government officers or employees acting as part of their official duties; on a worldwide, perpetual, irrevocable, royalty-free basis to BMJ Publishing Group Ltd ("BMJ") its licensees and where the relevant Journal is co-owned by BMJ to the co-owners of the Journal, to publish the Work in this journal and any other BMJ products and to exploit all rights, as set out in our licence.

The Submitting Author accepts and understands that any supply made under these terms is made by BMJ to the Submitting Author unless you are acting as an employee on behalf of your employer or a postgraduate student of an affiliated institution which is paying any applicable article publishing charge ("APC") for Open Access articles. Where the Submitting Author wishes to make the Work available on an Open Access basis (and intends to pay the relevant APC), the terms of reuse of such Open Access shall be governed by a Creative Commons licence – details of these licences and which Creative Commons licence will apply to this Work are set out in our licence referred to above.

Other than as permitted in any relevant BMJ Author's Self Archiving Policies, I confirm this Work has not been accepted for publication elsewhere, is not being considered for publication elsewhere and does not duplicate material already published. I confirm all authors consent to publication of this Work and authorise the granting of this licence.

For peer review only

The Clinical Experiences and Opinions of Healthcare Professionals regarding the Audio-Vestibular Consequences of Individuals with Traumatic Brain Injury

Kübra Bölükbaş[1,2], Laura Edwards[3,4], David M Baguley[1,2], Kathryn Fackrell[1,2,5]

[1]Hearing Sciences, Division of Mental Health and Clinical Neuroscience, School of Medicine, University of Nottingham, UK

[2]National Institute of Health and Social Research (NIHR) Nottingham Biomedical Research Centre, Nottingham, UK

[3]Division of Rehabilitation Medicine, University Hospitals of Derby and Burton NHS Foundation Trust, UK

[4]Injury, Inflammation and Recovery Sciences, School of Medicine, University of Nottingham, UK

[5]School of Healthcare Enterprise and Innovation, University of Southampton, UK

Corresponding author: Kübra Bölükbaş, NIHR Nottingham Biomedical Research Centre, Ropewalk House, 113 The Ropewalk, Nottingham, NG1 5DU, UK.

Email: kubra.bolukbas@nottingham.ac.uk

For peer review only

ABSTRACT

Objectives: To explore the experiences, current approaches, opinions, and awareness of healthcare professionals (HCPs) who care for adults with Traumatic Brain Injury (TBI) regarding the audio-vestibular consequences in these patients.

Design/setting: A cross-sectional online survey study

Participants: HCPs with experience of caring for adult patients with TBI, that were not ENT specialists or audiologists.

Methods: The survey study was conducted from May 2022 to December 2022. The online survey consisted of 16 closed and open-text questions in English and Turkish asking about clinical experience, current approaches and awareness of audio-vestibular consequences following a TBI. Frequencies of responses to closed questions were calculated. Open-text responses were reviewed and summarised.

Results: Seventy HCPs participated from 17 different professions and 14 different countries, with the majority from the UK. HCPs stated that 'some' to 'all' of their patients had auditory problems such as 'inability to understand speech in noise' (66%), 'tinnitus' (64%), 'hyperacusis' (57%), and balance problems such as 'dizziness' (79%) and 'vertigo' (67%). In general HCPs reported that they asked about the balance status of their patients at their appointments and that when they observed dizziness and/or balance disorder they used screening tests, most commonly Finger-to-Nose (53%). For auditory impairments, HCPs preferred to refer TBI patients to Audiology/ENT services, and 44% would suggest hearing aids to patients with hearing loss following a TBI 'if they would like to use them'.

Conclusions: A wide range of auditory-vestibular impairments is observed by HCPs caring for patients with TBI. The assessment and intervention decisions of HCPs for these impairments vary. However, non-expert HCPs may not be aware of negative consequences of untreated auditory-vestibular impairments following TBI. Therefore, it may be helpful to develop a simple framework for screening and indications of audio-vestibular impairments for onwards referral for non-audiological specialists who see this patient group regularly.

Keywords: Traumatic Brain Injury, Healthcare Professionals, Auditory, Vestibular, Balance, Survey

For peer review only

STRENGTHS AND WEAKNESSES OF THIS STUDY

1. This is the first study conducted with HCPs other than ENT specialist and audiologists on the audio-vestibular outcomes of patients with TBI.
2. Survey responses were received from HCPs all over the world, and as such the findings represent a range of experiences of assessing and managing audio-vestibular consequences in TBI patients across different countries and professions.
3. The inclusion of HCPs working in the balance field, such as physiotherapists, may have influenced the results, despite the absence of audiologists and ENT specialists.
4. This study has highlighted important areas for further study and practice, including education of HCPs around common audio-vestibular consequences of TBI, simple screening methods and routes for further assessment and treatment.

For peer review only

INTRODUCTION

Traumatic brain injury (TBI) is defined as a traumatic structural injury and/or deterioration of brain function following trauma due to an external force.[1] It is estimated that 64-74 million people every year experience a TBI[2] and it is one of the leading causes of death and disability worldwide.[3 4] There are many different complications associated with TBI, such as cognitive, emotional, behavioural, and audio-vestibular impairments.[5 6 7 8] In addition, neck pain, balance disorders and tinnitus (ringing in the ear(s)) have been reported as some of the most common complaints after head and neck trauma.[9 10] Moreover, in a review investigating hearing loss in TBI without skull fracture, Chen et al.[11] found that 58% of TBI patients experienced hearing loss, sometimes temporarily, despite not having any skull fractures. This rate suggests that the percentage of audio-vestibular consequences related to TBI that are not reported and/or investigated worldwide may be much higher than expected.

Consequences of TBI vary greatly by nature, severity and duration. Some individuals may be left with minimal impairment and make a rapid recovery with/without specialist intervention, whilst others may require inpatient or outpatient neurorehabilitation.[1] This diversity of complications due to TBI necessitates a multidisciplinary approach. Accordingly, depending on the severity and nature of the complication, a patient with TBI may involve a wide range of healthcare professionals (HCPs) including physiotherapists, psychologists, occupational therapists. The emergence of hearing loss and/or vestibular disorders, tinnitus, or hyperacusis (sound sensitivity) following TBI further complicates the treatment of TBI and requires a sensitive and careful approach in the diagnosis and treatment of audio-vestibular disorders in this patient group. Untreated hearing loss for example can lead to additional conditions such as dementia[12] as well as many psychological problems such as communication difficulties and social isolation.[13 14] Therefore, assessment and treatment of audio-vestibular conditions should not be neglected when caring for adults with TBI as early detection of audio-vestibular impairments in patients after TBI can improve quality of life, minimise complications and prevent additional conditions such as dementia.

Patients struggling with many problems such as physical, cognitive, emotional and behavioural complications after TBI may not easily notice any audio-vestibular impairments immediately. However, HCPs who care for them should understand and assess their audio-vestibular conditions, even if it is not their field of expertise and, if necessary, arrange referrals to the ENT (ear nose throat) specialists or audiologists. A recent survey on audio-vestibular clinical

For peer review only

practice within Australian audiology departments found gaps in audiologist knowledge and inadequate resources and practice regarding the identification, diagnosis and management of patients with auditory and/or vestibular deficits following TBI.[15] However, as yet, the clinical experiences, current approaches, opinions and awareness of HCPs who specialise in managing patients with TBI have not been fully investigated. It is therefore important to understand the experiences of these HCPs in particular to understand their awareness and knowledge of the audio-vestibular consequences of TBIs, in order to understand and identify areas that could be improved in existing practices.

Accordingly, the aim of this study was to explore and gain an understanding of the clinical experiences, current approaches, opinions, and awareness of the HCPs caring for adults with TBI (except ENT specialists and audiologists) regarding the audio-vestibular consequences experienced by these patients.

METHODS

Design

This study used a cross-sectional international online survey to explore the clinical experiences, current approaches, opinions and awareness of HCPs regarding the audio-vestibular conditions experienced by patients who had a TBI. Research approval was obtained from the University of Nottingham, Faculty of Medicine and Health Sciences Research Ethics Committee (FMHS 462-0222, May 2022). All respondents gave informed consent at the beginning of the survey. This survey study is reported according to Checklist for Reporting Results of Internet E-Surveys (CHERRIES).[16]

Survey Development

The survey questions were iteratively developed and refined through discussion and consensus within the study team. The authors initially compiled a list of potential questions and relevant sections for inclusion in the survey. Questions were based on experience of the authors and evidence from research. To ensure the questions and response options were relevant and valid for HCPs, the surveys were piloted with two HCPs and one researcher who have experience of audio-vestibular assessments. The questions were revised and distilled down to create a 16-question survey which included closed questions with single-choice, multiple-choice options, or Likert scales and for 4 questions with a free-text “other” option. Respondents were asked to

For peer review only

consider TBIs that occurred more than 6 months ago and the reported or observed audio-vestibular symptoms at that time. Responses to each question were required to complete the survey. It was possible for respondents to review and edit their responses until the point they chose to submit the survey on the final page. In order to reach a range of HCPs, and since the lead author (KB) is bilingual, two versions of the survey were created: one in English and one in Turkish (Supplemental appendix 1). The survey was delivered online using JISC software maintained by the University of Nottingham (https://www.onlinesurveys.ac.uk/).

Patient and Public Involvement

No patients were directly involved in the development, dissemination or reporting of this study. The study focused on the experiences of HCPs in the field of TBI, therefore the survey was piloted with HCPs and a researcher who have experience of audio-vestibular assessments to gain their views and refine questions, and two of the authors are clinicians with relevant experience in the field.

Study Sample

The study population was identified as HCPs caring for adult patients with TBI worldwide. However, ENT specialists and audiologists were not included in the study because they are already experts in the field of audio-vestibular care. In order to participate in the survey, all participants, except HCPs in Türkiye, needed the ability to understand and answer questions in English.

Distribution

A number of recruitment strategies were used to ensure a broad, representative sample of HCPs with a range of experiences, current approaches, opinions and awareness regarding audio-vestibular conditions in TBI patients. The survey invitation and link were circulated via professional organisations' contact lists including Headway UK, British Society of Physical and Rehabilitation Medicine, and Royal College of Speech and Language Therapists (see Supplemental appendix 2 for full list), promotion in blogs (e.g., Krysalis Neuro Occupational Therapy), and through social media channels (Facebook, Twitter, and Instagram). In addition, an invitation email with the survey link was sent to the HCPs identified via Expertscape website (https://expertscape.com/) as experts in the field of TBI. Responses were obtained between May 2022 and December 2022. Participation in the survey was voluntarily and no incentives were provided.

For peer review only

169 **Data analysis**

170 All survey data were imported to Microsoft Excel 2016©. Prior to analysis, data were checked to ensure all responses were complete. Descriptive statistics of closed question data (e.g., frequencies and percentages) were calculated in SPSS (v28). Open-text responses were reviewed and summarised in Microsoft Excel 2016©.

174 **RESULTS**

175 **Characteristics of participants**

176 The characteristics of respondents are reported in Table 1. A total of 70 HCPs completed the survey from 14 different countries. Of these, the majority were from the UK (n=30), followed by the Türkiye (n=14) and New Zealand (n=8). Respondents reported a range of occupations, including physiotherapists (n=15), occupational therapists (n=11) and speech and language therapists (n=10). The majority (48/70, 69%) had been in their occupation for more than 10 years and over half (54/70, 77%) had been working with TBI patients between 5 and 20 years. Over 50% spent more than 10 hours per week working with adult patients with TBI. Exactly half of the respondents worked in private healthcare (35/70, 50%), and the other half worked in NHS or state-owned hospitals.

185 **Table 1. Demographic information of participants**

Characteristics	n	%
**Country of residence**		
United Kingdom	30	42.9%
Türkiye	14	20.0%
New Zealand	8	11.5%
Ireland	6	8.6%
Canada	2	2.9%
Australia	2	2.9%
Greece	1	1.4%
United States of America	1	1.4%
Azerbaijan	1	1.4%
Italy	1	1.4%
Spain	1	1.4%
Croatia	1	1.4%
Venezuela	1	1.4%
Tanzania	1	1.4%
**Occupation**		

For peer review only

Physiotherapist	15	21.4%
Occupational Therapist	11	15.8%
Speech and Language Therapist	10	14.2%
Physiatrist or Rehabilitation Medicine Doctor	6	8.6%
Neuropsychiatrist	5	7.1%
Neuropsychologist	4	5.8%
Rehabilitation Nurse	4	5.8%
Neurologist	3	4.2%
Psychologist	2	2.9%
Geriatrician	2	2.9%
Neurosurgeon	2	2.9%
Optometrist	1	1.4%
Case Manager	1	1.4%
Anaesthetist	1	1.4%
Behavioural Scientist	1	1.4%
Primary Care Doctor	1	1.4%
Orthopaedic Surgeon	1	1.4%
**Years in occupation**		
Less than 5 years	8	11%
5 – 10 years	14	20%
10 – 20 years	25	36%
More than 20 years	23	33%
**Years working with TBI adults**		
Less than 5 years	16	23%
5 – 10 years	20	29%
10 – 20 years	19	27%
More than 20 years	15	21%
**Hours per week working with TBI adults**		
Less than an hour	7	10%
1 – 5 hours	13	19%
5 – 10 hours	10	14%
10 – 20 hours	15	21%
More than 20 hours	25	36%

The clinical experiences of HCPs regarding audio-vestibular consequences in TBI patients

This section explores the reported and/or observed audio-vestibular symptoms in adult patients with TBI. According to the results, the majority of responses ranged across 'a few' to 'most of my patients' describe the symptom (Figure 1). In general, HCPs reported that 'some' to 'all' of their patients had auditory problems such as 'inability to understand speech in noise' (46/70, 66%), 'tinnitus' (45/70, 64%), 'hyperacusis' (40/70, 57%), and 'inability to capture conversations' (44/70, 63%) and balance problems such as 'dizziness' (55/70, 79%), and

For peer review only

'vertigo' (47/70, 67%). However, the majority of HCPs observed that 'none' to 'a few' patients reported problems with 'inability to walk in the dark without a physical problem' (31/70, 44%), 'inability to understand speech in quiet' (40/70, 57%) and 'hearing loss' (41/70, 58%).

*** Insert Figure 1 here ***

The observed audio-vestibular symptoms by HCPs in patients with TBI (where trauma occurred more than 6 months ago) or reported to HCPs by patient relatives are shown in Figure 2. HCPs observed that 'some' to 'all' of their patient asks to 'repeat their speech frequently during their interviews' (45/70, 64%), 'difficulty understanding them on the phone' (38/70, 54%), and 'an inability to tolerate certain sounds' (44/70, 63%). In contrast, 37% of HCPs (26/70) did state that 'none' of their patients 'avoided coming to appointments because they were concerned, they will not understand what HCPs were talking about'. However, 17% (12/70) did not know whether there was such a concern for their patients. Over 40% (30/70) were unable to identify and generalise the aetiology of the TBI patients who frequently reported audio-vestibular complaints. Of the 21% (15/70) that were able, most reported that likely aetiologies were vehicle-related collisions (9/15, 60%), followed by falls (2/15, 13%) and sports injuries (1/15, 7%). Those who selected 'other' trauma option (3/15, 20%) indicated that audio-vestibular complaints could result from any TBI aetiology, including an object falling on the head, vehicle related collisions and/or sports related injuries.

*** Insert Figure 2 here ***

The current approaches to assess and manage audio-vestibular conditions of TBI patients

To understand the current approaches used by HCPs regarding the audio-vestibular conditions of their TBI patients, we asked questions about screening and assessment methods. Over half of respondents (52/70, 74%) indicated that they ask their TBI patients about their balance status (e.g., dizziness, vertigo), whilst the remaining stated that they only asked if their balance status was interfering with the conduct of the appointment (10/70, 14%), they did not ask if there are no complaints (5/70, 7%), or they preferred not to ask because it was not their expertise (1/70, 1%). When they noticed dizziness or balance disorder in TBI patients, the most commonly used screening methods were finger-to-nose (37/70, 53%), gait analysis (34/70, 49%), and eye movements (34/70, 49%) (Figure 3). In addition to these tests, HCPs also reported using evaluation methods such as videonystagmography (VNG) and dynamic visual acuity. Again, several HCPs reported that they prefer not to use any screening method because it was not their area of expertise (23/70, 33%). In relation to checking the external ear condition of TBI

For peer review only

patients, almost half of the respondents (34/70, 49%) stated that they referred patients to the ENT or audiology department, whilst 13% (9/70) reported that they evaluated it themself with an otoscope.

*** Insert Figure 3 here ***

When asked how many TBI patients they referred to ENT and/or audiology, 78% (55/70) of HCPs said that they referred ‘some’ or ‘a few’ of their TBI patients. Only 4% (3/77) of HCPs referred all of their TBI patients to ENT and/or audiology department. HCPs stated that they considered referring TBI patients to the ENT specialist and/or audiology department, when their patients could not understand and/or hear their speech (37/70, 53%), when their patients reported tinnitus and hyperacusis (33/70, 47%) or when they observed vertigo and/or dizziness in their patients (29/70, 41%). Only 6% (4/70) felt that audio-vestibular conditions could be ignored because TBI patients struggled with many impairments (Figure 4). In contrast, when asked whether TBI patients should use a hearing aid if they have hearing loss, no one thought it was something that could be ignored. However, most of the respondents (31/70, 44%) stated that TBI patients with hearing loss should use hearing aids ‘if they would like to’, rather than 'definitely', whilst the remaining did not know (14/70, 20%). When asked if they could help their TBI patients using hearing aids at their appointments, interestingly whilst 40% (28/70) stated that they knew did not know anything about hearing aids, 40% (28/70) also stated that they could change the battery of the hearing aid. The majority (32/70, 46%), however, did state that they would notice whether the hearing aid was working.

*** Insert Figure 4 here ***

DISCUSSION

Our study explored the clinical experiences, current approaches, opinions, and awareness of HCPs of audio-vestibular consequences in TBI patients. In general, the majority of HCPs had observed audio-vestibular impairments in their TBI patients in line with the literature.[17,18,19] In our study, most HCPs reported that hearing loss was only observed in ‘a few’ of patients. In contrast, a systematic review study showed that sensorineural hearing loss was the most frequently observed auditory impairment at a rate of 37.3% in patients with non-blast related TBI.[17] Moreover, it has been shown that following TBI, individuals can experience auditory symptoms even if their hearing thresholds are within the normal range (≤25 dB).[20] Consequently, these studies indicate that hearing loss can be observed or reported by TBI

patients more often than HCPs in our study observed or detected it in their patients. One potential explanation for these differences could be that the HCPs who participated in this study are not experts in audio-vestibular assessments and as such may not be looking for audiological impairments, particularly during appointments which may be addressing other concerns. However, recognition of hearing loss in TBI patients by non-audiological-specialist HCPs may result in more onwards referrals for detailed assessment and input, resulting in better outcomes.

We also observed that HCPs were more likely to assess their TBI patient's vestibular and/or balance-related conditions than their hearing-related conditions. One explanation for this is that different HCPs such as neurologists, physiotherapists and occupational therapists, who participated in our study, may be more likely to assess and treat balance and/or vestibular function, and less likely to be familiar with auditory assessments. This might be a possible reason why patients with auditory problems were more likely to be referred.

However, in contrast, a number of HCPs did not refer to ENT or audiology services if the patient had not complained about auditory problems and/or if they felt that the audio-vestibular problems were an issue that were not a priority compared to other TBI-related impairments. This suggests that some HCPs may not be aware of the negative short-term and long-term consequences of untreated audio-vestibular impairments. Many HCPs reported that they would leave the use of a hearing aid to the discretion of individual patients. This is understandable, in that patients who do not want to use a hearing aid are unlikely to use one, even if it is provided. However, it may also reflect lack of training and education in hearing aids for HCPs. It is important that HCPs explore the patient's rationale and capacity [21] for making this decision as well as explaining the potential consequences of untreated hearing loss.[12]

Finally, consistent with the studies in the literature[22 15], several HCPs reported that TBI patients with audio-vestibular complaints had the most vehicle-related collision aetiology. However, this was not universal and likely reflects the fact that audio-vestibular complaints are a common consequence of a wide variety of TBI aetiologies.

Interestingly, a recent Australian study [15] also showed that audiologists lacked knowledge about the assessment and management of auditory-vestibular impairments of TBI patients in audiological services. Given that our study demonstrated a similar lack of awareness regarding the auditory-vestibular consequences of TBI patients in non-audiology HCPs, it becomes evident that there is a need for greater awareness among HCPs working with this patient

For peer review only

population, even if it is outside their area of expertise. For this purpose, it may be beneficial to prepare a practical audiology guide for all HCPs dealing with TBI in future studies.

Strengths and limitations

To our knowledge, this is the first study to investigate the clinical experiences, opinions, awareness and current approaches of HCPs, other than ENT specialists and audiologists, regarding the audio-vestibular consequences of TBI patients. This international survey received responses from a total of 70 participants from 17 different departments and 14 countries and as such we have gained knowledge from a range of HCPs who are actively working in clinical settings that assess and manage audio-vestibular consequences in TBI patients. However, we do recognise that the majority were from the UK which means that some of the findings may reflect the UK health system. For example, other countries may have audiologists/ENTs as part of their multi-disciplinary team. Furthermore, the inclusion of HCPs such as physiotherapists working in the field of balance may have influenced the results, in particular the confidence in vestibular assessments. Finally, we wanted to understand the experience of HCPs who specialise in TBI therefore we did not include audiologists and/or ENT specialists in this survey and as such we do not know their experiences with this patient population, except for the findings from the Australia survey[15] which indicate similar experiences to our findings here. Future research should consider further investigation into the experiences of audiologists/ENTs in relation to this patient population, in particular the assessments that should be carried out.

Conclusion

HCPs who care for patients who sustained TBI observe a wide range of auditory-vestibular outcomes in these patients. HCP decisions around further assessment, investigation and treatment were variable in this study. Untreated audio-vestibular impairments may have significant adverse consequences for patients and non-expert clinicians may be unaware of these. We suggest that standardised screening protocols may enable easier identification of audio-vestibular problems with signposting to appropriate specialties for further assessment and intervention. Thus, audio-vestibular impairments of TBI patients can be diagnosed and treated earlier, to improve quality-of-life of TBI patients.

For peer review only

ACKNOWLEDGEMENTS

The authors wish to acknowledge the clinicians who piloted the survey and all the respondents of the survey. We would like to express our gratitude to Professor David Baguley, who sadly passed away during this study. His knowledge and expertise helped develop the fundamental ideas for this research, for which we are very thankful.

AUTHOR STATEMENT

DB, LE, KB authors conceptualised the project and design of the survey questions. KB led the recruitment and data collection, supported by KF, DB, LE. KF, DB and LE informed methodology and interpretation of analysis. KB conducted initial analysis. KF and LE reviewed and refined analysis. KB drafted manuscript. All authors reviewed and approved the final manuscript for submission.

FUNDING

This work was supported by the Ministry of National Education of the Republic of Türkiye and the National Institute for Health Research (NIHR) Nottingham Biomedical Research Centre.

DISCLAIMER

KB was funded by the Ministry of National Education of the Republic of Türkiye to undertake this work as part of her PhD. KF was funded by National Institute for Health Research (NIHR Post-Doctoral Fellowship, PDF-2018-11-ST2-003) at the time of completing this work. The views expressed in this publication are those of the author (s) and not necessarily those of the NIHR, the NHS or the Department of Health and Social Care.

COMPETING INTERESTS

None declared

PATIENT CONSENT FOR PUBLICATION

Not required

For peer review only

ETHICS APPROVAL

Research approval was obtained from the University of Nottingham, Faculty of Medicine and Health Sciences Research Ethics Committee (FMHS 462-0222, May 2022). All respondents gave informed consent at the beginning of the survey.

PROVENANCE AND PEER REVIEW

Not commissioned; externally peer reviewed.

DATA SHARING STATEMENT

Data are available upon reasonable request.

ORCID IDS

Kübra Bölükbaş https://orcid.org/0000-0002-7625-4758

Laura Edwards https://orcid.org/0000-0003-1392-2648

Kathryn Fackrell http://orcid.org/0000-0001-6529-8643

TWITTER

Kubra Bolukbas @_kubrabolukbas_, Kathryn Fackrell @FackrellKathryn

For peer review only

REFERENCES

1. NICE. Head injury briefing paper. 2014 [cited 2023 Jan 2];77. Available from: https://www.nice.org.uk/guidance/qs74/documents/head-injurybriefing- paper2.
2. Dewan MC, Rattani A, Gupta S, Baticulon RE, Hung YC, Punchak M, et al. Estimating the global incidence of traumatic brain injury. J Neurosurg. 2019 Apr 1;130(4):1080–97.
3. Maas AIR, Menon DK, Steyerberg EW, Citerio G, Lecky F, Manley GT, et al. Collaborative European NeuroTrauma Effectiveness Research in Traumatic Brain Injury (CENTER-TBI): A Prospective Longitudinal Observational Study. Neurosurgery [Internet]. 2015;76(1). Available from: https://journals.lww.com/neurosurgery/Fulltext/2015/01000/Collaborative_European_NeuroTrauma_Effectiveness.8.aspx
4. Rubiano AM, Carney N, Chesnut R, Puyana JC. Global neurotrauma research challenges and opportunities. Vol. 527, Nature. Nature Publishing Group; 2015. p. S193–7.
5. Ahmed S, Venigalla H, Mekala HM, Dar S, Hassan M, Ayub S. Traumatic brain injury and neuropsychiatric complications. Vol. 39, Indian Journal of Psychological Medicine. Medknow Publications; 2017. p. 114–21.
6. Konrad C, Geburek AJ, Rist F, Blumenroth H, Fischer B, Husstedt I, et al. Long-term cognitive and emotional consequences of mild traumatic brain injury. Psychol Med. 2011 Jun;41(6):1197–211.
7. Hoover EC, Souza PE, Gallun FJ. Auditory and cognitive factors associated with speech-in-noise complaints following mild traumatic brain injury. In: Journal of the American Academy of Audiology. American Academy of Audiology; 2017. p. 325–39.
8. Campbell KR, Parrington L, Peterka RJ, Martini DN, Hullar TE, Horak FB, et al. Exploring persistent complaints of imbalance after mTBI: Oculomotor, peripheral vestibular and central sensory integration function. Journal of Vestibular Research. 2021;31:519–30.
9. Kreuzer PM, Landgrebe M, Schecklmann M, Staudinger S, Langguth B. Trauma-Associated Tinnitus: Audiological, Demographic and Clinical Characteristics. PLoS

For peer review only

One [Internet]. 2012 Sep 26;7(9):e45599-. Available from: https://doi.org/10.1371/journal.pone.0045599

10. Elzière M, Devèze A, Bartoli C, Levy G. Post-traumatic balance disorder. Vol. 134, European Annals of Otorhinolaryngology, Head and Neck Diseases. Elsevier Masson SAS; 2017. p. 171–5.

11. Chen JX, Lindeborg M, Herman SD, Ishai R, Knoll RM, Remenschneider A, et al. Systematic review of hearing loss after traumatic brain injury without associated temporal bone fracture. Am J Otolaryngol. 2018 May 1;39(3):338–44.

12. Livingston G, Huntley J, Sommerlad A, Ames D, Ballard C, Banerjee S, et al. Dementia prevention, intervention, and care: 2020 report of the Lancet Commission. The Lancet [Internet]. 2020 Aug 8;396(10248):413–46. Available from: https://doi.org/10.1016/S0140-6736(20)30367-6

13. Wilson BS, Tucci DL. Addressing the global burden of hearing loss. The Lancet [Internet]. 2021 Mar 13;397(10278):945–7. Available from: https://doi.org/10.1016/S0140-6736(21)00522-5

14. Monzani D, Galeazzi GM, Genovese E, Marrara A, Martini A. Psychological profile and social behaviour of working adults with mild or moderate hearing loss. Acta Otorhinolaryngologica Italica. 2008;28(2):61–6.

15. Šarkić B, Douglas JacintaM, Simpson A. A cross-sectional survey of non-specialist Australian audio-vestibular clinical practice for traumatic brain injury and rehabilitation. Brain Impairment [Internet]. 2023 Jan 6 [cited 2023 Jan 13];1–18. Available from: https://www.cambridge.org/core/product/identifier/S1443964622000353/type/journal_article

16. Eysenbach G. Improving the quality of web surveys: The Checklist for Reporting Results of Internet E-Surveys (CHERRIES). Vol. 6, Journal of Medical Internet Research. JMIR Publications Inc.; 2004.

17. Šarkić B, Douglas JacintaM, Simpson A. Peripheral auditory dysfunction secondary to traumatic brain injury: a systematic review of literature. Brain Inj [Internet]. 2019 Jan 28;33(2):111–28. Available from: https://doi.org/10.1080/02699052.2018.1539868

18. Roup CM, Ross C, Whitelaw G. Hearing difficulties as a result of traumatic brain injury. J Am Acad Audiol. 2020;31(2):137–46.

For peer review only

19. Šarkić B, Douglas JM, Simpson A, Vasconcelos A, Scott BR, Melitsis LM, et al. Frequency of peripheral vestibular pathology following traumatic brain injury: a systematic review of literature. Vol. 60, International Journal of Audiology. Taylor and Francis Ltd.; 2020. p. 479–94.

20. Knoll RM, Lubner RJ, Brodsky JR, Wong K, Jung DH, Remenschneider AK, et al. Auditory Quality-of-Life Measures in Patients With Traumatic Brain Injury and Normal Pure Tone Audiometry. Otolaryngology - Head and Neck Surgery (United States). 2020 Dec 1;163(6):1250–4.

21. Cotrena C, Branco LD, Zimmermann N, Cardoso CO, Grassi-Oliveira R, Fonseca RP. Impaired decision-making after traumatic brain injury: The Iowa Gambling Task. Brain Inj [Internet]. 2014 Jul 1;28(8):1070–5. Available from: https://doi.org/10.3109/02699052.2014.896943

22. Peeters W, van den Brande R, Polinder S, Brazinova A, Steyerberg EW, Lingsma HF, et al. Epidemiology of traumatic brain injury in Europe. Acta Neurochir (Wien) [Internet]. 2015;157(10):1683–96. Available from: https://doi.org/10.1007/s00701-015-2512-7

FIGURE LEGENDS

Figure 1. The frequency of audio-vestibular symptoms reported by patients to HCPs

Figure 2. The frequency of audio-vestibular symptoms reported by patients or their relatives to HCPs and/or observed by HCPs

Figure 3. Screening methods used by HCPs when they notice dizziness or balance disorders in patients with TBI

Figure 4. Reasons for HCPs to refer TBI patients to the ENT and/or audiology department.

SUPPLEMENTAL MATERIALS

Supplemental Appendix 1. English and Turkish versions of the survey

Supplemental Appendix 2. List of organisations that were contacted to share the surveys

For peer review only

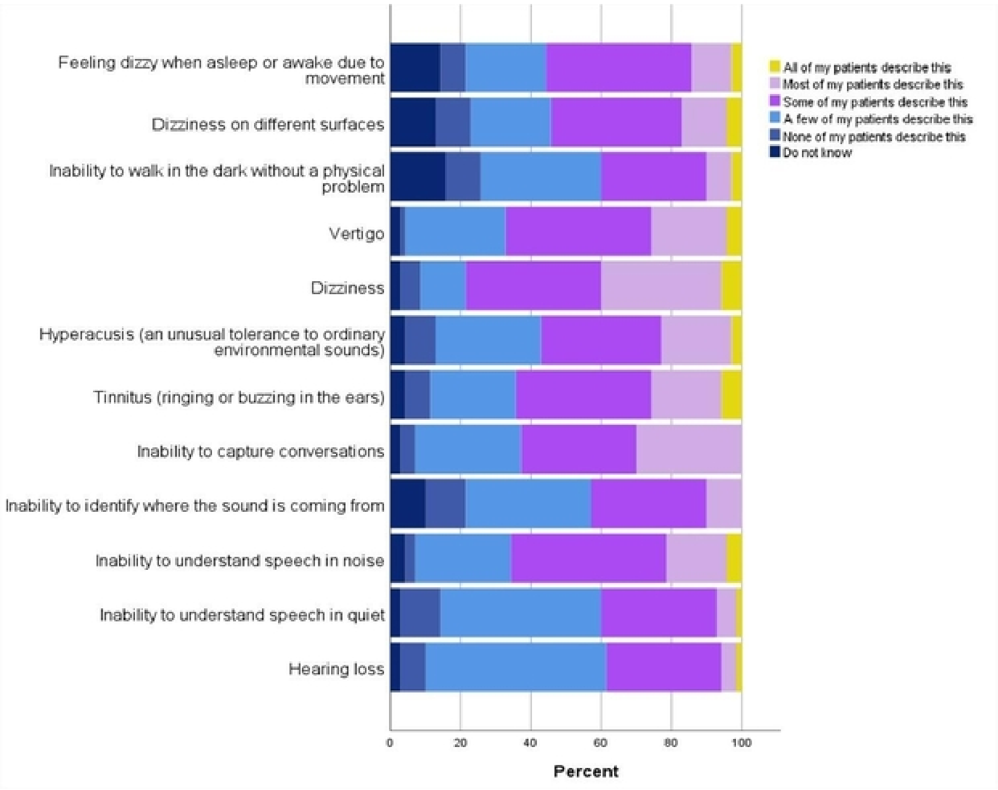

Figure 1. The frequency of audio-vestibular symptoms reported by patients to HCPs

52x41mm (300 x 300 DPI)

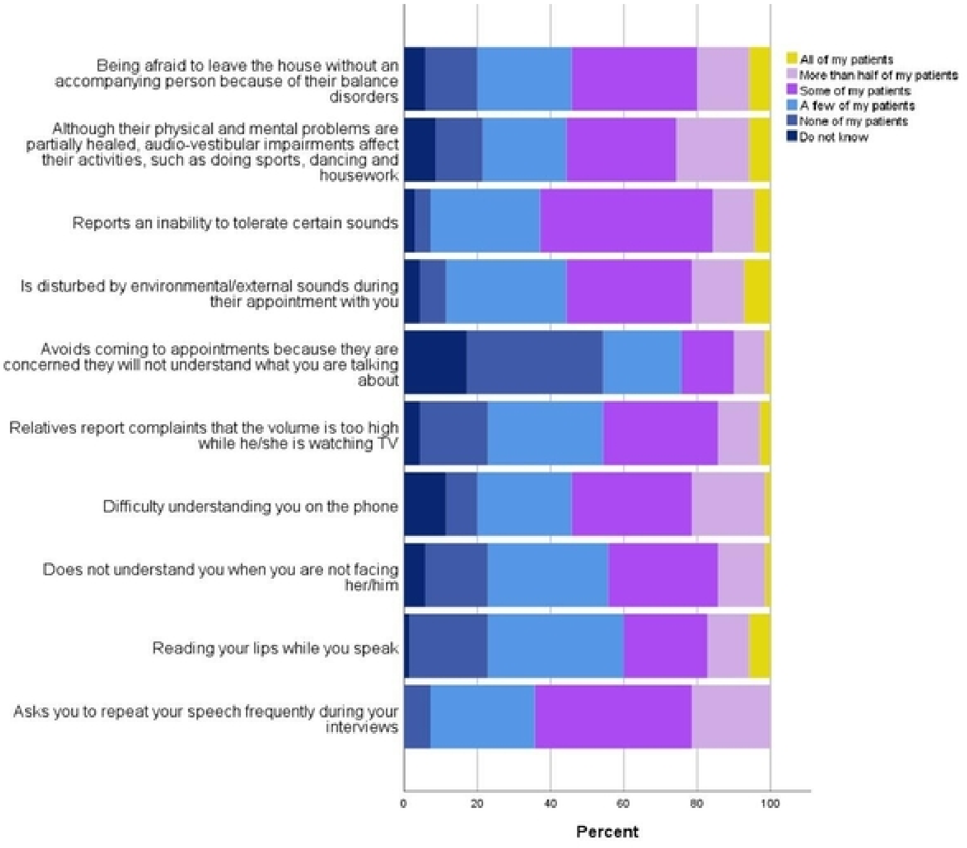

Figure 2. The frequency of audio-vestibular symptoms reported by patients or their relatives to HCPs and/or observed by HCPs

51x43mm (300 x 300 DPI)

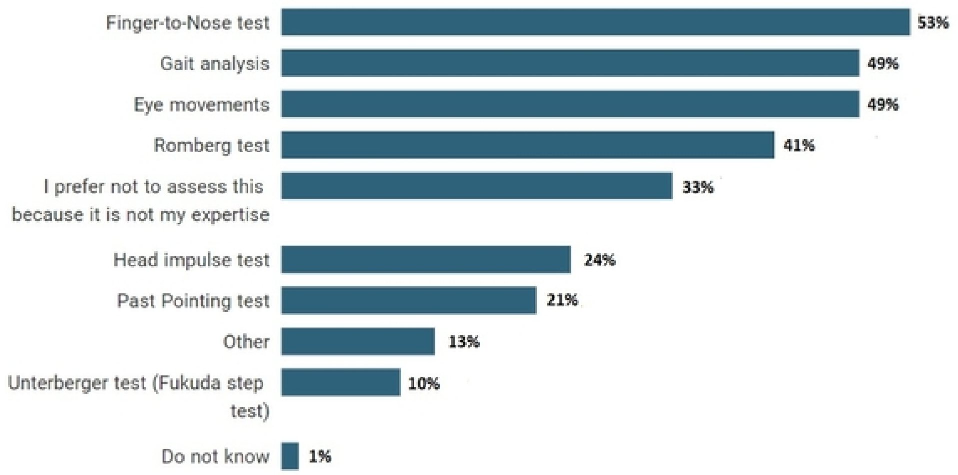

Figure 3. Screening methods used by HCPs when they notice dizziness or balance disorders in patients with TBI

50x24mm (300 x 300 DPI)

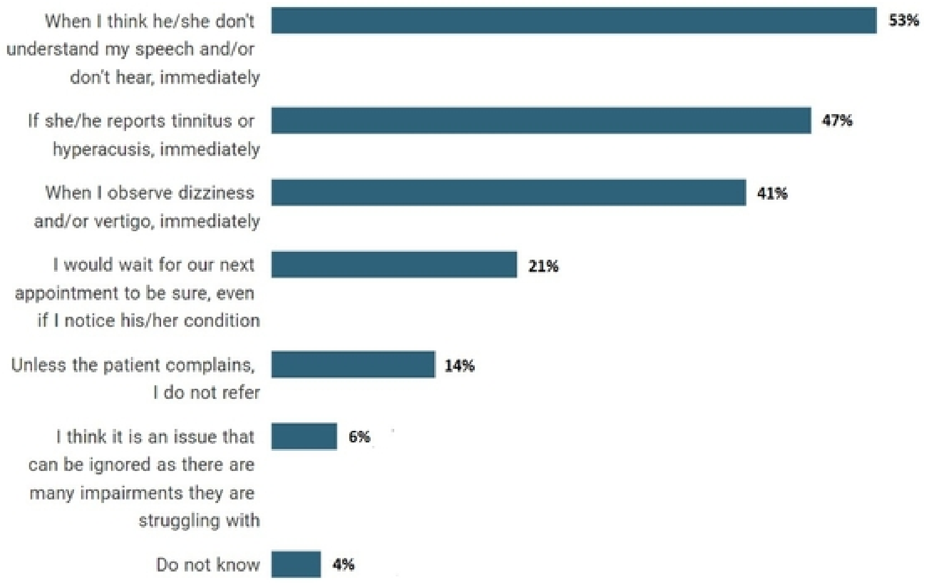

Figure 4. Reasons for HCPs to refer TBI patients to the ENT and/or audiology department.

50x30mm (300 x 300 DPI)

Supplemental Appendix 1. English and Turkish version of the survey

English Version of Survey

Healthcare professionals and Audio- vestibular consequences of Traumatic Brain Injury

Page 1: Information and Consent Page for an OnlineSurvey/Questionnaire

Information and Consent page for an Online Survey/Questionnaire

Study Title: Experiences and opinions of healthcare professionals about the audio-vestibular findings of individuals with traumatic brain injury

Research Team:

Name of supervisors:

Kathryn Fackrell, Senior Research Fellow

Laura Edwards, Clinical Associate Professor in the Faculty of Medicine and Health Sciences

Co-investigator:

Kubra Bolukbas, PhD in Hearing Sciences

Faculty of Medicine and Health Sciences Research Ethics Ref: FMHS 462-0222

For peer review only

This study investigates the opinions, experiences and approaches of healthcare professionals ***(excluding ENT/audiology specialists)*** who deal with adults with traumatic brain injury, with a focus on the auditory and balance status of these patients.

For peer review only

Thank you for your interest. You are invited to take part because you are a healthcare professional ***(excluding ENT/audiology specialists)*** who works with adult traumatic brain injury patients. Please read through the following information before agreeing to participate. You can ask any questions before deciding by contacting the researchers (details below). Taking part is entirely voluntary.

What will I be asked to do?

After clicking the ‘next’ button at the end of this information page you will be asked to provide informed consent to participate in this study. You will then be asked to provide some basic demographic information related to your profession (i.e. profession, years of occupation, type of institution) and will also answer some questions about your experiences, opinions, and approaches in relation to audio-vestibular findings of traumatic brain injury patients. It should take you a maximum of 20 minutes to complete. We would like you to answer all questions as honestly and completely as possible. You can withdraw at any point during the questionnaire for any reason, before submitting your answers by clicking the Exit button/closing the browser. The data will only be uploaded on completion of the questionnaire by clicking the FINISH button on the final page. At this point, it will not be possible to withdraw your answers.

What are the disadvantages of taking part?

We are asking you to give up your valuable time to take part and this is appreciated.

What are the advantages of taking part?

Your contribution, along with others, will contribute to researchers’ understanding of the awareness of healthcare professionals about the auditory and balance status of patients with traumatic brain injury. It will also contribute to the understanding of the international determination of the approaches to audio-vestibular problems in these patients. Future research may also be conducted based on the results of this study.

Who will know I have taken part in the study?

No one will know you have taken part in this study because we will not ask for your name or any other personal identification details (ID) during this survey. Your IP address will not be visible to or stored by the research team because an online survey platform is being used which receives and stores an IP address but enables this detail to be filtered out before it is transferred to the research team. As with any online related activity, the risk of breach is possible but this risk is being minimised by using a platform on an encrypted webpage.

For peer review only

What will happen to your data?

When you have clicked the submit button at the end of the questionnaire, it will be uploaded into a password-protected database with a code number. The research team will not be able to see who it is from and for this reason, it will not be possible to withdraw the data at this point. Your data (research data) will be stored in a password-protected folder on a restricted-access server at the university under the terms of its data protection policy. Data is kept for a minimum of 7 years and then destroyed.

This survey is for a PhD project and the answers received from all participants will be combined in a password-protected database ready for analysis. The results will be written up as a dissertation and may be used in academic publications and presentations. The overall anonymised data from this study may be shared for use in future research and teaching (with research ethics approval).

The only personal data we will receive is your e-mail if you contact us to ask further questions or need support. This will be received and handled separately from your completed questionnaire and it will not be possible to link the sets of data. Your e-mail address will only be kept as long as needed to resolve your problem. It will then be destroyed. For further information about how the university processes personal data please see: https://www.nottingham.ac.uk/utilities/privacy.aspx/

Who will have access to your data?

The University of Nottingham is the data controller (legally responsible for data security) and the Supervisor of this study (named above) is the data custodian (manages access to the data) and as such will determine how your data is used in the study. Your research and personal data will be used for the purposes of the research only. Research is a task that we perform in the public interest.

Responsible members of the University of Nottingham may be given access to data for monitoring and/or audit of the study to ensure it is being carried out correctly.

If you have any questions or concerns about this project, please contact:

Kubra Bolukbas, E-mail: kubra.bolukbas@nottingham.ac.uk or

if you have any concerns about any aspect of this study please contact the Research Supervisor:

Kathryn Fackrell, Email: kathryn.fackrell@nottingham.ac.uk;

Laura Edwards, Email: laura.edwards@nottingham.ac.uk

For peer review only

If you remain unhappy and wish to complain formally, you should then contact the FMHS Research Ethics Committee Administrator E-mail:

FMHS-ResearchEthics@nottingham.ac.uk

For peer review only

Page 2: Consent Form

Please confirm each box to continue. If you do not accept the consent form, please close the browser. ✱ *Required*

Please select at least 5 answer(s).

- [ ] I confirm that I have read and understood the information on the previous page
- [ ] I am a healthcare professionals dealing with adult traumatic brain injury patients (other than audiologist and/or ENT specialist)
- [ ] I understand that my participation is voluntary and I can end the study at any time and withdraw my data by clicking the EXIT button
- [ ] I understand that my answers are anonymous
- [ ] I understand the overall anonymized data from this study may be used in the future for research (with research ethics approval) and teaching purposes

For peer review only

Page 3: Experiences and opinions of healthcare professionals about the audio-vestibular findings of individuals with traumatic brain injury

1. What is your profession? * Required

- Neurologist
- Neuropsychiatrist
- Neurosurgeon
- General Surgeon
- Physiatrist or Rehabilitation Medicine Doctor
- Geriatrician
- Orthopaedic Surgeon
- Rehabilitation Nurse
- Neuropsychologist
- Speech and Language Therapist
- Occupational Therapist
- Physiotherapist
- Psychologist
- Other

1.a. If you selected Other, please specify:

2. How many years have you been in your occupation? * Required

- Less than 5 years
- 5-10 years
- 10-20 years

- More than 20 years

3. How many years have you been working with adult patients with traumatic brain injury? * *Required*

- Less than 5 years
- 5-10 years
- 10-20 years
- More than 20 years

4. What country do you work in? * *Required*

- Australia
- Azerbaijan
- Belgium
- Canada
- China
- Cyprus
- Denmark
- France
- Germany
- Greece
- India
- Ireland
- Israel
- Italy
- Japan
- Netherlands
- Poland

- Saudi Arabia
- Spain
- Sweden
- Switzerland
- Thailand
- Türkiye
- United Kingdom
- United States of America
- Other

4.a. If you selected Other, please specify:

5. Is your place of work private or state-owned? * *Required*

- Private
- State-owned

6. How many hours per week do you spend working with traumatic brain injury adult patients? * *Required*

- Less than an hour
- 1-5 hours
- 5-10 hours
- 10-20 hours
- More than 20 hours

For peer review only

7. The following symptoms may be reported by people who have had a traumatic brain injury. Please indicate how frequently your adult patients, who experienced trauma more than 6 months ago, describe these to you ✱ *Required*

Please don't select more than 1 answer(s) per row.

Please select at least 12 answer(s).

	All of my patients describe this	Most of my patients describe this	Some of my patients describe this	A few of my patients describe this	None of my patients describe this	Do not know
Hearing loss	☐	☐	☐	☐	☐	☐
Inability to understand speech in quiet	☐	☐	☐	☐	☐	☐
Inability to understand speech in noise	☐	☐	☐	☐	☐	☐
Inability to identify where the sound is coming from	☐	☐	☐	☐	☐	☐
Inability to capture conversations	☐	☐	☐	☐	☐	☐
Tinnitus (ringing or buzzing in the ears)	☐	☐	☐	☐	☐	☐
Hyperacusis (an unusual tolerance to ordinary environmental sounds)	☐	☐	☐	☐	☐	☐
Dizziness	☐	☐	☐	☐	☐	☐
Vertigo	☐	☐	☐	☐	☐	☐

Inability to walk in the dark without a physical problem	☐	☐	☐	☐	☐	☐
Dizziness on different surfaces	☐	☐	☐	☐	☐	☐
Feeling dizzy when asleep or awake due to movement	☐	☐	☐	☐	☐	☐

For peer review

8. The following symptoms may be reported and/or observed by people who have had a traumatic brain injury. Please indicate how frequently, adults who experienced trauma more than 6 months ago, describe these to you, ✱*Required*

Please don't select more than 1 answer(s) per row.

Please select at least 10 answer(s).

	All of my patients	More than half of my patients	Some of my patients	A few of my patients	None of my patients	Do not know
Asks you to repeat your speech frequently during your interviews	☐	☐	☐	☐	☐	☐
Reading your lips while you speak	☐	☐	☐	☐	☐	☐
Does not understand you when you are not facing her/him	☐	☐	☐	☐	☐	☐
Difficulty understanding you on the phone	☐	☐	☐	☐	☐	☐

For peer review only - http://bmjopen.bmj.com/site/about/guidelines.xhtml

Relatives report complaints that the volume is too high while he/she is watching TV	☐	☐	☐	☐	☐	☐
Avoids coming to appointments because they are concerned they will not understand what you are talking about	☐	☐	☐	☐	☐	☐
Is disturbed by environmental/external sounds during their appointment with you	☐	☐	☐	☐	☐	☐
Reports an inability to tolerate certain sounds	☐	☐	☐	☐	☐	☐
Although their physical and mental problems are partially healed, audio-vestibular impairments affect their activities, such as doing sports, dancing and housework	☐	☐	☐	☐	☐	☐
Being afraid to leave the house without an accompanying person because of their balance disorders	☐	☐	☐	☐	☐	☐

9. Do you ask your adult patients with traumatic brain injury (which occurred more than 6 months ago) about their balance status (dizziness, vertigo etc.) at your appointments? ✱ *Required*

- Yes, I ask everybody
- If their balance status prevents me from performing our appointments and/or my job, I ask
- I prefer not to ask because it is not my expertise
- I do not ask anything if there are no complaints about it
- Do not know

10. If you notice dizziness or balance disorders in your adult patients with traumatic brain injury (which occurred more than 6 months ago), which of the screening methods would you use? You can choose multiple options *✱ Required*

- [ ] Past Pointing test
- [ ] Finger-to-Nose test
- [ ] Unterberger test (Fukuda step test)
- [ ] Romberg test
- [ ] Gait analysis
- [ ] Eye movements
- [ ] Head impulse test
- [ ] I prefer not to assess this because it is not my expertise
- [ ] Do not know
- [ ] Other

10.a. If you selected Other, please specify:

11. In which situation would you consider referring your traumatic brain injury patient (where the trauma occurred more than 6 months ago) to the ENT and/or audiology service? You can choose multiple options *✱ Required*

For peer review only - http://bmjopen.bmj.com/site/about/guidelines.xhtml

- ☐ When I think he/she don't understand my speech and/or don’t hear, immediately
- ☐ When I observe dizziness and/or vertigo, immediately
- ☐ If she/he reports tinnitus or hyperacusis, immediately
- ☐ I would wait for our next appointment to be sure, even if I notice his/her condition
- ☐ Unless the patient complains, I do not refer
- ☐ I think it is an issue that can be ignored as there are many impairments they are struggling with
- ☐ Do not know

12. How many of your adult patients who have had a traumatic brain injury (which occurred more than 6 months ago) do you refer to the audiology or ENT department? * *Required*

- ○ All of my patients
- ○ More than half of my patients
- ○ Some of my patients
- ○ A few of my patients
- ○ None of my patients
- ○ Do not know

13. How do you check the external ear condition of your adult patients with traumatic brain injury (which occurred more than 6 months ago)? * *Required*

- ○ Referring to the ENT and/or audiology service
- ○ I prefer to evaluate it myself with otoscopy
- ○ I do not do anything if there are no complaints about it
- ○ I am not interested because it is not my area of expertise
- ○ Do not know

For peer review only

14. Do you think that your traumatic brain injury patients (where the trauma occurred more than 6 months ago) should use hearing aids if they have hearing loss?

*Required

- Yes, definitely
- If they would like, yes
- No need
- I think it is an issue that can be ignored as there are many impairments they are struggling with
- Do not know

15. In which situations can you help your adult patients with traumatic brain injury, who use hearing aids, during their appointment with you? You can choose multiple options

*Required

- I can change the battery of hearing aids
- I can notice whether or not it is working
- I can notice any feedback problem
- I can check the hearing aid ear mould and/or dome functionality
- I do not know anything about hearing aids

16. Did your patient group, who frequently report auditory and/or vestibular complaints after trauma, have a specific aetiology of traumatic brain injury? Can you generalize the trauma aetiology of these patients with auditory and/or vestibular complaints?

*Required

- Yes
- No
- Do not know

For peer review only

16.a. If you answered yes to question 16, what was this aetiology?

- Falls
- Vehicle-related collisions
- Violence
- Sport Injuries
- Other Trauma (Please describe)

16.a.i. If you selected Other, please specify:

For peer review only

Turkish Version of Survey

Sağlık Çalışanları ve Travmatik Beyin Hasarının İşitsel-vestibüler Sonuçları

Page 1: Bilgilendirme Formu

Bilgilendirme ve Onam Formu

Final version 1.2: 04.07.2022

Araştırma Başlığı: Travmatik beyin hasarı olan bireylerin odyo-vestibüler bulgularına ilişkinsağlık profesyonellerinin deneyimleri ve görüşleri

Araştırma Ekibi:

Danışmanlar:

Dr. Kathryn Fackrell, İşitme Bilimleri

Dr. Laura Edwards, Tıp ve Sağlık Bilimleri

Araştırmacı:

Uzm Ody. Kübra Bölükbaş, İşitme Bilimleri Doktora Öğrencisi

Tıp ve Sağlık Bilimleri Fakültesü Araştırma Etiği Ref. No: FMHS 462-0222

Bu çalışma, ***KBB ve odyoloji uzmanlarının dışındaki***, travmatik beyin hasarı olan erişkinlerleilgilenen sağlık profesyonellerinin görüş, deneyim ve yaklaşımlarını bu hastaların işitsel ve denge durumlarına odaklanarak incelemektedir.

İlgiliniz için teşekkür ederiz. ***KBB/odyoloji uzmanları dışında*** travmatik beyin hasarı olan yetişkin hastalarla ilgilenen bir sağlık çalışanı olduğunuz için çalışmamıza katılmaya davetlisiniz. Lütfen katılımcı olmadan önce bilgilendirme ve onam formunu dikkatlice okuyunuz.Herhangi bir sorunuz varsa aşağıda yer alan iletişim kanalları

For peer review only

aracılığıyla araştırmacılara ulaşmaktan lütfen çekinmeyin. Araştırmaya katılım gönüllülük esasına dayanmaktadır.

For peer review only

Ne yapmanızı isteyeceğiz?

Bilgilendirme sayfasının sonundaki "Next (İleri)" butonuna tıkladıktan sonra, bu çalışmaya katılabilmeniz için onam formunu kabul etmeniz istenecektir. Daha sonra, mesleğinizle ilgili demografik bilgiler (mesleğiniz, çalışma süreniz, kurum türü gibi) içeren birkaç soru ve travmatikbeyin hasarı olan yetişkin hastalarınızın işitsel-vestibüler bulgularıyla ilgili deneyimleriniz, görüşleriniz ve yaklaşımlarınızı öğrenmek amacıyla hazırlanan soruları cevaplamanız istenecektir. Tüm anket sorularını cevaplamanız en fazla yirmi (20) dakikanızı alacaktır. Tüm soruları mümkün olduğunca dürüst ve eksiksiz olarak cevaplamanızı rica ediyoruz.
Cevaplarınızı göndermeden önce, "Çıkış" butonuna tıklayarak ve/veya tarayıcınızı kaparatarak herhangi bir noktada, herhangi bir nedenle anketten çekilebilirsiniz. Ancak, son
sayfada bulunan "Bitir (Finish)" butonuna tıkladıktan sonra cevaplarınız tarafımıza gönderilecekve cevaplarınızı geri çekmeniz mümkün olmayacaktır.

Katılımcı olmanızın dezavantajları nelerdir?

Katılımcı olmanız için kıymetli zamanınızdan birkaç dakikanızı anketimize ayırmanız gerekecektir.

Katılımcı olmanızın avantajları nelerdir?

Diğer katılımcılar ve sizin katkınız sayesinde araştırmacılar, travmatik beyin hasarı olan bireylerin işitsel ve vestibüler durumları hakkında KBB ve odyoloji uzmanları haricindeki sağlık uzmanlarının görüşleri, yaklaşımları ve farkındalıkları hakkında bilgi edineceklerdir. Ayrıca, bu bireylerdeki işitsel-vestibüler sorunlara yaklaşımın uluslararası düzeyde nasıl olduğunun tespit edilmesine katkı sağlanacaktır. Böylece, çalışmanın sonuçlarından yola çıkılarak konuya ilişkingelecek araştırmaların da önü açılabilir.

Araştırmacılar çalışmaya katıldığınızı bilecekler mi?

İsim, soyisim ve kimlik bilgisi gibi kişisel verileriniz sorulmadığı için hiç kimse araştırmaya katıldığınızı bilmeyecektir. Ayrıca, cevaplarınız araştırma ekibine iletilirken kullanılan platformunfiltreleme sistemi sayesinde IP adresiniz gözükmeyecek ve saklanmayacaktır. Tüm çevrimiçi platformlarda ihlal riski mümkün olduğu gibi bu platformda da mümkündür. Ancak kişisel veri alınmaması ve şifreli bir sistemin kullanılması sayesinde bu risk minimuma düşürülmüştür.

Verilerinize ne olacak?

Anketin sonundaki "Bitir (Finish)" butonuna tıkladığınızda, anket sonuçlarınız şifre korumalı birveri tabanına kod numarası ile yüklenecektir. Araştırma ekibi anketin kim tarafından cevaplandığını göremeyecek ve bu nedenle, bu noktada, verileri geri çekmeniz

For peer review only

mümkün olmayacaktır. Araştırma verileriniz, Nottingham Üniversitesinin veri koruma politikasışartlarına göre sınırlı erişimli bir sunucuda şifre korumalı bir klasörde saklanacaktır. Veriler enaz 7 yıl saklandıktan sonra imha edilecektir.

Bu anket bir doktora projesi için hazırlanmıştır. Tüm katılımcılardan elde edilen yanıtlar analizedilmeye hazır şifre korumalı bir veritabanında birleştirilecektir. Sonuçlar doktora kapsamındabir tez olarak yazılmasının yanısıra akademik yayınlarda ve sunumlarda kullanılabilir. Bu çalışmadan elde edilen anonimleştirilmiş veriler, etik onayı ile gelecekteki araştırma ve öğrenimde kullanılmak üzere paylaşılabilir.

Daha fazla soru sormak için veya desteğe ihtiyaç duyduğunuzda bizimle iletişime geçerseniz, alacağımız tek kişisel veri e-postanız olacaktır. Ancak bu, tamamlamış olduğunuz anketinizdenayrı olarak ele alınacaktır ve verilerinizi birbirine bağlamak mümkün olmayacaktır. E-posta adresiniz yalnızca sorununuzu çözmek için gerektiği kadar saklanacak ve daha sonra imha edilecektir. Üniversitenin kişisel verileri nasıl işlediği hakkında daha fazla bilgi için lütfen şu adresi ziyaret ediniz: https://www.nottingham.ac.uk/utilities/privacy.aspx/

Verilerinize kim erişebilecek?

Nottingham Üniversitesi veri denetleyicisidir (veri güvenliğinden yasal olarak sorumludur) ve buçalışmanın danışmanları (yukarıda adı geçen) veri sorumlusudur (verilere erişimi yönetmektedirler) ve bu nedenle verilerinizin çalışmada nasıl kullanılacağını belirleyeceklerdir. Yanıtlarınız ve verileriniz yalnızca araştırma amacıyla kullanılacaktır. Araştırmamız, kamu yararını gözeterek gerçekleştirdiğimiz bir projedir.

Nottingham Üniversitesi'nin sorumlu üyelerine, çalışmanın doğru bir şekilde yürütüldüğündenemin olmak için izleme ve/veya denetleme için verilere erişim izni verilebilir.

Bu projeyle ilgili herhangi bir sorunuz veya endişeniz varsa, lütfen iletişime geçiniz:

Kübra Bölükbaş, E-mail: kubra.bolukbas@nottingham.ac.uk or

Bu çalışmanın herhangi bir yönü hakkında bir endişeniz varsa, lütfen araştırma danışmanları ileiletişime geçiniz:

Kathryn Fackrell,

Email: kathryn.fackrell@nottingham.ac.uk

Laura Edwards,

Email: laura.edwards@nottingham.ac.uk

For peer review only

Resmi olarak şikayette bulunmak isterseniz, FMHS Araştırma Etik Kurulu Yöneticisi ile iletişimegeçiniz:

FMHS-ResearchEthics@nottingham.ac.uk

For peer review only

Page 2: Onam Formu

Please select at least 5 answer(s). ✱

- [ ] Önceki sayfadaki bilgileri okuduğumu ve anladığımı onaylıyorum
- [ ] Odyolog ve/veya KBB uzmanı dışında, travmatik beyin hasarı olan yetişkinlerle ilgilenen bir sağlık çalışanıyım
- [ ] Katılımımın gönüllü olduğunu ve çalışmayı istediğim zaman sonlandırabileceğimi ve sayfayı kapatarak verilerimi geri çekebileceğimi anlıyorum
- [ ] Yanıtlarımın anonim olarak görüneceğini anlıyorum
- [ ] Bu çalışmadan elde edilen anonimleştirilmiş verilerin gelecekte etik onayı ile araştırma ve öğrenim amaçları için kullanılabileceğini anlıyor ve kabul ediyorum

Devam edebilmek için lütfen her bir kutucuğu onaylayın. Bilgilendirme ve onam formunukabul etmiyorsanız, lütfen tarayıcınızı kapatarak çıkış yapınız. ✱*Required*

For peer review only

Page 3: Travmatik Beyin Hasarı olan Bireylerin Odyo-vestibülerBulgularına İlişkin Sağlık Profesyonellerinin Deneyimleri ve Görüşleri

1. Mesleğiniz nedir? ✱ *Required*

- Nörolog
- Nöropsikiyatrist
- Beyin Cerrahı
- Genel Cerrahi Uzmanı
- Fiziksel Tıp ve Rehabilitasyon Uzmanı
- Geriatri Uzmanı
- Ortopedi ve Travmatoloji Uzmanı
- Hemşire
- Nöropsikolog
- Dil ve Konuşma Terapisti
- Ergoterapist
- Fizyoterapist
- Psikolog
- Diğer Sağlık Profesyoneli (Lütfen açıklayınız)

1.a. Diğer seçeneğini seçtiyseniz, lütfen açıklayın:

2. Kaç yıldır mesleğinizi yapıyorsunuz?✱ *Required*

- 5 yıldan az
- 5 ile 10 yıl arası
- 10 ile 20 yıl arası
- 20 yılı aşkın süredir

3. Travmatik beyin hasarı olan hastalarla kaç yıldır çalışıyorsunuz? * Required

- 5 yıldan az
- 5 ile 10 yıl arası
- 10 ile 20 yıl arası
- 20 yılı aşkın süredir

4. Hangi ülkede çalışıyorsunuz? * Required

- Almanya
- Amerika
- Avustralya
- Azerbaycan
- Belçika
- Birleşik Krallık
- Çin
- Danimarka
- Hindistan
- Hollanda
- İrlanda
- İspanya
- İsrail
- İsveç
- İsviçre
- İtalya
- Japonya
- Kanada
- Kıbrıs
- Polonya
- Suudi Arabistan
- Türkiye

- Yunanistan
- Diğer

4.a. Diğer seçeneğini seçtiyseniz, lütfen açıklayın:

5. İş yeriniz özel mi yoksa devlete mi ait?✱ *Required*

- Özel
- Devlet

6. Travmatik beyin hasarı olan yetişkin hastalarla haftada kaç saat çalışıyorsunuz? ✱ *Required*

- 1 saatten az
- 1 ile 5 saat arası
- 5 ile 10 saat arası
- 10 ile 20 saat arası
- 20 saatten fazla

7. Aşağıdaki şikayetler travmatik beyin hasarı geçirmiş kişiler tarafından bildirilebilir. Lütfen 6 aydan daha uzun süre önce kafa travmasına maruz kalmış hastalarınızın bunları size ne sıklıklatarif ettiğini belirtiniz. ✱ *Required*

Please don't select more than 1 answer(s) per row.

Please select at least 12 answer(s).

For peer review only

	Bütün hastaları mbunu bildiriyor	Hastalarımı nçoğu bunu bildiriyor	Bazı hastaları mbunu bildiriyor	Birkaç hastam bunu bildiriyor	Hiçbir hastam bunu bildirmiyor	Bilmiyorum
İşitme kaybı	☐	☐	☐	☐	☐	☐
Sessiz ortamda konuşmayı anlayamama	☐	☐	☐	☐	☐	☐
Gürültülü ortamda konuşmayı anlayamama	☐	☐	☐	☐	☐	☐
Sesin nereden geldiğini tespit edememe	☐	☐	☐	☐	☐	☐
Konuşmaları yakalayamama	☐	☐	☐	☐	☐	☐
Kulak çınlaması (Tinnitus)	☐	☐	☐	☐	☐	☐
Normal çevresel seslere karşı olağan dışı hassasiyet, tahammülsüzlük (Hiperakuzi)	☐	☐	☐	☐	☐	☐
Dengesizlik, Sersemlik (Dizziness)	☐	☐	☐	☐	☐	☐
Baş Dönmesi (Vertigo)	☐	☐	☐	☐	☐	☐
Fiziksel bir problemi olmaksızın karanlıkta yürüyememe	☐	☐	☐	☐	☐	☐

Farklı yüzeylerde yürürken dengesizlik (dizziness)	☐	☐	☐	☐	☐	☐
Harekete bağlı olarak uykuda veya uyanıkken baş dönmesi hissetmek	☐	☐	☐	☐	☐	☐

8. Aşağıdaki belirtiler travmatik beyin hasarı geçirmiş kişiler tarafından bildirilebilir ve/veyagözlemlenebilir. Lütfen 6 aydan daha uzun süre önce kafa travmasına maruz kalmış hastalarınızın bunları size ne sıklıkla bildirdiğini belirtiniz *✱Required*

Please don't select more than 1 answer(s) per row.

Please select at least 10 answer(s).

	Hastalarımın hepsi	Hastalarımın yarısından fazlası	Bazı hastalarım	Birkaç hastam	Hastalarımın hiçbiri bunu bildirmiyor	Bilmiyorum
Görüşmeleriniz sırasında konuşmanızı sıksık tekrarlamanızı ister	☐	☐	☐	☐	☐	☐
Konuşurken dudaklarınızı okur	☐	☐	☐	☐	☐	☐
Yüzünüz ona dönük olmadığında sizianlamaz	☐	☐	☐	☐	☐	☐
Telefonda sizi anlamakta zorluk çeker	☐	☐	☐	☐	☐	☐

For peer review only

Hasta yakınları hastanın çok yüksek sesle televizyon izlemeye başladığından şikayet eder	☐	☐	☐	☐	☐	☐
Ne hakkında konuştuğunuzu anlayamamaktan endişelendiği için randevularınıza gelmekten kaçınır	☐	☐	☐	☐	☐	☐
Sizinle görüşmesi sırasında çevresel/dış seslerden rahatsız olur	☐	☐	☐	☐	☐	☐
Belirli seslere tahammül edemediğini bildirir	☐	☐	☐	☐	☐	☐
Fiziksel ve zihinsel sorunları kısmen iyileşmiş olsa da, işitsel-vestibüler bozukluklar spor yapma, dans etme ve ev işi yapma gibi aktivitelerini etkiler	☐	☐	☐	☐	☐	☐

Denge bozuklukları nedeniyle refakatçi olmadan evden çıkmaya korkar	☐	☐	☐	☐	☐	☐

9. 6 aydan daha uzun süre önce meydana gelen travmatik beyin hasarına maruz kalmış hastalarınıza randevularınızda denge durumlarının nasıl olduğunu (sersemlik, başdönmesi vb.) soruyor musunuz? ✱*Required*

- ○ Evet, tüm hastalarıma sorarım
- ○ Denge durumları randevularımızı etkiliyorsa ve/veya işimi yapmamı engelliyorsa sorarım
- ○ Bu konu uzmanlık alanım olmadığı için sormamayı tercih ediyorum
- ○ Bu konuda herhangi bir şikayeti yoksa hiçbir şey sormam
- ○ Bilmiyorum

10. 6 aydan daha uzun süre önce travmatik beyin hasarına maruz kalan hastalarınızda baş dönmesi ve/veya denge bozukluğu fark ederseniz, aşağıdaki tarama yöntemlerinden hangisiniveya hangilerini kullanırsınız? Birden fazla yanıt seçebilirsiniz. ✱ *Required*

- ☐ Past Pointing testi
- ☐ Parmak Burun testi
- ☐ Unterberger testi (Fukuda adım
- ☐ testi)Romberg testi
- ☐ Gait analysis (Yürüme analizi)
- ☐ Göz hareketleri
- ☐ Head impulse testi
- ☐ Uzmanlık alanım olmadığı için değerlendirmeyi tercih etmiyorum
- ☐ Bilmiyorum
- ☐ Diğer

10.a. Diğer seçeneğini seçtiyseniz, lütfen açıklayın:

11. 6 aydan daha uzun süre önce travmatik beyin hasarına maruz kalmış hastanızı hangidurum veya durumlarda KBB ve/veya odyoloji departmanına sevk etmeyi düşünürsünüz? Birden fazla yanıt seçebilirsiniz.* Required

- Konuşmamı anlamadığını ve/veya duymadığını düşündüğümde hemen
- Dizziness ve/veya baş dönmesi gözlemlediğimde hemen
- Kulak çınlaması veya seslere karşı hassasiyet bildirirse hemen
- Durumunu fark etsem bile emin olmak için bir sonraki randevuyu beklerim
- Hasta herhangi bir şikayet bildirmedikçe sevk etmiyorum
- Bu hasta grubunun uğraştığı çok fazla problemi olduğu için görmezden gelinebilecek bir konu olduğunu düşünüyorum
- Bilmiyorum

review

12. 6 aydan daha uzun süre önce travmatik beyin hasarına maruz kalmış hastalarınızdantahmini olarak kaç tanesini odyoloji ve/veya KBB departmanına yönlendiriyorsunuz? *Required

- Hastalarımın hepsini
- Hastalarımın yarısından
- fazlasınıBazı hastalarımı
- Birkaç hastamı
- Hastalarımın hiçbirini yönlendirmiyorum
- Bilmiyorum

13. 6 aydan daha uzun süre önce travmatik beyin hasarına maruz kalmış hastalarınızın gerektiğinde, dış kulaklarının durumunu nasıl kontrol edersiniz? * Required

- KBB hekimine ve/veya odyoloji departmanına konsülte ederim

- Otoskop ile kendim değerlendirmeyi tercih ederim
- Bu konuda şikayeti yoksa hiçbir şey yapmam
- Uzmanlık alanım olmadığı için ilgilenmiyorum
- Bilmiyorum

14. 6 aydan daha önce travmatik beyin hasarına maruz kalan hastanızın işitme kaybı varsaişitme cihazı kullanması gerektiğini düşünüyor musunuz? * *Required*

- Evet, kesinlikle kullanmalı
- İsterse, evet
- Gerek yok
- Çok fazla problemle uğraştıkları için görmezden gelinebilecek bir konu olduğunu düşünüyorum
- Bilmiyorum

15. İşitme cihazı kullanan travmatik beyin hasarına maruz kalmış hastalarınıza randevularısırasında işitme cihazlarıyla ilgili hangi durumlarda yardımcı olabilirsiniz? Birden fazla yanıt seçebilirsiniz. * *Required*

- İşitme cihazlarının pilini değiştirebilirim
- Hastamın işitme cihazının çalışıp çalışmadığını anlayabilirim
- Herhangi bir feedback (ötme sesi) sorunu varsa fark edebilirim
- İşitme cihazının kulak kalıbını ve/veya dome (kubbe) işlevini kontrol edebilirim
- İşitme cihazları hakkında hiçbir şey bilmiyorum

16. Travma sonrası sıklıkla işitsel ve/veya vestibüler şikayetler bildiren hasta grubunuz,travmatik beyin hasarının spesifik bir etiyolojisine sahip miydi? İşitsel ve/veya vestibüler şikayetleri olan bu hastaların travma etiyolojisini genelleyebilir misiniz ? * *Required*

- Evet

- Hayır
- Bilmiyorum

16.a. 17. soruya evet cevabı verdiyseniz, bu etiyoloji neydi?

- Düşme
- Trafik kazası
- Şiddet
- Spor yaralanmaları
- Diğer nedenler(Lütfen açıklaynız)

16.a.i. Diğer seçeneğini seçtiyseniz, lütfen açıklayın:

For peer review only

Supplemental Appendix 2. List of organisations that were contacted to share the surveys

Name of Organisations	Country
Speech Pathology Australia	Australia
Rehabilitation Medicine Society of Australia and New Zealand	Australia and New Zealand
the Royal Australian and New Zealand College of Psychiatrist	Australia and New Zealand
Speech-Language & Audiology Canada	Canada
Hellenic Neuropsychological Society	Greece
Logopedista	Italy
Association of Occupational Therapists of Ireland	Ireland
Irish Association of Speech & Language Therapists	Ireland
Headway The Brain Injury Association	United Kingdom
The British Neuropsychological Society	United Kingdom
British Psychological Society	United Kingdom
The British Neuropsychiatry Association	United Kingdom
Northampton Speech Therapy (Independent Therapist)	United Kingdom
British Society of Rehabilitation Medicine	United Kingdom
Royal College of Speech and Language Therapists	United Kingdom
Association of Speech and Language Therapists	Türkiye
World Federation of Occupational Therapists	

For peer review only

The Clinical Experiences, Current Approaches, Opinions and Awareness of Healthcare Professionals regarding the Audio-Vestibular Consequences of Individuals with Traumatic Brain Injury: A Cross-sectional Online Survey Study

Journal:	*BMJ Open*
Manuscript ID	bmjopen-2023-078017.R1
Article Type:	Original research
Date Submitted by the Author:	20-Feb-2024
Complete List of Authors:	Bölükbaş, Kübra; University of Nottingham, Hearing sciences, Mental Health and Clinical Neuroscience, School of Medicine; NIHR Nottingham Biomedical Research Centre<br>Edwards, Laura; University Hospitals of Derby and Burton NHS Foundation Trust, Division of Rehabilitation Medicine; University of Nottingham, Injury, Inflammation and Recovery Sciences, School of Medicine<br>Baguley, David; University of Nottingham, Hearing sciences, Mental Health and Clinical Neuroscience, School of Medicine; University of Nottingham, NIHR Nottingham Biomedical Research Centre<br>Fackrell, Kathryn ; University of Nottingham, Hearing sciences, Mental Health and Clinical Neuroscience, School of Medicine; NIHR Nottingham Biomedical Research Centre
**Primary Subject Heading**:	Ear, nose and throat/otolaryngology
Secondary Subject Heading:	Health services research
Keywords:	TRAUMA MANAGEMENT, Audiology < OTOLARYNGOLOGY, Surveys and Questionnaires, Patient-Centered Care, Physicians, Brain Injuries

SCHOLARONE™
Manuscripts

I, the Submitting Author has the right to grant and does grant on behalf of all authors of the Work (as defined in the below author licence), an exclusive licence and/or a non-exclusive licence for contributions from authors who are: i) UK Crown employees; ii) where BMJ has agreed a CC-BY licence shall apply, and/or iii) in accordance with the terms applicable for US Federal Government officers or employees acting as part of their official duties; on a worldwide, perpetual, irrevocable, royalty-free basis to BMJ Publishing Group Ltd ("BMJ") its licensees and where the relevant Journal is co-owned by BMJ to the co-owners of the Journal, to publish the Work in this journal and any other BMJ products and to exploit all rights, as set out in our licence.

The Submitting Author accepts and understands that any supply made under these terms is made by BMJ to the Submitting Author unless you are acting as an employee on behalf of your employer or a postgraduate student of an affiliated institution which is paying any applicable article publishing charge ("APC") for Open Access articles. Where the Submitting Author wishes to make the Work available on an Open Access basis (and intends to pay the relevant APC), the terms of reuse of such Open Access shall be governed by a Creative Commons licence – details of these licences and which Creative Commons licence will apply to this Work are set out in our licence referred to above.

Other than as permitted in any relevant BMJ Author's Self Archiving Policies, I confirm this Work has not been accepted for publication elsewhere, is not being considered for publication elsewhere and does not duplicate material already published. I confirm all authors consent to publication of this Work and authorise the granting of this licence.

For peer review only

The Clinical Experiences, Current Approaches, Opinions and Awareness of Healthcare Professionals regarding the Audio-Vestibular Consequences of Individuals with Traumatic Brain Injury: A Cross-sectional Online Survey Study

Kübra Bölükbaş[1,2], Laura Edwards[3,4], David M Baguley[1,2], Kathryn Fackrell[1,2,5]

[1]Hearing Sciences, Division of Mental Health and Clinical Neuroscience, School of Medicine, University of Nottingham, UK

[2]National Institute of Health and Social Research (NIHR) Nottingham Biomedical Research Centre, Nottingham, UK

[3]Division of Rehabilitation Medicine, University Hospitals of Derby and Burton NHS Foundation Trust, UK

[4]Injury, Inflammation and Recovery Sciences, School of Medicine, University of Nottingham, UK

[5]School of Healthcare Enterprise and Innovation, University of Southampton, UK

Corresponding author: Kübra Bölükbaş, NIHR Nottingham Biomedical Research Centre, Ropewalk House, 113 The Ropewalk, Nottingham, NG1 5DU, UK.

Email: kubra.bolukbas@nottingham.ac.uk

For peer review only - http://bmjopen.bmj.com/site/about/guidelines.xhtml

ABSTRACT

Objective: To explore the experiences, current approaches, opinions, and awareness of healthcare professionals (HCPs) caring for adults with Traumatic Brain Injury (TBI) regarding the audio-vestibular consequences.

Design/setting: Cross-sectional online survey study

Participants: HCPs with experience of caring for adults with TBI, who were not ENT specialists or audiologists.

Methods: The study was conducted from May 2022 to December 2022. The online survey consisted of 16 closed and open-text questions in English and Turkish about clinical experience, current approaches and awareness of audio-vestibular consequences following TBI. Frequencies of responses to closed questions and associations between variables were analysed using SPSS-v28. Open-text responses were summarised in Microsoft Excel.

Results: Seventy HCPs participated from 17 professions and 14 countries, with the majority from the UK (42.9%). HCPs stated that 'some' to 'all' of their patients had auditory problems such as 'inability to understand speech-in-noise' (66%), 'tinnitus' (64%), 'hyperacusis' (57%), and balance problems such as 'dizziness' (79%) and 'vertigo' (67%). Usually, HCPs asked about the balance status of patients at appointments and when they observed dizziness and/or balance disorder they used screening tests, most commonly Finger-to-Nose (53%). For auditory impairments, HCPs preferred referring TBI patients to Audiology/ENT services. However, 6% of HCPs felt that audio-vestibular conditions could be ignored upon referral because TBI patients struggled with many impairments. Additionally, 44% would suggest hearing aids to TBI patients with hearing loss 'if they would like to use' rather than 'definitely'.

Conclusions: Many audio-vestibular impairments are observed by HCPs caring for TBI patients. The assessment and intervention opinions and awareness of HCPs for these impairments vary. However, non-expert HCPs may not be aware of negative consequences of untreated audio-vestibular impairments following TBI. Therefore, developing a simple framework for screening and indications of audio-vestibular impairments for referral may be helpful for non-audiological specialists regularly seeing these patients.

Keywords: Traumatic Brain Injury, Healthcare Professionals, Auditory, Vestibular, Balance, Survey

For peer review only

STRENGTHS AND WEAKNESSES OF THIS STUDY

1. To the best of our knowledge, this is the first study conducted with HCPs other than ENT specialists and audiologists on the audio-vestibular outcomes of patients with TBI, utilizing an international design.
2. The multidisciplinary approach to recruitment using established academic networks resulted in a range of respondents from different health disciplines.
3. The creation of two versions of the survey enhanced inclusivity and made it possible for respondents especially from Türkiye to participate who may not be proficient in English.
4. Despite our efforts to engage HCPs from across the world the majority of respondents were from the UK and as such findings may reflect the UK health system.

For peer review only

For peer review only - http://bmjopen.bmj.com/site/about/guidelines.xhtml

INTRODUCTION

Traumatic brain injury (TBI) is defined as a structural injury and/or physiological deterioration of brain function following trauma due to an external force [1]. It is estimated that 64-74 million people every year experience a TBI [2] and it is one of the leading causes of death and disability worldwide [3,4]. There are many different complications associated with TBI, such as cognitive, emotional, behavioural, and audio-vestibular impairments [5–8]. In addition, neck pain, balance disorders and tinnitus (ringing in the ear(s)) have been reported as some of the most common complaints after head and neck trauma [9,10]. Moreover, in a review investigating hearing loss in TBI without skull fracture, Chen et al. [11] found that 58% of TBI patients experienced hearing loss, sometimes temporarily, despite not having any skull fractures. This rate suggests that the percentage of audio-vestibular consequences related to TBI that are not reported and/or investigated worldwide may be much higher than expected.

Consequences of TBI vary greatly by nature, severity and duration. Some individuals may be left with minimal impairment and make a rapid recovery with/without specialist intervention, whilst others may require inpatient or outpatient neurorehabilitation [1]. This diversity of complications due to TBI necessitates a multidisciplinary approach. Accordingly, depending on the severity and nature of the complication, a patient with TBI may be treated by a wide range of healthcare professionals (HCPs) including physiotherapists, psychologists, occupational therapists. The emergence of hearing loss and/or vestibular disorders, tinnitus, or hyperacusis (sound sensitivity) following TBI further complicates the treatment of TBI and requires a sensitive and careful approach in the diagnosis and treatment of audio-vestibular disorders in this patient group. Untreated hearing loss for example can lead to additional conditions such as dementia [12] as well as many psychological problems such as communication difficulties and social isolation [13,14]. Therefore, assessment and treatment of audio-vestibular conditions should not be neglected when caring for adults with TBI as early detection of audio-vestibular impairments in patients after TBI can improve quality-of-life, minimise complications and prevent additional conditions such as dementia.

Patients struggling with many problems such as physical, cognitive, emotional and behavioural complications after TBI may not easily notice any audio-vestibular impairments immediately. However, HCPs who care for them should understand and assess their audio-vestibular conditions, even if it is not their field of expertise and, if necessary, arrange referrals to the ENT (ear nose throat) specialists or audiologists. A recent survey on audio-vestibular clinical

For peer review only - http://bmjopen.bmj.com/site/about/guidelines.xhtml

practice within Australian audiology departments found gaps in audiologist knowledge and inadequate resources and practice regarding the identification, diagnosis and management of patients with auditory and/or vestibular deficits following TBI [15]. However, as yet, the clinical experiences, current approaches, opinions and awareness of HCPs who specialise in managing patients with TBI have not been fully investigated. It is therefore important to understand the experiences of these HCPs in particular to understand their awareness and knowledge of the audio-vestibular consequences of TBIs, in order to understand and identify areas that could be improved in existing practices.

Accordingly, the aim of this study was to explore and gain an understanding of the clinical experiences, current approaches, opinions, and awareness of the HCPs caring for adults with TBI (except ENT specialists and audiologists) regarding the audio-vestibular consequences experienced by these patients.

METHODS

Design

This study used a cross-sectional international online survey to explore the clinical experiences, current approaches, opinions and awareness of HCPs regarding the audio-vestibular conditions experienced by patients who had a TBI. Research approval was obtained from the University of Nottingham, Faculty of Medicine and Health Sciences Research Ethics Committee (FMHS 462-0222, May 2022). Before initiating the survey questions, on the first page, an information page was provided detailing who the researchers are, the purpose of the survey, its duration, the advantages and disadvantages of participation, who would have access to the survey data, where and for how long the data would be stored. Following the information page, all participants were requested to give informed consent. This survey study is reported according to Checklist for Reporting Results of Internet E-Surveys (CHERRIES) [16].

Survey Development

The survey questions were iteratively developed and refined through discussion and consensus within the study team. The authors initially compiled a list of potential questions and relevant sections for inclusion in the survey. Questions were based on experience of the authors and evidence from research. To ensure the questions and response options were relevant and valid for HCPs, the surveys were piloted with two HCPs and one researcher who have experience of

For peer review only - http://bmjopen.bmj.com/site/about/guidelines.xhtml

audio-vestibular assessments. The questions were revised and distilled down to create a 16-question survey (over 10 pages, with a maximum of 3 questions on per page) which included closed questions with single-choice, multiple-choice options, or Likert scales and for 4 questions with a free-text “other” option. Sub-question (16a) of the last question used adaptive questioning, which was only displayed conditionally based on ‘yes' responses to question 16. Respondents were asked to consider patients with TBIs that occurred more than 6 months ago and the reported or observed audio-vestibular symptoms at that time. It was necessary to answer each question and to complete the survey in one session. It was possible for respondents to review and edit their responses until the point they chose to submit the survey on the final page. In order to reach a range of HCPs, and since the lead author (KB) is bilingual, two versions of the survey were created: one in English and one in Turkish (Supplemental appendix 1). The survey was delivered online using JISC software maintained by the University of Nottingham (https://www.onlinesurveys.ac.uk/) which collected and stored responses from each respondent. Respondents accessed the open survey via a link (passwords were not provided).

Patient and Public Involvement

No patients were directly involved in the development, dissemination or reporting of this study. The study focused on the experiences of HCPs in the field of TBI, such as physiotherapists, neuropsychologists, rehabilitation nurses, therefore the survey was piloted with HCPs and a researcher who have experience of audio-vestibular assessments to gain their views and refine questions, and two of the authors are clinicians with relevant experience in the field.

Study Sample and Eligibility

The study population was identified as HCPs caring for adult patients with TBI worldwide. To be eligible to participate in the survey, respondents were required to be HCPs with clinical experience in caring for adult patients with TBI and to have the ability to understand and answer questions in English (except HCPs in Türkiye). ENT specialists and audiologists were excluded from the study as they were already experts in the field of audio-vestibular care.

Distribution

A number of recruitment strategies were used to ensure a broad, representative sample of HCPs with a range of experiences, current approaches, opinions and awareness regarding audio-vestibular conditions in TBI patients. The survey invitation and link were circulated via

For peer review only - http://bmjopen.bmj.com/site/about/guidelines.xhtml

professional organisations' contact lists including Headway UK, British Society of Physical and Rehabilitation Medicine, and Royal College of Speech and Language Therapists (see Supplemental appendix 2 for full list), promotion in blogs (e.g., Krysalis Neuro Occupational Therapy), and through social media channels (Facebook, Twitter, and Instagram). In addition, an invitation email with the survey link was sent to the HCPs identified via Expertscape website (https://expertscape.com/) as experts in the field of TBI. All of these distribution channels were selected taking into account the inclusion criteria of our study. Participants were not given a specific timeframe to complete the survey. Responses were obtained between May 2022 and December 2022. Participation in the survey was voluntarily and no incentives were provided.

Data analysis

All survey data were imported to Microsoft Excel 2016©. Prior to analysis, data were checked to ensure all respondents fully consented and responses were given to all questions as required. If any surveys were terminated early, they were not visible because they were not submitted. Only completed surveys were analysed. Descriptive statistics, in particular frequencies and percentages of responses to closed questions were calculated in SPSS (v28). Open-text responses (short sentences or lists) were reviewed and categorized in Microsoft Excel 2016©. In particular, similar responses were categorized together and then summarised.

Occupations, countries of residence, years of occupation, years of working with TBI patients, and hours per week working with TBI adults were analysed using non-parametric statistical methods to assess relations between these categorical variables. To ensure a meaningful statistical analysis in comparisons, countries of residence were divided into four groups (the UK, Europe, Asia and Others) and occupations into six groups as seen in Table 1 (Group 1 representing physiotherapists; Group 2 occupational therapists; Group 3 speech and language therapists; Group 4 physiatrist or rehabilitation medicine doctors and rehabilitation nurses; Group 5 doctors from other specialties, and Group 6 'other') due to the imbalance in participant numbers. The Kruskal-Wallis test was utilized for comparing more than two independent groups in each category. Following this measurement, to identify the groups that contribute to the observed differences in the data, pairwise subgroup comparisons were conducted using Mann-Whitney U test with Bonferroni's correction for country of residence groups: $p<0.05/(4*3/2)$, for occupation groups: $p<0.05/(6*5/2)$, for years working with TBI adults groups: $p<0.05/(4*3/2)$.

For peer review only - http://bmjopen.bmj.com/site/about/guidelines.xhtml

RESULTS

Characteristics of participants

The characteristics of respondents are reported in Table 1. A total of 70 HCPs completed the survey from 14 different countries. Of these, the majority were from the UK (n=30, 42.9%), followed by Türkiye (n=14, 20%) and New Zealand (n=8, 11.5%). Respondents reported a range of occupations, including physiotherapists (n=15, 21.4%), occupational therapists (n=11, 15.8%) and speech and language therapists (n=10, 14.2%). The majority (48/70, 69%) had been in their occupation for more than 10 years and over half (39/70, 56%) had been working with TBI patients between 5 and 20 years. Over half of the respondents (57%) spent more than 10 hours per week working with adult patients with TBI. There were no statistically significant differences in years of occupation and years working with TBI adults among participants based on groups of country of residence and occupations ($p>0.05$). However, statistically significant differences were observed in the working hours per week with TBI adults based on groups of country of residence and occupations in the Kruskal-Wallis test ($p<0.05$). Subgroup statistical analyses showed that participants from the UK (n=16, 23%) were more likely to spend more than 20 hours a week with TBI adults, but those from Asia (n=7, 10%) were more likely to spend less than 1 hour per week ($p<0.008$). Participants in Group 2 (occupational therapists; n=6, 9%) were reported to spend over 20 hours per week with TBI adults, whilst participants in Group 5 (doctors from other specialties; n=5, 7%) spent less than 1 hour per week with TBI adults ($p<0.003$). Exactly half of the respondents worked in private healthcare (35/70, 50%), and the other half worked in National Health Service (NHS) or state-owned hospitals.

Table 1. Demographic information of participants

Characteristics	n	%	Statistical analysis groups
	**70**	**100**	
**Country of residence**			
United Kingdom	30	42.9%	UK
Türkiye	14	20.0%	Asia
New Zealand	8	11.5%	Others
Ireland	6	8.6%	Europe
Canada	2	2.9%	Others
Australia	2	2.9%	Others
Greece	1	1.4%	Europe

For peer review only

United States of America	1	1.4%	Others
Azerbaijan	1	1.4%	Asia
Italy	1	1.4%	Europe
Spain	1	1.4%	Europe
Croatia	1	1.4%	Europe
Venezuela	1	1.4%	Others
Tanzania	1	1.4%	Others
**Occupation**			
Physiotherapist	15	21.4%	Group 1
Occupational Therapist	11	15.8%	Group 2
Speech and Language Therapist	10	14.2%	Group 3
Physiatrist or Rehabilitation Medicine Doctor	6	8.6%	Group 4
Neuropsychiatrist	5	7.1%	Group 5
Neuropsychologist	4	5.8%	Group 6
Rehabilitation Nurse	4	5.8%	Group 4
Neurologist	3	4.2%	Group 5
Psychologist	2	2.9%	Group 6
Geriatrician	2	2.9%	Group 5
Neurosurgeon	2	2.9%	Group 5
Optometrist	1	1.4%	Group 6
Case Manager	1	1.4%	Group 6
Anaesthetist	1	1.4%	Group 5
Behavioural Scientist	1	1.4%	Group 6
Primary Care Doctor	1	1.4%	Group 5
Orthopaedic Surgeon	1	1.4%	Group 5
**Years in occupation**			
Less than 5 years	8	11%	Less than 5 years
5 – 10 years	14	20%	5 – 10 years
10 – 20 years	25	36%	10 – 20 years
More than 20 years	23	33%	More than 20 years
**Years working with TBI adults**			
Less than 5 years	16	23%	Less than 5 years
5 – 10 years	20	29%	5 – 10 years
10 – 20 years	19	27%	10 – 20 years
More than 20 years	15	21%	More than 20 years
**Hours per week working with TBI adults**			
Less than an hour	7	10%	Less than an hour

For peer review only - http://bmjopen.bmj.com/site/about/guidelines.xhtml

1 – 5 hours	13	19%	1 – 5 hours
5 – 10 hours	10	14%	5 – 10 hours
10 – 20 hours	15	21%	10 – 20 hours
More than 20 hours	25	36%	More than 20 hours

The clinical experiences of HCPs regarding audio-vestibular consequences in TBI patients

This section explores the reported and/or observed audio-vestibular symptoms in adult patients with TBI. According to the results, the majority of responses ranged across ‘a few’ to ‘most of my patients’ describe the symptom (Figure 1). In general, HCPs reported that ‘some’ to ‘all’ of their patients had auditory problems such as ‘inability to understand speech-in-noise’ (46/70, 66%), ‘tinnitus’ (45/70, 64%), ‘hyperacusis’ (40/70, 57%), and ‘inability to capture conversations’ (44/70, 63%) and balance problems such as ‘dizziness’ (55/70, 79%), and ‘vertigo’ (47/70, 67%). However, the majority of HCPs observed that ‘none’ to ‘a few’ patients reported problems with ‘inability to walk in the dark without a physical problem’ (31/70, 44%), ‘inability to understand speech in quiet’ (40/70, 57%) and ‘hearing loss’ (41/70, 58%). According to the Kruskal-Wallis test, no significant differences were found in the frequency of audio-vestibular symptoms reported by patients to HCPs, based on years of working with TBI adults and groups of country of residence ($p>0.05$). Statistically significant differences were observed for symptom of ‘inability to capture conversations’ based on groups of occupations ($p<0.05$). According to the Mann-Whitney U test with Bonferroni correction, a statistically significant difference was detected between Group 3 (speech and language therapists) and Group 1 (physiotherapists) for the symptom of ‘inability to capture conversations’ ($p<0.003$). Most speech and language therapists (n=7, 10%) reported that ‘most’ of their patients described ‘inability to capture conversations’, whilst most physiotherapists (n=10, 14%) reported that only ‘a few’ of their patients reported this symptom.

*** Insert Figure 1 here ***

The observed audio-vestibular symptoms by HCPs in patients with TBI (where trauma occurred more than 6 months ago) or reported to HCPs by patients’ relatives are shown in Figure 2. HCPs observed that ‘some’ to ‘all’ of their patients ‘asked to repeat their speech frequently during their interviews’ (45/70, 64%), ‘difficulty understanding them on the phone’

For peer review only

(38/70, 54%), and 'an inability to tolerate certain sounds' (44/70, 63%). In contrast, 37% of HCPs (26/70) did state that 'none' of their patients 'avoided coming to appointments because they were concerned, they would not understand what HCPs were talking about'. However, 17% (12/70) did not know whether there was such a concern for their patients. The details of the statistical analysis results regarding the associations between audio-vestibular symptoms observed by HCPs or reported by patients' relatives to HCPs, and the years working with TBI adults and groups of country of residence, are explained in Supplemental Appendix 3.

Statistically significant differences were observed in the Kruskal-Wallis test between groups of occupations regarding the symptoms of 'asking to repeat their speech frequently during their interviews', 'not understanding HCPs when they are not facing with them', 'difficulty understanding HCPs on the phone' ($p<0.05$). Subgroup statistical analyses demonstrated significant differences in communication-related symptoms between Group 1 (physiotherapists) and Group 3 (speech and language therapists) ($p<0.003$). Based on this, most speech and language therapists (n=7, 10%) reported that 'more than half' of their patients asked to repeat their speech frequently during interviews, whilst the majority of physiotherapists (n=12, 17%) indicated that 'some' to 'a few' of their patients asked to repeat their speech frequently. Additionally, most speech and language therapists (n=6, 9%) stated that 'some' of their patients did not understand them when they were not facing them, similar to physiotherapists where the same percentage (n=6, 9%) reported observing this problem in 'a few' of their patients. The majority of speech and language therapists (n=6, 9%) reported that 'some' of their patients had difficulty understanding them on the phone, whilst physiotherapists (n=4, 6% for each option) had an equal percentage distribution among the options 'some', 'a few' of their patients, and 'do not know'. The statistical differences in other symptoms observed among other groups of occupations are detailed in Supplemental Appendix 3.

Over 40% (30/70) of HCPs were unable to identify and generalise the aetiology of the TBI patients who frequently reported audio-vestibular complaints. Of the 21% (15/70) that were able, most reported that likely aetiologies were vehicle-related collisions (9/15, 60%), followed by falls (2/15, 13%) and sports injuries (1/15, 7%). Those who selected 'other' trauma option (3/15, 20%) indicated that audio-vestibular complaints could result from any TBI aetiology, including an object falling on the head, vehicle related collisions and/or sports related injuries.

*** Insert Figure 2 here ***

For peer review only - http://bmjopen.bmj.com/site/about/guidelines.xhtml

The current approaches to assess and manage audio-vestibular conditions of TBI patients

To understand the current approaches used by HCPs regarding the audio-vestibular conditions of their TBI patients, we asked questions about screening and assessment methods. Over half of respondents (52/70, 74%) indicated that they ask their TBI patients about their balance status (e.g., dizziness, vertigo), whilst the remaining stated that they only asked if their balance status was interfering with the conduct of the appointment (10/70, 14%), they did not ask if there are no complaints (5/70, 7%), or they preferred not to ask because it was not their expertise (1/70, 1%). When they noticed dizziness or balance disorder in TBI patients, the most commonly used screening methods were finger-to-nose (37/70, 53%), gait analysis (34/70, 49%), and eye movements (34/70, 49%) as seen in Figure 3. In addition to these tests, HCPs also reported using evaluation methods such as videonystagmography (VNG) and dynamic visual acuity in open-text responses. Again, several HCPs reported that they prefer not to use any screening method because it was not their area of expertise (23/70, 33%). In relation to checking the external ear condition of TBI patients, 13% (9/70) of HCPs, in particular physiatrists or rehabilitation medicine doctors reported that they evaluated 'the external ear' by themself with an otoscope. However, almost half of the respondents (34/70, 49%) stated that they would refer patients to the ENT or audiology department to check the external ear conditions.

*** Insert Figure 3 here ***

When asked how many TBI patients they overall referred to ENT and/or audiology, 78% (55/70) of HCPs said that they referred 'some' or 'a few' of their TBI patients. Only 4% (3/70) of HCPs referred 'all' of their TBI patients to ENT and/or audiology department. In the questions asked to understand the opinions and awareness of HCPs, HCPs stated that they considered referring TBI patients to the ENT specialist and/or audiology department, when their patients could not understand and/or hear their speech (37/70, 53%), when their patients reported tinnitus and hyperacusis (33/70, 47%) or when they observed vertigo and/or dizziness in their patients (29/70, 41%). Only 6% (4/70) felt that audio-vestibular conditions could be ignored because TBI patients struggled with many impairments (Figure 4). In contrast, when asked whether TBI patients should use a hearing aid if they have hearing loss, no one thought it was something that could be ignored. However, most of the respondents (31/70, 44%) stated that TBI patients with hearing loss should use hearing aids 'if they would like to', rather than 'definitely', whilst the remaining did not know (14/70, 20%). Among the participants who indicated not knowing the majority were physiotherapists (n=8, 11%). When asked if they could help their TBI patients using hearing aids at their appointments, interestingly whilst 40%

For peer review only - http://bmjopen.bmj.com/site/about/guidelines.xhtml

(28/70) stated that they did not know anything about hearing aids, 40% (28/70) also stated that they could change the battery of the hearing aid. The majority (32/70, 46%), however, did state that they would notice whether the hearing aid was working. Notably, speech and language therapists were not among the HCPs who reported not knowing anything about hearing aids.

*** Insert Figure 4 here ***

DISCUSSION

This study explored the clinical experiences, current approaches, opinions, and awareness of HCPs of audio-vestibular consequences in TBI patients. In general, the majority of HCPs had observed audio-vestibular impairments in their TBI patients in line with the literature [17–19]. However, most HCPs reported that hearing loss was only observed in 'a few' of patients. In contrast to this finding, a systematic review study which investigated peripheral auditory dysfunction secondary to TBI showed that sensorineural hearing loss was the most frequently observed auditory impairment at a rate of 37.3% in patients with non-blast related TBI [17]. Additionally, Knoll et al. [20] showed that following TBI, individuals can experience auditory symptoms even if their hearing thresholds are within the normal range (≤25 dB). Therefore, these studies suggest that hearing loss can be observed or reported more frequently by TBI patients than HCPs may observe. One potential explanation for these differences could be that the HCPs who participated in this study are not experts in audio-vestibular assessments and as such may not be looking for audiological impairments, particularly during appointments which may be addressing other concerns. However, recognition of hearing loss in TBI patients by non-audiological-specialist HCPs may result in more onwards referrals for detailed assessment and input, resulting in better outcomes.

We also observed that HCPs were more likely to assess their TBI patients' vestibular and/or balance-related conditions than their hearing-related conditions. One explanation for this is that different HCPs such as neurologists, physiotherapists and occupational therapists, who participated in this study, may be more likely to assess and treat balance and/or vestibular function, and less likely to be familiar with auditory assessments. Another explanation could be that these professional groups prioritize physical function. These factors might be a possible reason why patients with auditory problems were more likely to be referred. Additionally, the observation of communication-related symptoms more likely in individuals with TBI by speech and language therapists rather than physiotherapists can be associated with their field

For peer review only - http://bmjopen.bmj.com/site/about/guidelines.xhtml

of expertise in communication. These findings may also contribute to emphasizing the importance of differences in patient care among professional groups and a multidisciplinary approach.

However, in contrast, a number of HCPs did not refer to ENT or audiology services if the patient had not complained about auditory problems and/or if they felt that the audio-vestibular problems were an issue that were not a priority compared to other TBI-related impairments. This suggests that some HCPs may not be aware of the negative short-term and long-term consequences of untreated audio-vestibular impairments. Many HCPs reported that they would leave the use of a hearing aid to the discretion of individual patients. This is understandable, in that patients who do not want to use a hearing aid are unlikely to use one, even if it is provided. However, it may also reflect lack of training and education in hearing aids for HCPs. It is important that HCPs explore the patient's rationale and capacity [21] for making this decision as well as explaining the potential consequences of untreated hearing loss [12].

Finally, consistent with the studies in the literature [15,22], several HCPs reported that TBI patients with audio-vestibular complaints had the most vehicle-related collision aetiology. However, this was not universal and likely reflects the fact that audio-vestibular complaints are a common consequence of a wide variety of TBI aetiologies.

Interestingly, a recent Australian study [15] also showed that audiologists lacked knowledge about the assessment and management of audio-vestibular impairments of TBI patients in audiological services. Whilst there can be variation in understanding of audio-vestibular consequences of TBI across clinical services and/or centres around the world, this study demonstrated a similar lack of awareness regarding the audio-vestibular consequences of TBI patients in non-audiology HCPs. This highlights the evident need for greater awareness among HCPs working with this patient population, even if it is outside their area of expertise. For this purpose, it may be beneficial to prepare a practical audiology guide for all HCPs dealing with TBI in future studies.

Strengths and Limitations

To our knowledge, this is the first study to investigate the clinical experiences, current approaches, opinions and awareness of HCPs, other than ENT specialists and audiologists, regarding the audio-vestibular consequences of TBI patients. This international survey received responses from a total of 70 participants from 17 different departments and 14 countries and as such we have gained knowledge from a range of HCPs who are actively

For peer review only - http://bmjopen.bmj.com/site/about/guidelines.xhtml

working in clinical settings that assess and manage audio-vestibular consequences in TBI patients. However, we do recognise that the majority were from the UK and that there are low to no participation rates for some countries. This means that some of the findings may reflect the UK health system above others. For example, other countries may have audiologists/ENTs as part of their multi-disciplinary team. Open online surveys may have inherent issues such as the possibility of participants submitting multiple responses and leading to response fraud in recruitment through particularly social media. However, anonymity with this method allows respondents to provide honest and candid answers freely [23]. Although no tracking method was used to address these issues in our study, the shared social media accounts and links included individuals from the academic community and HCPs. In addition, responses were checked for inconsistencies or evidence of deception through data cleaning. Furthermore, the inclusion of HCPs such as physiotherapists working in the field of balance may have influenced the results, in particular the confidence in vestibular assessments. Finally, we wanted to understand the experience of HCPs who specialise in TBI therefore we did not include audiologists and/or ENT specialists in this survey and as such we do not know their experiences with this patient population, except for the findings from the Australia survey [15] which indicate similar experiences to our findings here. Future research should consider further investigation into the experiences of audiologists/ENT specialists in relation to this patient population, in particular the assessments that should be carried out.

Conclusion

HCPs who care for individuals who sustained TBI observe a wide range of audio-vestibular outcomes in these patients. HCPs' opinions and awareness around further assessment, investigation and treatment were variable in this study. Untreated audio-vestibular impairments may have significant adverse consequences for patients and non-expert clinicians may be unaware of these. We suggest that standardised screening protocols may enable easier identification of audio-vestibular problems with signposting to appropriate specialties for further assessment and intervention. Thus, audio-vestibular impairments of TBI patients can be diagnosed and treated earlier, to improve quality-of-life of TBI patients.

For peer review only - http://bmjopen.bmj.com/site/about/guidelines.xhtml

ACKNOWLEDGEMENTS

The authors wish to acknowledge the clinicians who piloted the survey and all the respondents of the survey. We would like to express our gratitude to Professor David Baguley, who sadly passed away during this study. His knowledge and expertise helped develop the fundamental ideas for this research, for which we are very thankful.

AUTHOR STATEMENT

DB, LE, KB authors conceptualised the project and design of the survey questions. KB led the recruitment and data collection, supported by KF, DB, LE. KF, DB and LE informed methodology and interpretation of analysis. KB conducted initial analysis. KF and LE reviewed and refined analysis. KB drafted manuscript. KF, LE and KB reviewed and approved the final manuscript for submission.

FUNDING

This work was supported by the Ministry of National Education of the Republic of Türkiye and the National Institute for Health Research (NIHR) Nottingham Biomedical Research Centre.

DISCLAIMER

KB was funded by the Ministry of National Education of the Republic of Türkiye to undertake this work as part of her PhD. KF was funded by National Institute for Health Research (NIHR Post-Doctoral Fellowship, PDF-2018-11-ST2-003) at the time of completing this work. The views expressed in this publication are those of the author (s) and not necessarily those of the NIHR, the NHS or the Department of Health and Social Care.

COMPETING INTERESTS

None declared

PATIENT CONSENT FOR PUBLICATION

Not required

For peer review only

For peer review only - http://bmjopen.bmj.com/site/about/guidelines.xhtml

ETHICS APPROVAL

Research approval was obtained from the University of Nottingham, Faculty of Medicine and Health Sciences Research Ethics Committee (FMHS 462-0222, May 2022). All respondents gave informed consent at the beginning of the survey.

PROVENANCE AND PEER REVIEW

Not commissioned; externally peer reviewed.

DATA SHARING STATEMENT

Data are available upon reasonable request.

ORCID IDS

Kübra Bölükbaş https://orcid.org/0000-0002-7625-4758

Laura Edwards https://orcid.org/0000-0003-1392-2648

Kathryn Fackrell http://orcid.org/0000-0001-6529-8643

TWITTER

Kubra Bolukbas @_kubrabolukbas_, Kathryn Fackrell @FackrellKathryn

For peer review only

For peer review only - http://bmjopen.bmj.com/site/about/guidelines.xhtml

REFERENCES

1. NICE. Head injury briefing paper. 2014 [cited 2023 Jan 2];77. Available from: https://www.nice.org.uk/guidance/qs74/documents/head-injurybriefing- paper2.
2. Dewan MC, Rattani A, Gupta S, Baticulon RE, Hung YC, Punchak M, et al. Estimating the global incidence of traumatic brain injury. J Neurosurg. 2019 Apr 1;130(4):1080–97.
3. Maas AIR, Menon DK, Steyerberg EW, Citerio G, Lecky F, Manley GT, et al. Collaborative European NeuroTrauma Effectiveness Research in Traumatic Brain Injury (CENTER-TBI): A Prospective Longitudinal Observational Study. Neurosurgery [Internet]. 2015;76(1). Available from: https://journals.lww.com/neurosurgery/Fulltext/2015/01000/Collaborative_European_NeuroTrauma_Effectiveness.8.aspx
4. Rubiano AM, Carney N, Chesnut R, Puyana JC. Global neurotrauma research challenges and opportunities. Vol. 527, Nature. Nature Publishing Group; 2015. p. S193–7.
5. Ahmed S, Venigalla H, Mekala HM, Dar S, Hassan M, Ayub S. Traumatic brain injury and neuropsychiatric complications. Vol. 39, Indian Journal of Psychological Medicine. Medknow Publications; 2017. p. 114–21.
6. Konrad C, Geburek AJ, Rist F, Blumenroth H, Fischer B, Husstedt I, et al. Long-term cognitive and emotional consequences of mild traumatic brain injury. Psychol Med. 2011 Jun;41(6):1197–211.
7. Hoover EC, Souza PE, Gallun FJ. Auditory and cognitive factors associated with speech-in-noise complaints following mild traumatic brain injury. In: Journal of the American Academy of Audiology. American Academy of Audiology; 2017. p. 325–39.
8. Campbell KR, Parrington L, Peterka RJ, Martini DN, Hullar TE, Horak FB, et al. Exploring persistent complaints of imbalance after mTBI: Oculomotor, peripheral vestibular and central sensory integration function. Journal of Vestibular Research. 2021;31:519–30.

For peer review only - http://bmjopen.bmj.com/site/about/guidelines.xhtml

9. Kreuzer PM, Landgrebe M, Schecklmann M, Staudinger S, Langguth B. Trauma-Associated Tinnitus: Audiological, Demographic and Clinical Characteristics. PLoS One [Internet]. 2012 Sep 26;7(9):e45599-. Available from: https://doi.org/10.1371/journal.pone.0045599

10. Elzière M, Devèze A, Bartoli C, Levy G. Post-traumatic balance disorder. Vol. 134, European Annals of Otorhinolaryngology, Head and Neck Diseases. Elsevier Masson SAS; 2017. p. 171–5.

11. Chen JX, Lindeborg M, Herman SD, Ishai R, Knoll RM, Remenschneider A, et al. Systematic review of hearing loss after traumatic brain injury without associated temporal bone fracture. Am J Otolaryngol. 2018 May 1;39(3):338–44.

12. Livingston G, Huntley J, Sommerlad A, Ames D, Ballard C, Banerjee S, et al. Dementia prevention, intervention, and care: 2020 report of the Lancet Commission. The Lancet [Internet]. 2020 Aug 8;396(10248):413–46. Available from: https://doi.org/10.1016/S0140-6736(20)30367-6

13. Wilson BS, Tucci DL. Addressing the global burden of hearing loss. The Lancet [Internet]. 2021 Mar 13;397(10278):945–7. Available from: https://doi.org/10.1016/S0140-6736(21)00522-5

14. Monzani D, Galeazzi GM, Genovese E, Marrara A, Martini A. Psychological profile and social behaviour of working adults with mild or moderate hearing loss. Acta Otorhinolaryngologica Italica. 2008;28(2):61–6.

15. Šarkić B, Douglas JM, Simpson A. A cross-sectional survey of non-specialist Australian audio-vestibular clinical practice for traumatic brain injury and rehabilitation. Brain Impairment [Internet]. 2023 Jan 6 [cited 2023 Jan 13];1–18. Available from: https://www.cambridge.org/core/product/identifier/S1443964622000353/type/journal_article

16. Eysenbach G. Improving the quality of web surveys: The Checklist for Reporting Results of Internet E-Surveys (CHERRIES). Vol. 6, Journal of Medical Internet Research. JMIR Publications Inc.; 2004.

For peer review only

For peer review only - http://bmjopen.bmj.com/site/about/guidelines.xhtml

17. Šarkić B, Douglas JM, Simpson A. Peripheral auditory dysfunction secondary to traumatic brain injury: a systematic review of literature. Brain Inj [Internet]. 2019 Jan 28;33(2):111–28. Available from: https://doi.org/10.1080/02699052.2018.1539868

18. Roup CM, Ross C, Whitelaw G. Hearing difficulties as a result of traumatic brain injury. J Am Acad Audiol. 2020;31(2):137–46.

19. Šarkić B, Douglas JM, Simpson A, Vasconcelos A, Scott BR, Melitsis LM, et al. Frequency of peripheral vestibular pathology following traumatic brain injury: a systematic review of literature. Vol. 60, International Journal of Audiology. Taylor and Francis Ltd.; 2020. p. 479–94.

20. Knoll RM, Lubner RJ, Brodsky JR, Wong K, Jung DH, Remenschneider AK, et al. Auditory Quality-of-Life Measures in Patients With Traumatic Brain Injury and Normal Pure Tone Audiometry. Otolaryngology - Head and Neck Surgery (United States). 2020 Dec 1;163(6):1250–4.

21. Cotrena C, Branco LD, Zimmermann N, Cardoso CO, Grassi-Oliveira R, Fonseca RP. Impaired decision-making after traumatic brain injury: The Iowa Gambling Task. Brain Inj [Internet]. 2014 Jul 1;28(8):1070–5. Available from: https://doi.org/10.3109/02699052.2014.896943

22. Peeters W, van den Brande R, Polinder S, Brazinova A, Steyerberg EW, Lingsma HF, et al. Epidemiology of traumatic brain injury in Europe. Acta Neurochir (Wien) [Internet]. 2015;157(10):1683–96. Available from: https://doi.org/10.1007/s00701-015-2512-7

23. Teitcher JEF, Bockting WO, Bauermeister JA, Hoefer CJ, Miner MH, Klitzman RL. Detecting, Preventing, and Responding to “Fraudsters” in Internet Research: Ethics and Tradeoffs. The Journal of Law, Medicine & Ethics [Internet]. 2015 Apr 1;43(1):116–33. Available from: https://doi.org/10.1111/jlme.12200

For peer review only

FIGURE LEGENDS

Figure 1. The frequency of audio-vestibular symptoms reported by patients to HCPs.

For peer review only - http://bmjopen.bmj.com/site/about/guidelines.xhtml

Figure 2. The frequency of audio-vestibular symptoms reported by patients or their relatives to HCPs and/or observed by HCPs.

Figure 3. Percent of screening methods used by HCPs when they notice dizziness or balance disorders in patients with TBI.

Figure 4. Percent of reasons for HCPs to refer TBI patients to the ENT and/or audiology department.

SUPPLEMENTAL MATERIALS

Supplemental Appendix 1. English and Turkish versions of the survey

Supplemental Appendix 2. List of organisations that were contacted to share the surveys

Supplemental Appendix 3. The results of statistical analysis on associations between audio-vestibular symptoms observed by HCPs or reported by patients' relatives to HCPs, and the years working with TBI adults, groups of country of residence and occupations

For peer review only

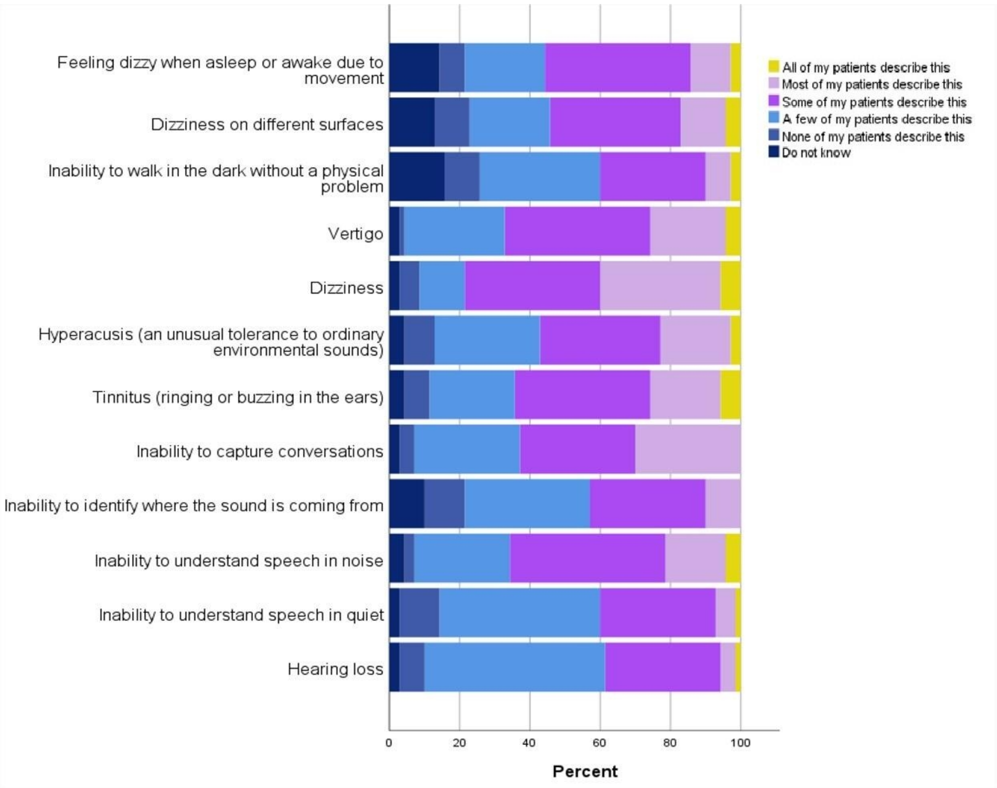

Figure 1. The frequency of audio-vestibular symptoms reported by patients to HCPs

52x41mm (600 x 600 DPI)

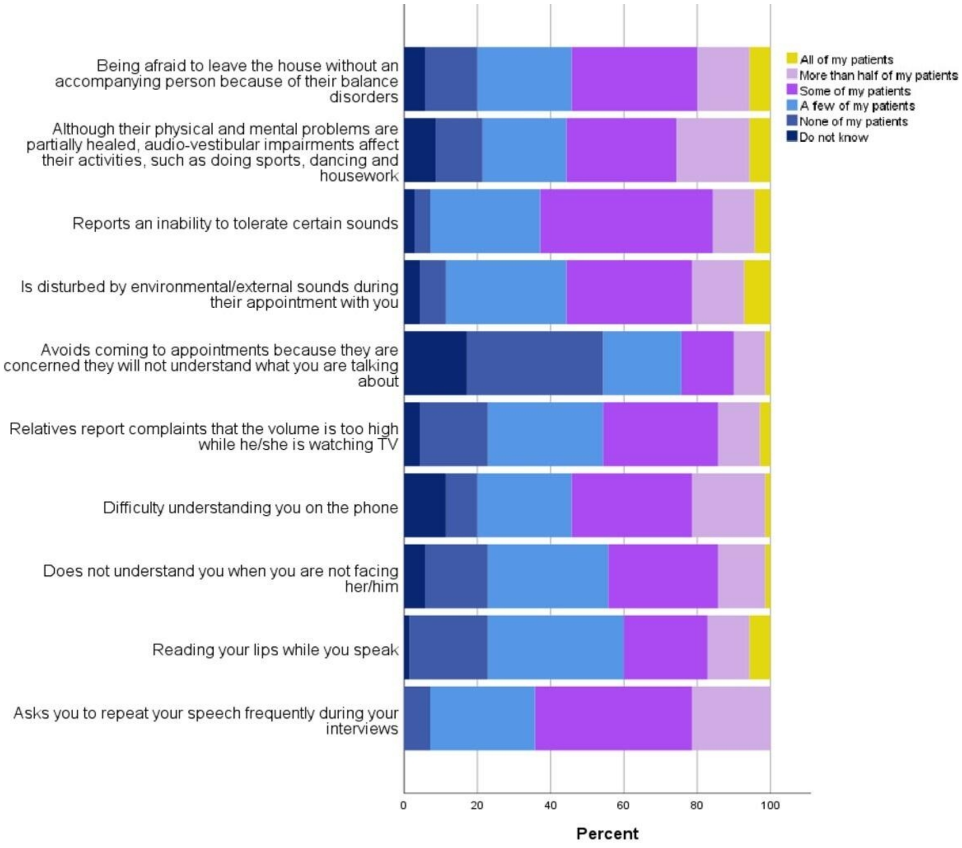

Figure 2. The frequency of audio-vestibular symptoms reported by patients or their relatives to HCPs and/or observed by HCPs

51x43mm (600 x 600 DPI)

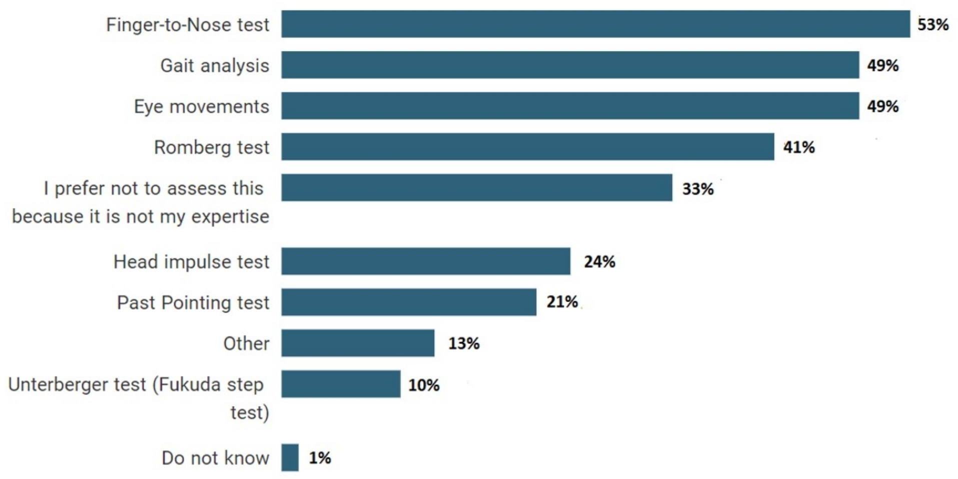

Figure 3. Percent of screening methods used by HCPs when they notice dizziness or balance disorders in patients with TBI

50x24mm (600 x 600 DPI)

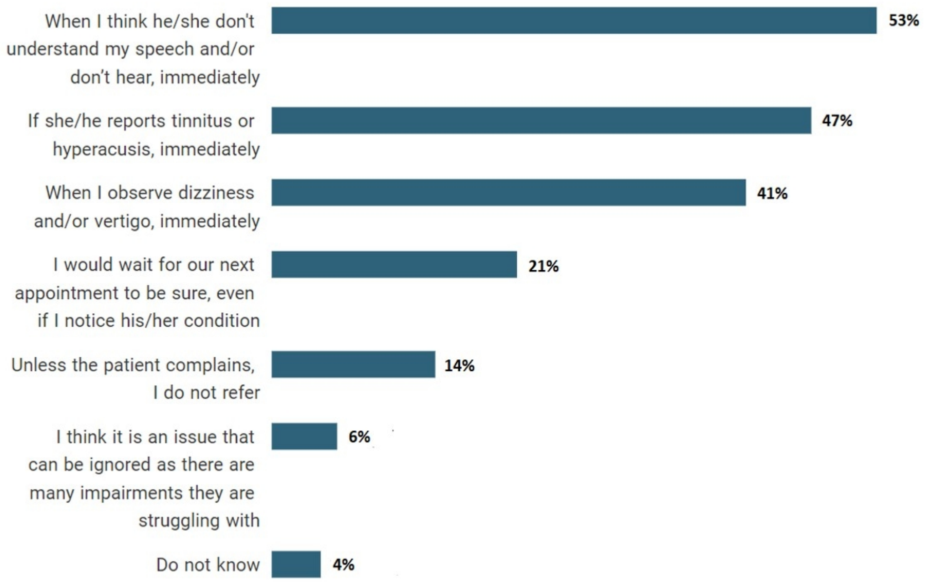

Figure 4. Percent of reasons for HCPs to refer TBI patients to the ENT and/or audiology department.

50x30mm (600 x 600 DPI)

Supplemental Appendix 1. English and Turkish version of the survey

English Version of Survey

Healthcare professionals and Audio- vestibular consequences of Traumatic Brain Injury

Page 1: Information and Consent Page for an OnlineSurvey/Questionnaire

Information and Consent page for an Online Survey/Questionnaire

Study Title: Experiences and opinions of healthcare professionals about the audio-vestibular findings of individuals with traumatic brain injury

Research Team:

Name of supervisors:

Kathryn Fackrell, Senior Research Fellow

Laura Edwards, Clinical Associate Professor in the Faculty of Medicine and Health Sciences

Co-investigator:

Kubra Bolukbas, PhD in Hearing Sciences

Faculty of Medicine and Health Sciences Research Ethics Ref: FMHS 462-0222

For peer review only

This study investigates the opinions, experiences and approaches of healthcare professionals ***(excluding ENT/audiology specialists)*** who deal with adults with traumatic brain injury, with a focus on the auditory and balance status of these patients.

For peer review only

Thank you for your interest. You are invited to take part because you are a healthcare professional ***(excluding ENT/audiology specialists)*** who works with adult traumatic brain injury patients. Please read through the following information before agreeing to participate. You can ask any questions before deciding by contacting the researchers (details below). Taking part is entirely voluntary.

What will I be asked to do?

After clicking the 'next' button at the end of this information page you will be asked to provide informed consent to participate in this study. You will then be asked to provide some basic demographic information related to your profession (i.e. profession, years of occupation, type of institution) and will also answer some questions about your experiences, opinions, and approaches in relation to audio-vestibular findings of traumatic brain injury patients. It should take you a maximum of 20 minutes to complete. We would like you to answer all questions as honestly and completely as possible. You can withdraw at any point during the questionnaire for any reason, before submitting your answers by clicking the Exit button/closing the browser. The data will only be uploaded on completion of the questionnaire by clicking the FINISH button on the final page. At this point, it will not be possible to withdraw your answers.

What are the disadvantages of taking part?

We are asking you to give up your valuable time to take part and this is appreciated.

What are the advantages of taking part?

Your contribution, along with others, will contribute to researchers' understanding of the awareness of healthcare professionals about the auditory and balance status of patients with traumatic brain injury. It will also contribute to the understanding of the international determination of the approaches to audio-vestibular problems in these patients. Future research may also be conducted based on the results of this study.

Who will know I have taken part in the study?

No one will know you have taken part in this study because we will not ask for your name or any other personal identification details (ID) during this survey. Your IP address will not be visible to or stored by the research team because an online survey platform is being used which receives and stores an IP address but enables this detail to be filtered out before it is transferred to the research team. As with any online related activity, the risk of breach is possible but this risk is being minimised by using a platform on an encrypted webpage.

For peer review only

What will happen to your data?

When you have clicked the submit button at the end of the questionnaire, it will be uploaded into a password-protected database with a code number. The research team will not be able to see who it is from and for this reason, it will not be possible to withdraw the data at this point. Your data (research data) will be stored in a password-protected folder on a restricted-access server at the university under the terms of its data protection policy. Data is kept for a minimum of 7 years and then destroyed.

This survey is for a PhD project and the answers received from all participants will be combined in a password-protected database ready for analysis. The results will be written up as a dissertation and may be used in academic publications and presentations. The overall anonymised data from this study may be shared for use in future research and teaching (with research ethics approval).

The only personal data we will receive is your e-mail if you contact us to ask further questions or need support. This will be received and handled separately from your completed questionnaire and it will not be possible to link the sets of data. Your e-mail address will only be kept as long as needed to resolve your problem. It will then be destroyed. For further information about how the university processes personal data please see: https://www.nottingham.ac.uk/utilities/privacy.aspx/

Who will have access to your data?

The University of Nottingham is the data controller (legally responsible for data security) and the Supervisor of this study (named above) is the data custodian (manages access to the data) and as such will determine how your data is used in the study. Your research and personal data will be used for the purposes of the research only. Research is a task that we perform in the public interest.

Responsible members of the University of Nottingham may be given access to data for monitoring and/or audit of the study to ensure it is being carried out correctly.

If you have any questions or concerns about this project, please contact:

Kubra Bolukbas, E-mail: kubra.bolukbas@nottingham.ac.uk or

if you have any concerns about any aspect of this study please contact the Research Supervisor:

Kathryn Fackrell, Email: kathryn.fackrell@nottingham.ac.uk;

Laura Edwards, Email: laura.edwards@nottingham.ac.uk

For peer review only

If you remain unhappy and wish to complain formally, you should then contact the FMHS Research Ethics Committee Administrator E-mail:

FMHS-ResearchEthics@nottingham.ac.uk

For peer review only

Page 2: Consent Form

Please confirm each box to continue. If you do not accept the consent form, please close the browser. ✱ *Required*

Please select at least 5 answer(s).

- ☐ I confirm that I have read and understood the information on the previous page
- ☐ I am a healthcare professionals dealing with adult traumatic brain injury patients (other than audiologist and/or ENT specialist)
- ☐ I understand that my participation is voluntary and I can end the study at any time and withdraw my data by clicking the EXIT button
- ☐ I understand that my answers are anonymous
- ☐ I understand the overall anonymized data from this study may be used in the future for research (with research ethics approval) and teaching purposes

For peer review only

Page 3: Experiences and opinions of healthcare professionals about the audio-vestibular findings of individuals with traumatic brain injury

1. What is your profession? * Required

- Neurologist
- Neuropsychiatrist
- Neurosurgeon
- General Surgeon
- Physiatrist or Rehabilitation Medicine Doctor
- Geriatrician
- Orthopaedic Surgeon
- Rehabilitation Nurse
- Neuropsychologist
- Speech and Language Therapist
- Occupational Therapist
- Physiotherapist
- Psychologist
- Other

1.a. If you selected Other, please specify:

2. How many years have you been in your occupation? * Required

- Less than 5 years
- 5-10 years
- 10-20 years

- More than 20 years

3. How many years have you been working with adult patients with traumatic brain injury? ✱ *Required*

- Less than 5 years
- 5-10 years
- 10-20 years
- More than 20 years

4. What country do you work in? ✱ *Required*

- Australia
- Azerbaijan
- Belgium
- Canada
- China
- Cyprus
- Denmark
- France
- Germany
- Greece
- India
- Ireland
- Israel
- Italy
- Japan
- Netherlands
- Poland

- Saudi Arabia
- Spain
- Sweden
- Switzerland
- Thailand
- Türkiye
- United Kingdom
- United States of America
- Other

4.a. If you selected Other, please specify:

5. Is your place of work private or state-owned? * *Required*

- Private
- State-owned

6. How many hours per week do you spend working with traumatic brain injury adult patients? **Required*

- Less than an hour
- 1-5 hours
- 5-10 hours
- 10-20 hours
- More than 20 hours

For peer review only

7. The following symptoms may be reported by people who have had a traumatic brain injury. Please indicate how frequently your adult patients, who experienced trauma more than 6 months ago, describe these to you ✱ *Required*

Please don't select more than 1 answer(s) per row.

Please select at least 12 answer(s).

	All of my patients describe this	Most of my patients describe this	Some of my patients describe this	A few of my patients describe this	None of my patients describe this	Do not know
Hearing loss	☐	☐	☐	☐	☐	☐
Inability to understand speech in quiet	☐	☐	☐	☐	☐	☐
Inability to understand speech in noise	☐	☐	☐	☐	☐	☐
Inability to identify where the sound is coming from	☐	☐	☐	☐	☐	☐
Inability to capture conversations	☐	☐	☐	☐	☐	☐
Tinnitus (ringing or buzzing in the ears)	☐	☐	☐	☐	☐	☐
Hyperacusis (an unusual tolerance to ordinary environmental sounds)	☐	☐	☐	☐	☐	☐
Dizziness	☐	☐	☐	☐	☐	☐
Vertigo	☐	☐	☐	☐	☐	☐

Inability to walk in the dark without a physical problem	☐	☐	☐	☐	☐	☐
Dizziness on different surfaces	☐	☐	☐	☐	☐	☐
Feeling dizzy when asleep or awake due to movement	☐	☐	☐	☐	☐	☐

8. The following symptoms may be reported and/or observed by people who have had a traumatic brain injury. Please indicate how frequently, adults who experienced trauma more than 6 months ago, describe these to you, *Required

Please don't select more than 1 answer(s) per row.

Please select at least 10 answer(s).

	All of my patients	More than half of my patients	Some of my patients	A few of my patients	None of my patients	Do not know
Asks you to repeat your speech frequently during your interviews	☐	☐	☐	☐	☐	☐
Reading your lips while you speak	☐	☐	☐	☐	☐	☐
Does not understand you when you are not facing her/him	☐	☐	☐	☐	☐	☐
Difficulty understanding you on the phone	☐	☐	☐	☐	☐	☐

For peer review only - http://bmjopen.bmj.com/site/about/guidelines.xhtml

Relatives report complaints that the volume is too high while he/she is watching TV	☐	☐	☐	☐	☐	☐
Avoids coming to appointments because they are concerned they will not understand what you are talking about	☐	☐	☐	☐	☐	☐
Is disturbed by environmental/external sounds during their appointment with you	☐	☐	☐	☐	☐	☐
Reports an inability to tolerate certain sounds	☐	☐	☐	☐	☐	☐
Although their physical and mental problems are partially healed, audio-vestibular impairments affect their activities, such as doing sports, dancing and housework	☐	☐	☐	☐	☐	☐
Being afraid to leave the house without an accompanying person because of their balance disorders	☐	☐	☐	☐	☐	☐

9. Do you ask your adult patients with traumatic brain injury (which occurred more than 6 months ago) about their balance status (dizziness, vertigo etc.) at your appointments? * *Required*

- Yes, I ask everybody
- If their balance status prevents me from performing our appointments and/or my job, I ask
- I prefer not to ask because it is not my expertise
- I do not ask anything if there are no complaints about it
- Do not know

10. If you notice dizziness or balance disorders in your adult patients with traumatic brain injury (which occurred more than 6 months ago), which of the screening methods would you use? You can choose multiple options ✱ *Required*

- [ ] Past Pointing test
- [ ] Finger-to-Nose test
- [ ] Unterberger test (Fukuda step test)
- [ ] Romberg test
- [ ] Gait analysis
- [ ] Eye movements
- [ ] Head impulse test
- [ ] I prefer not to assess this because it is not my expertise
- [ ] Do not know
- [ ] Other

10.a. If you selected Other, please specify:

11. In which situation would you consider referring your traumatic brain injury patient (where the trauma occurred more than 6 months ago) to the ENT and/or audiology service? You can choose multiple options ✱ *Required*

For peer review only - http://bmjopen.bmj.com/site/about/guidelines.xhtml

- [ ] When I think he/she don't understand my speech and/or don’t hear, immediately
- [ ] When I observe dizziness and/or vertigo, immediately
- [ ] If she/he reports tinnitus or hyperacusis, immediately
- [ ] I would wait for our next appointment to be sure, even if I notice his/her condition
- [ ] Unless the patient complains, I do not refer
- [ ] I think it is an issue that can be ignored as there are many impairments they are struggling with
- [ ] Do not know

12. How many of your adult patients who have had a traumatic brain injury (which occurred more than 6 months ago) do you refer to the audiology or ENT department? *Required*

- All of my patients
- More than half of my patients
- Some of my patients
- A few of my patients
- None of my patients
- Do not know

13. How do you check the external ear condition of your adult patients with traumatic brain injury (which occurred more than 6 months ago)? *Required*

- Referring to the ENT and/or audiology service
- I prefer to evaluate it myself with otoscopy
- I do not do anything if there are no complaints about it
- I am not interested because it is not my area of expertise
- Do not know

For peer review only

14. Do you think that your traumatic brain injury patients (where the trauma occurred more than 6 months ago) should use hearing aids if they have hearing loss?
* Required

- Yes, definitely
- If they would like, yes
- No need
- I think it is an issue that can be ignored as there are many impairments they are struggling with
- Do not know

15. In which situations can you help your adult patients with traumatic brain injury, who use hearing aids, during their appointment with you? You can choose multiple options
* Required

- [ ] I can change the battery of hearing aids
- [ ] I can notice whether or not it is working
- [ ] I can notice any feedback problem
- [ ] I can check the hearing aid ear mould and/or dome functionality
- [ ] I do not know anything about hearing aids

16. Did your patient group, who frequently report auditory and/or vestibular complaints after trauma, have a specific aetiology of traumatic brain injury? Can you generalize the trauma aetiology of these patients with auditory and/or vestibular complaints?
* Required

- Yes
- No
- Do not know

For peer review only

16.a. If you answered yes to question 16, what was this aetiology?

- ○ Falls
- ○ Vehicle-related collisions
- ○ Violence
- ○ Sport Injuries
- ○ Other Trauma (Please describe)

16.a.i. If you selected Other, please specify:

For peer review only

Turkish Version of Survey

Sağlık Çalışanları ve Travmatik Beyin Hasarının İşitsel-vestibüler Sonuçları

Page 1: Bilgilendirme Formu

Bilgilendirme ve Onam Formu

Final version 1.2: 04.07.2022

Araştırma Başlığı: Travmatik beyin hasarı olan bireylerin odyo-vestibüler bulgularına ilişkinsağlık profesyonellerinin deneyimleri ve görüşleri

Araştırma Ekibi:

Danışmanlar:

Dr. Kathryn Fackrell, İşitme Bilimleri

Dr. Laura Edwards, Tıp ve Sağlık Bilimleri

Araştırmacı:

Uzm Ody. Kübra Bölükbaş, İşitme Bilimleri Doktora Öğrencisi

Tıp ve Sağlık Bilimleri Fakültesü Araştırma Etiği Ref. No: FMHS 462-0222

Bu çalışma, ***KBB ve odyoloji uzmanlarının dışındaki***, travmatik beyin hasarı olan erişkinlerleilgilenen sağlık profesyonellerinin görüş, deneyim ve yaklaşımlarını bu hastaların işitsel ve denge durumlarına odaklanarak incelemektedir.

İlgiliniz için teşekkür ederiz. ***KBB/odyoloji uzmanları dışında*** travmatik beyin hasarı olan yetişkin hastalarla ilgilenen bir sağlık çalışanı olduğunuz için çalışmamıza katılmaya davetlisiniz. Lütfen katılımcı olmadan önce bilgilendirme ve onam formunu dikkatlice okuyunuz.Herhangi bir sorunuz varsa aşağıda yer alan iletişim kanalları

For peer review only

aracılığıyla araştırmacılara ulaşmaktan lütfen çekinmeyin. Araştırmaya katılım gönüllülük esasına dayanmaktadır.

For peer review only

Ne yapmanızı isteyeceğiz?

Bilgilendirme sayfasının sonundaki "Next (İleri)" butonuna tıkladıktan sonra, bu çalışmaya katılabilmeniz için onam formunu kabul etmeniz istenecektir. Daha sonra, mesleğinizle ilgili demografik bilgiler (mesleğiniz, çalışma süreniz, kurum türü gibi) içeren birkaç soru ve travmatikbeyin hasarı olan yetişkin hastalarınızın işitsel-vestibüler bulgularıyla ilgili deneyimleriniz, görüşleriniz ve yaklaşımlarınızı öğrenmek amacıyla hazırlanan soruları cevaplamanız istenecektir. Tüm anket sorularını cevaplamanız en fazla yirmi (20) dakikanızı alacaktır. Tüm soruları mümkün olduğunca dürüst ve eksiksiz olarak cevaplamanızı rica ediyoruz.

Cevaplarınızı göndermeden önce, "Çıkış" butonuna tıklayarak ve/veya tarayıcınızı kaparatarak herhangi bir noktada, herhangi bir nedenle anketten çekilebilirsiniz. Ancak, son

sayfada bulunan "Bitir (Finish)" butonuna tıkladıktan sonra cevaplarınız tarafımıza gönderilecekve cevaplarınızı geri çekmeniz mümkün olmayacaktır.

Katılımcı olmanızın dezavantajları nelerdir?

Katılımcı olmanız için kıymetli zamanınızdan birkaç dakikanızı anketimize ayırmanız gerekecektir.

Katılımcı olmanızın avantajları nelerdir?

Diğer katılımcılar ve sizin katkınız sayesinde araştırmacılar, travmatik beyin hasarı olan bireylerin işitsel ve vestibüler durumları hakkında KBB ve odyoloji uzmanları haricindeki sağlık uzmanlarının görüşleri, yaklaşımları ve farkındalıkları hakkında bilgi edineceklerdir. Ayrıca, bu bireylerdeki işitsel-vestibüler sorunlara yaklaşımın uluslararası düzeyde nasıl olduğunun tespit edilmesine katkı sağlanacaktır. Böylece, çalışmanın sonuçlarından yola çıkılarak konuya ilişkingelecek araştırmaların da önü açılabilir.

Araştırmacılar çalışmaya katıldığınızı bilecekler mi?

İsim, soyisim ve kimlik bilgisi gibi kişisel verileriniz sorulmadığı için hiç kimse araştırmaya katıldığınızı bilmeyecektir. Ayrıca, cevaplarınız araştırma ekibine iletilirken kullanılan platformunfiltreleme sistemi sayesinde IP adresiniz gözükmeyecek ve saklanmayacaktır. Tüm çevrimiçi platformlarda ihlal riski mümkün olduğu gibi bu platformda da mümkündür. Ancak kişisel veri alınmaması ve şifreli bir sistemin kullanılması sayesinde bu risk minimuma düşürülmüştür.

Verilerinize ne olacak?

Anketin sonundaki "Bitir (Finish)" butonuna tıkladığınızda, anket sonuçlarınız şifre korumalı birveri tabanına kod numarası ile yüklenecektir. Araştırma ekibi anketin kim tarafından cevaplandığını göremeyecek ve bu nedenle, bu noktada, verileri geri çekmeniz

For peer review only

mümkün olmayacaktır. Araştırma verileriniz, Nottingham Üniversitesinin veri koruma politikasışartlarına göre sınırlı erişimli bir sunucuda şifre korumalı bir klasörde saklanacaktır. Veriler enaz 7 yıl saklandıktan sonra imha edilecektir.

Bu anket bir doktora projesi için hazırlanmıştır. Tüm katılımcılardan elde edilen yanıtlar analizedilmeye hazır şifre korumalı bir veritabanında birleştirilecektir. Sonuçlar doktora kapsamındabir tez olarak yazılmasının yanısıra akademik yayınlarda ve sunumlarda kullanılabilir. Bu çalışmadan elde edilen anonimleştirilmiş veriler, etik onayı ile gelecekteki araştırma ve öğrenimde kullanılmak üzere paylaşılabilir.

Daha fazla soru sormak için veya desteğe ihtiyaç duyduğunuzda bizimle iletişime geçerseniz, alacağımız tek kişisel veri e-postanız olacaktır. Ancak bu, tamamlamış olduğunuz anketinizdenayrı olarak ele alınacaktır ve verilerinizi birbirine bağlamak mümkün olmayacaktır. E-posta adresiniz yalnızca sorununuzu çözmek için gerektiği kadar saklanacak ve daha sonra imha edilecektir. Üniversitenin kişisel verileri nasıl işlediği hakkında daha fazla bilgi için lütfen şu adresi ziyaret ediniz: https://www.nottingham.ac.uk/utilities/privacy.aspx/

Verilerinize kim erişebilecek?

Nottingham Üniversitesi veri denetleyicisidir (veri güvenliğinden yasal olarak sorumludur) ve buçalışmanın danışmanları (yukarıda adı geçen) veri sorumlusudur (verilere erişimi yönetmektedirler) ve bu nedenle verilerinizin çalışmada nasıl kullanılacağını belirleyeceklerdir. Yanıtlarınız ve verileriniz yalnızca araştırma amacıyla kullanılacaktır. Araştırmamız, kamu yararını gözeterek gerçekleştirdiğimiz bir projedir.

Nottingham Üniversitesi'nin sorumlu üyelerine, çalışmanın doğru bir şekilde yürütüldüğündenemin olmak için izleme ve/veya denetleme için verilere erişim izni verilebilir.

Bu projeyle ilgili herhangi bir sorunuz veya endişeniz varsa, lütfen iletişime geçiniz:

Kübra Bölükbaş, E-mail: kubra.bolukbas@nottingham.ac.uk or

Bu çalışmanın herhangi bir yönü hakkında bir endişeniz varsa, lütfen araştırma danışmanları ileiletişime geçiniz:

Kathryn Fackrell,

Email: kathryn.fackrell@nottingham.ac.uk

Laura Edwards,

Email: laura.edwards@nottingham.ac.uk

For peer review only

Resmi olarak şikayette bulunmak isterseniz, FMHS Araştırma Etik Kurulu Yöneticisi ile iletişimegeçiniz:

FMHS-ResearchEthics@nottingham.ac.uk

For peer review only

Page 2: Onam Formu

Please select at least 5 answer(s).

- ☐ Önceki sayfadaki bilgileri okuduğumu ve anladığımı onaylıyorum
- ☐ Odyolog ve/veya KBB uzmanı dışında, travmatik beyin hasarı olan yetişkinlerle ilgilenen bir sağlık çalışanıyım
- ☐ Katılımımın gönüllü olduğunu ve çalışmayı istediğim zaman sonlandırabileceğimi ve sayfayı kapatarak verilerimi geri çekebileceğimi anlıyorum
- ☐ Yanıtlarımın anonim olarak görüneceğini anlıyorum
- ☐ Bu çalışmadan elde edilen anonimleştirilmiş verilerin gelecekte etik onayı ile araştırma ve öğrenim amaçları için kullanılabileceğini anlıyor ve kabul ediyorum

Devam edebilmek için lütfen her bir kutucuğu onaylayın. Bilgilendirme ve onam formunukabul etmiyorsanız, lütfen tarayıcınızı kapatarak çıkış yapınız. ✱*Required*

For peer review only

Page 3: Travmatik Beyin Hasarı olan Bireylerin Odyo-vestibülerBulgularına İlişkin Sağlık Profesyonellerinin Deneyimleri ve Görüşleri

1. Mesleğiniz nedir? ✱ *Required*

- Nörolog
- Nöropsikiyatrist
- Beyin Cerrahı
- Genel Cerrahi Uzmanı
- Fiziksel Tıp ve Rehabilitasyon Uzmanı
- Geriatri Uzmanı
- Ortopedi ve Travmatoloji Uzmanı
- Hemşire
- Nöropsikolog
- Dil ve Konuşma Terapisti
- Ergoterapist
- Fizyoterapist
- Psikolog
- Diğer Sağlık Profesyoneli (Lütfen açıklayınız)

1.a. Diğer seçeneğini seçtiyseniz, lütfen açıklayın:

2. Kaç yıldır mesleğinizi yapıyorsunuz?✱ *Required*

- 5 yıldan az
- 5 ile 10 yıl arası
- 10 ile 20 yıl arası
- 20 yılı aşkın süredir

3. Travmatik beyin hasarı olan hastalarla kaç yıldır çalışıyorsunuz? ✱ Required

- 5 yıldan az
- 5 ile 10 yıl arası
- 10 ile 20 yıl arası
- 20 yılı aşkın süredir

4. Hangi ülkede çalışıyorsunuz? ✱ Required

- Almanya
- Amerika
- Avustralya
- Azerbaycan
- Belçika
- Birleşik Krallık
- Çin
- Danimarka
- Hindistan
- Hollanda
- İrlanda
- İspanya
- İsrail
- İsveç
- İsviçre
- İtalya
- Japonya
- Kanada
- Kıbrıs
- Polonya
- Suudi Arabistan
- Türkiye

- Yunanistan
- Diğer

4.a. Diğer seçeneğini seçtiyseniz, lütfen açıklayın:

5. İş yeriniz özel mi yoksa devlete mi ait?✱ *Required*

- Özel
- Devlet

6. Travmatik beyin hasarı olan yetişkin hastalarla haftada kaç saat çalışıyorsunuz? ✱ *Required*

- 1 saatten az
- 1 ile 5 saat arası
- 5 ile 10 saat arası
- 10 ile 20 saat arası
- 20 saatten fazla

7. Aşağıdaki şikayetler travmatik beyin hasarı geçirmiş kişiler tarafından bildirilebilir. Lütfen 6 aydan daha uzun süre önce kafa travmasına maruz kalmış hastalarınızın bunları size ne sıklıklatarif ettiğini belirtiniz. ✱ *Required*

Please don't select more than 1 answer(s) per row.

Please select at least 12 answer(s).

For peer review only

	Bütün hastaları mbunu bildiriyor	Hastalarımı nçoğu bunu bildiriyor	Bazı hastaları mbunu bildiriyor	Birkaç hastam bunu bildiriyor	Hiçbir hastam bunu bildirmiyor	Bilmiyorum
İşitme kaybı	☐	☐	☐	☐	☐	☐
Sessiz ortamda konuşmayı anlayamama	☐	☐	☐	☐	☐	☐
Gürültülü ortamda konuşmayı anlayamama	☐	☐	☐	☐	☐	☐
Sesin nereden geldiğini tespit edememe	☐	☐	☐	☐	☐	☐
Konuşmaları yakalayamama	☐	☐	☐	☐	☐	☐
Kulak çınlaması (Tinnitus)	☐	☐	☐	☐	☐	☐
Normal çevresel seslere karşı olağan dışı hassasiyet, tahammülsüzlük (Hiperakuzi)	☐	☐	☐	☐	☐	☐
Dengesizlik, Sersemlik (Dizziness)	☐	☐	☐	☐	☐	☐
Baş Dönmesi (Vertigo)	☐	☐	☐	☐	☐	☐
Fiziksel bir problemi olmaksızın karanlıkta yürüyememe	☐	☐	☐	☐	☐	☐

Farklı yüzeylerde yürürken dengesizlik (dizziness)	☐	☐	☐	☐	☐	☐
Harekete bağlı olarak uykuda veya uyanıkken baş dönmesi hissetmek	☐	☐	☐	☐	☐	☐

8. Aşağıdaki belirtiler travmatik beyin hasarı geçirmiş kişiler tarafından bildirilebilir ve/veyagözlemlenebilir. Lütfen 6 aydan daha uzun süre önce kafa travmasına maruz kalmış hastalarınızın bunları size ne sıklıkla bildirdiğini belirtiniz ✱*Required*

Please don't select more than 1 answer(s) per row.

Please select at least 10 answer(s).

	Hastalarımın hepsi	Hastalarımın yarısından fazlası	Bazı hastalarım	Birkaç hastam	Hastalarımın hiçbiri bunu bildirmiyor	Bilmiyorum
Görüşmeleriniz sırasında konuşmanızı sıksık tekrarlamanızı ister	☐	☐	☐	☐	☐	☐
Konuşurken dudaklarınızı okur	☐	☐	☐	☐	☐	☐
Yüzünüz ona dönük olmadığında sizianlamaz	☐	☐	☐	☐	☐	☐
Telefonda sizi anlamakta zorluk çeker	☐	☐	☐	☐	☐	☐

For peer review only

Hasta yakınları hastanın çok yüksek sesle televizyon izlemeye başladığından şikayet eder	☐	☐	☐	☐	☐	☐
Ne hakkında konuştuğunuzu anlayamamaktan endişelendiği için randevularınıza gelmekten kaçınır	☐	☐	☐	☐	☐	☐
Sizinle görüşmesi sırasında çevresel/dış seslerden rahatsız olur	☐	☐	☐	☐	☐	☐
Belirli seslere tahammül edemediğini bildirir	☐	☐	☐	☐	☐	☐
Fiziksel ve zihinsel sorunları kısmen iyileşmiş olsa da, işitsel-vestibüler bozukluklar spor yapma, dans etme ve ev işi yapma gibi aktivitelerini etkiler	☐	☐	☐	☐	☐	☐

Denge bozuklukları nedeniyle refakatçi olmadan evden çıkmaya korkar	☐	☐	☐	☐	☐	☐

9. 6 aydan daha uzun süre önce meydana gelen travmatik beyin hasarına maruz kalmış hastalarınıza randevularınızda denge durumlarının nasıl olduğunu (sersemlik, başdönmesi vb.) soruyor musunuz? ✱*Required*

- ◯ Evet, tüm hastalarıma sorarım
- ◯ Denge durumları randevularımızı etkiliyorsa ve/veya işimi yapmamı engelliyorsa sorarım
- ◯ Bu konu uzmanlık alanım olmadığı için sormamayı tercih ediyorum
- ◯ Bu konuda herhangi bir şikayeti yoksa hiçbir şey sormam
- ◯ Bilmiyorum

10. 6 aydan daha uzun süre önce travmatik beyin hasarına maruz kalan hastalarınızda baş dönmesi ve/veya denge bozukluğu fark ederseniz, aşağıdaki tarama yöntemlerinden hangisiniveya hangilerini kullanırsınız? Birden fazla yanıt seçebilirsiniz. ✱ *Required*

- ☐ Past Pointing testi
- ☐ Parmak Burun testi
- ☐ Unterberger testi (Fukuda adım
- ☐ testi)Romberg testi
- ☐ Gait analysis (Yürüme analizi)
- ☐ Göz hareketleri
- ☐ Head impulse testi
- ☐ Uzmanlık alanım olmadığı için değerlendirmeyi tercih etmiyorum
- ☐ Bilmiyorum
- ☐ Diğer

10.a. Diğer seçeneğini seçtiyseniz, lütfen açıklayın:

11. 6 aydan daha uzun süre önce travmatik beyin hasarına maruz kalmış hastanızı hangidurum veya durumlarda KBB ve/veya odyoloji departmanına sevk etmeyi düşünürsünüz? Birden fazla yanıt seçebilirsiniz.✱ *Required*

- ☐ Konuşmamı anlamadığını ve/veya duymadığını düşündüğümde hemen
- ☐ Dizziness ve/veya baş dönmesi gözlemlediğimde hemen
- ☐ Kulak çınlaması veya seslere karşı hassasiyet bildirirse hemen
- ☐ Durumunu fark etsem bile emin olmak için bir sonraki randevuyu beklerim
- ☐ Hasta herhangi bir şikayet bildirmedikçe sevk etmiyorum
- ☐ Bu hasta grubunun uğraştığı çok fazla problemi olduğu için görmezden gelinebilecek bir konu olduğunu düşünüyorum
- ☐ Bilmiyorum

review

12. 6 aydan daha uzun süre önce travmatik beyin hasarına maruz kalmış hastalarınızdantahmini olarak kaç tanesini odyoloji ve/veya KBB departmanına yönlendiriyorsunuz? ✱*Required*

- ○ Hastalarımın hepsini
- ○ Hastalarımın yarısından
- ○ fazlasınıBazı hastalarımı
- ○ Birkaç hastamı
- ○ Hastalarımın hiçbirini yönlendirmiyorum
- ○ Bilmiyorum

13. 6 aydan daha uzun süre önce travmatik beyin hasarına maruz kalmış hastalarınızın gerektiğinde, dış kulaklarının durumunu nasıl kontrol edersiniz? ✱ *Required*

- ○ KBB hekimine ve/veya odyoloji departmanına konsülte ederim

- Otoskop ile kendim değerlendirmeyi tercih ederim
- Bu konuda şikayeti yoksa hiçbir şey yapmam
- Uzmanlık alanım olmadığı için ilgilenmiyorum
- Bilmiyorum

14. 6 aydan daha önce travmatik beyin hasarına maruz kalan hastanızın işitme kaybı varsaişitme cihazı kullanması gerektiğini düşünüyor musunuz? ✱ *Required*

- Evet, kesinlikle kullanmalı
- İsterse, evet
- Gerek yok
- Çok fazla problemle uğraştıkları için görmezden gelinebilecek bir konu olduğunu düşünüyorum
- Bilmiyorum

15. İşitme cihazı kullanan travmatik beyin hasarına maruz kalmış hastalarınıza randevularısırasında işitme cihazlarıyla ilgili hangi durumlarda yardımcı olabilirsiniz? Birden fazla yanıt seçebilirsiniz. ✱ *Required*

- İşitme cihazlarının pilini değiştirebilirim
- Hastamın işitme cihazının çalışıp çalışmadığını anlayabilirim
- Herhangi bir feedback (ötme sesi) sorunu varsa fark edebilirim
- İşitme cihazının kulak kalıbını ve/veya dome (kubbe) işlevini kontrol edebilirim
- İşitme cihazları hakkında hiçbir şey bilmiyorum

16. Travma sonrası sıklıkla işitsel ve/veya vestibüler şikayetler bildiren hasta grubunuz,travmatik beyin hasarının spesifik bir etiyolojisine sahip miydi? İşitsel ve/veya vestibüler şikayetleri olan bu hastaların travma etiyolojisini genelleyebilir misiniz ? ✱ *Required*

- Evet

- Hayır
- Bilmiyorum

16.a. 17. soruya evet cevabı verdiyseniz, bu etiyoloji neydi?

- Düşme
- Trafik kazası
- Şiddet
- Spor yaralanmaları
- Diğer nedenler(Lütfen açıklaynız)

16.a.i. Diğer seçeneğini seçtiyseniz, lütfen açıklayın:

For peer review only

Supplemental Appendix 2. List of organisations that were contacted to share the surveys

Name of Organisations	Country
Speech Pathology Australia	Australia
Rehabilitation Medicine Society of Australia and New Zealand	Australia and New Zealand
the Royal Australian and New Zealand College of Psychiatrist	Australia and New Zealand
Speech-Language & Audiology Canada	Canada
Hellenic Neuropsychological Society	Greece
Logopedista	Italy
Association of Occupational Therapists of Ireland	Ireland
Irish Association of Speech & Language Therapists	Ireland
Headway The Brain Injury Association	United Kingdom
The British Neuropsychological Society	United Kingdom
British Psychological Society	United Kingdom
The British Neuropsychiatry Association	United Kingdom
Northampton Speech Therapy (Independent Therapist)	United Kingdom
British Society of Rehabilitation Medicine	United Kingdom
Royal College of Speech and Language Therapists	United Kingdom
Association of Speech and Language Therapists	Türkiye
World Federation of Occupational Therapists	

For peer review only

Supplemental Appendix 3. The results of statistical analysis on associations between audio-vestibular symptoms observed by HCPs or reported by patients' relatives to HCPs, and the years working with TBI adults, groups of country of residence and groups of occupations.

A statistically significant difference was observed in the years of working with TBI adults, in the case of 'being afraid to leave the house without an accompanying person because of their balance disorders' in the Kruskal-Wallis test ($p<0.05$). The statistical differences were identified between the groups working with adults with TBI for 5-10 years and 10-20 years (Appendix 3-Table 1), as well as between the groups working with adults with TBI for 5-10 years and more than 20 years in the Mann-Whitney U test with Bonferroni correction ($p<0.008$; Appendix 3-Table 2).

Ranks

	How many years have you been working with adult patients with traumatic brain injury?	N	Mean Rank	Sum of Ranks
Being afraid to leave the house without an accompanying person because of their balance disorders	5-10 years	20	14.88	297.50
	10-20 years	19	25.39	482.50
	Total	39		

Test Statistics[a]

	Being afraid to leave the house without an accompanying person because of their balance disorders
Mann-Whitney U	87.500
Wilcoxon W	297.500
Z	-2.970
Asymp. Sig. (2-tailed)	.003
Exact Sig. [2*(1-tailed Sig.)]	.003[b]

a. Grouping Variable: How many years have you been working with adult patients with traumatic brain injury?

Appendix 3-Table 1: The comparison of 5-10 years and 10-20 years groups in the case of 'being afraid to leave the house without an accompanying person because of their balance disorders' via the Mann-Whitney U test with Bonferroni correction

Ranks

	How many years have you been working with adult patients with traumatic brain injury?	N	Mean Rank	Sum of Ranks
Being afraid to leave the house without an accompanying person because of their balance disorders	5-10 years	20	13.75	275.00
	More than 20 years	15	23.67	355.00
	Total	35		

Test Statistics[a]

	Being afraid to leave the house without an accompanying person because of their balance disorders
Mann-Whitney U	65.000
Wilcoxon W	275.000
Z	-2.934
Asymp. Sig. (2-tailed)	.003
Exact Sig. [2*(1-tailed Sig.)]	.004[b]

a. Grouping Variable: How many years have you been working with adult patients with traumatic brain injury?

Appendix 3-Table 2: The comparison of 5-10 years and more than 20 years groups in the case of 'being afraid to leave the house without an accompanying person because of their balance disorders' via the Mann-Whitney U test with Bonferroni correction

For peer review only

Majority of HCPs (n=13, 19%) who work with TBI adults for 5-10 years stated that ‘more than half' and ‘some’ of their patients reported ‘being afraid to leave the house without an accompanying person because of their balance disorders’, whilst most of the participants who work with TBI patients for 10-20 years (n=12, 17%) and more than 20 years (n=11, 16%) indicated that this case was observed in ‘some’ and ‘a few’ of their patients.

Furthermore, there were statistically significant differences for symptoms of ‘asking to repeat their speech frequently during their interviews’, ‘relatives reporting complaints that the volume is too high while he/she is watching TV’ and ‘avoiding coming to appointments because TBI patients are concerned they will not understand what HCPs are talking about’ based on groups of country of residence in the Kruskal-Wallis test ($p<0.05$). Statistically significant differences were noted between the UK and Asia for all three symptoms as seen in Appendix 3-Table 3.

Ranks

	What country do you work in?	N	Mean Rank	Sum of Ranks
Asks you to repeat your speech frequently during your interviews	Asia	15	15.00	225.00
	United Kingdom	30	27.00	810.00
	Total	45		
Relatives report complaints that the volume is too high while he/she is watching TV	Asia	15	12.87	193.00
	United Kingdom	30	28.07	842.00
	Total	45		
Avoids coming to appointments because they are concerned they will not understand what you are talking about	Asia	15	15.80	237.00
	United Kingdom	30	26.60	798.00
	Total	45		

Test Statistics[a]

	Asks you to repeat your speech frequently during your interviews	Relatives report complaints that the volume is too high while he/she is watching TV	Avoids coming to appointments because they are concerned they will not understand what you are talking about
Mann-Whitney U	105.000	73.000	117.000
Wilcoxon W	225.000	193.000	237.000
Z	-3.109	-3.803	-2.694
Asymp. Sig. (2-tailed)	.002	<.001	.007

a. Grouping Variable: What country do you work in?

Appendix 3-Table 3: The comparison of the UK and Asia groups in the cases of ‘asking to repeat their speech frequently during their interviews’, ‘relatives reporting complaints that the volume is too high while he/she is watching TV’ and ‘avoiding coming to appointments because TBI patients are concerned they will not understand what HCPs are talking about’ via the Mann-Whitney U test with Bonferroni correction

Similarly, differences were observed between Asia and Others for the symptoms ‘asking to repeat their speech frequently during their interviews’ and ‘relatives reporting complaints that the volume is too high while he/she is watching TV’ ($p<0.008$; Appendix 3-Table 4).

Ranks

	What country do you work in?	N	Mean Rank	Sum of Ranks
Asks you to repeat your speech frequently during your interviews	Asia	15	10.57	158.50
	Others	15	20.43	306.50
	Total	30		
Relatives report complaints that the volume is too high while he/she is watching TV	Asia	15	10.53	158.00
	Others	15	20.47	307.00
	Total	30		
Avoids coming to appointments because they are concerned they will not understand what you are talking about	Asia	15	12.33	185.00
	Others	15	18.67	280.00
	Total	30		

Test Statistics[a]

	Asks you to repeat your speech frequently during your interviews	Relatives report complaints that the volume is too high while he/she is watching TV	Avoids coming to appointments because they are concerned they will not understand what you are talking about
Mann-Whitney U	38.500	38.000	65.000
Wilcoxon W	158.500	158.000	185.000
Z	-3.197	-3.187	-2.045
Asymp. Sig. (2-tailed)	.001	.001	.041
Exact Sig. [2*(1-tailed Sig.)]	.001[b]	.001[b]	.050[b]

a. Grouping Variable: What country do you work in?

b. Not corrected for ties.

Appendix 3-Table 4: The comparison of the UK and Others groups in the cases of ‘asking to repeat their speech frequently during their interviews’, ‘relatives reporting complaints that the volume is too high while he/she is watching TV’ and ‘avoiding coming to appointments because TBI patients are concerned they will not understand what HCPs are talking about’ via the Mann-Whitney U test with Bonferroni correction

Additionally, a statistical difference was observed regarding the symptom 'relatives reporting complaints that the volume is too high while he/she is watching TV' between Europe and the UK in the Mann-Whitney U test with Bonferroni correction (p<0.008; Appendix 3-Table 5).

Ranks

	What country do you work in?	N	Mean Rank	Sum of Ranks
Asks you to repeat your speech frequently during your interviews	Europe	10	14.60	146.00
	United Kingdom	30	22.47	674.00
	Total	40		
Relatives report complaints that the volume is too high while he/she is watching TV	Europe	10	12.05	120.50
	United Kingdom	30	23.32	699.50
	Total	40		
Avoids coming to appointments because they are concerned they will not understand what you are talking about	Europe	10	15.75	157.50
	United Kingdom	30	22.08	662.50
	Total	40		

Test Statistics[a]

	Asks you to repeat your speech frequently during your interviews	Relatives report complaints that the volume is too high while he/she is watching TV	Avoids coming to appointments because they are concerned they will not understand what you are talking about
Mann-Whitney U	91.000	65.500	102.500
Wilcoxon W	146.000	120.500	157.500
Z	-2.013	-2.776	-1.542
Asymp. Sig. (2-tailed)	.044	.006	.123
Exact Sig. [2*(1-tailed Sig.)]	.067[b]	.007[b]	.140[b]

a. Grouping Variable: What country do you work in?

b. Not corrected for ties.

Appendix 3-Table 5: The comparison of the UK and Others groups in the cases of 'asking to repeat their speech frequently during their interviews', 'relatives reporting complaints that the volume is too high while he/she is watching TV' and 'avoiding coming to appointments because TBI patients are concerned they will not understand what HCPs are talking about' via the Mann-Whitney U test with Bonferroni correction

Most of participants in the UK (n=27, 39%) and Others (n=10, 14%) groups reported that the symptom of 'asking to repeat their speech frequently during their interviews' was observed in 'some' to 'a few' of their patients, most participants in Asia (n=13, 19%) reported that this symptom was observed in 'more than half' to 'some' of their patients. Most participants from Asia (n=7, 10%) and Europe (n=6, 9%) reported that 'some' of their patients' relatives complained about the volume being too high while they watch TV, whilst the majority of participants from the UK (n=15, 21%) mentioned that 'a few' of their patients' relatives reported this issue. Conversely, the majority of participants from the Others group (n=10, 14%) stated that 'a few' to 'none' of their patients' relatives reported this case. Most participants from Asia (n=6, 9%) reported that 'a few' of their patients avoided appointments because they were concerned that they would not understand what the HCPs were talking about. In contrast, most participants from the UK (n=11, 16%) reported that 'none' of their patients avoided appointments for this reason.

Another significant difference was observed between Groups 1 (physiotherapists) and 5 (doctors from other specialties) for the symptoms of 'relatives reporting complaints that the volume is too high while he/she is watching TV', 'avoiding coming to appointments because TBI patients are concerned they will not understand what HCPs are talking about', and 'an inability to tolerate certain sounds' in the Mann-Whitney U test with Bonferroni correction (p<0.003-Appendix 3-Table 6).

For peer review only

Test Statistics[a]

	Asks you to repeat your speech frequently during your interviews	Does not understand you when you are not facing her/him	Difficulty understanding you on the phone	Relatives report complaints that the volume is too high while he/she is watching TV	Avoids coming to appointments because they are concerned they will not understand what you are talking about	Reports an inability to tolerate certain sounds
Mann-Whitney U	76.500	73.000	45.000	44.500	34.000	46.000
Wilcoxon W	196.500	193.000	165.000	164.500	154.000	166.000
Z	-1.588	-1.713	-2.902	-2.949	-3.419	-2.996
Asymp. Sig. (2-tailed)	.112	.087	.004	.002	<.001	.002
Exact Sig. [2*(1-tailed Sig.)]	.137[b]	.106[b]	.004[b]	.004[b]	<.001[b]	.005[b]

a. Grouping Variable: What is your profession?

b. Not corrected for ties.

Appendix 3-Table 6: The comparison of the Groups 1and 5 in the cases of 'relatives reporting complaints that the volume is too high while he/she is watching TV', 'avoiding coming to appointments because TBI patients are concerned they will not understand what HCPs are talking about', and 'an inability to tolerate certain sounds' via the Mann-Whitney U test with Bonferroni correction

Most of the participants in Group 5 (n=8, 11%) stated that 'some' of their patients' relatives complained about the volume being too high while they watch TV, whilst the most participants of Group 1 (n=5, 7%) reported that 'none' of their patient relatives reported this problem. The majority of participants in Group 1 reported that 'none' of their TBI patients avoided coming to appointments because they were concerned, they would not understand what the HCPs were talking about. However, participants in Group 5 (n=4, 6% for each option) had equal percentages among the options of 'some', 'a few' and 'none' of their patients. The majority of participants in Group 1 (n=13, 19%) reported observing that 'some' to 'a few' of their patients reported an inability to tolerate certain sounds, whilst 17% of HCPs (n=12) in Group 5 reported observing this symptom in 'more than half' to 'some' of their patients.

For peer review only

BMJ Open

The Clinical Experiences, Current Approaches, Opinions and Awareness of Healthcare Professionals regarding the Audio-Vestibular Consequences of Individuals with Traumatic Brain Injury: A Cross-sectional Online Survey Study

Journal:	*BMJ Open*
Manuscript ID	bmjopen-2023-078017.R2
Article Type:	Original research
Date Submitted by the Author:	17-Apr-2024
Complete List of Authors:	Bölükbaş, Kübra; University of Nottingham, Hearing sciences, Mental Health and Clinical Neuroscience, School of Medicine; NIHR Nottingham Biomedical Research Centre<br>Edwards, Laura; University Hospitals of Derby and Burton NHS Foundation Trust, Division of Rehabilitation Medicine; University of Nottingham, Injury, Inflammation and Recovery Sciences, School of Medicine<br>Baguley, David; University of Nottingham, Hearing sciences, Mental Health and Clinical Neuroscience, School of Medicine; University of Nottingham, NIHR Nottingham Biomedical Research Centre<br>Fackrell, Kathryn ; University of Nottingham, Hearing sciences, Mental Health and Clinical Neuroscience, School of Medicine; NIHR Nottingham Biomedical Research Centre
**Primary Subject Heading**:	Ear, nose and throat/otolaryngology
Secondary Subject Heading:	Health services research
Keywords:	TRAUMA MANAGEMENT, Audiology < OTOLARYNGOLOGY, Surveys and Questionnaires, Patient-Centered Care, Physicians, Brain Injuries

SCHOLARONE™
Manuscripts

For peer review only - http://bmjopen.bmj.com/site/about/guidelines.xhtml

I, the Submitting Author has the right to grant and does grant on behalf of all authors of the Work (as defined in the below author licence), an exclusive licence and/or a non-exclusive licence for contributions from authors who are: i) UK Crown employees; ii) where BMJ has agreed a CC-BY licence shall apply, and/or iii) in accordance with the terms applicable for US Federal Government officers or employees acting as part of their official duties; on a worldwide, perpetual, irrevocable, royalty-free basis to BMJ Publishing Group Ltd ("BMJ") its licensees and where the relevant Journal is co-owned by BMJ to the co-owners of the Journal, to publish the Work in this journal and any other BMJ products and to exploit all rights, as set out in our licence.

The Submitting Author accepts and understands that any supply made under these terms is made by BMJ to the Submitting Author unless you are acting as an employee on behalf of your employer or a postgraduate student of an affiliated institution which is paying any applicable article publishing charge ("APC") for Open Access articles. Where the Submitting Author wishes to make the Work available on an Open Access basis (and intends to pay the relevant APC), the terms of reuse of such Open Access shall be governed by a Creative Commons licence – details of these licences and which Creative Commons licence will apply to this Work are set out in our licence referred to above.

Other than as permitted in any relevant BMJ Author's Self Archiving Policies, I confirm this Work has not been accepted for publication elsewhere, is not being considered for publication elsewhere and does not duplicate material already published. I confirm all authors consent to publication of this Work and authorise the granting of this licence.

For peer review only

The Clinical Experiences, Current Approaches, Opinions and Awareness of Healthcare Professionals regarding the Audio-Vestibular Consequences of Individuals with Traumatic Brain Injury: A Cross-sectional Online Survey Study

Kübra Bölükbaş[1,2], Laura Edwards[3,4], David M Baguley[1,2], Kathryn Fackrell[1,2,5]

[1]Hearing Sciences, Division of Mental Health and Clinical Neuroscience, School of Medicine, University of Nottingham, UK

[2]National Institute of Health and Social Research (NIHR) Nottingham Biomedical Research Centre, Nottingham, UK

[3]Division of Rehabilitation Medicine, University Hospitals of Derby and Burton NHS Foundation Trust, UK

[4]Injury, Inflammation and Recovery Sciences, School of Medicine, University of Nottingham, UK

[5]School of Healthcare Enterprise and Innovation, University of Southampton, UK

Corresponding author: Kübra Bölükbaş, NIHR Nottingham Biomedical Research Centre, Ropewalk House, 113 The Ropewalk, Nottingham, NG1 5DU, UK.

Email: kubra.bolukbas@nottingham.ac.uk

For peer review only - http://bmjopen.bmj.com/site/about/guidelines.xhtml

ABSTRACT

Objective: To explore the experiences, current approaches, opinions, and awareness of healthcare professionals (HCPs) caring for adults with Traumatic Brain Injury (TBI) regarding the audio-vestibular consequences.

Design/setting: Cross-sectional online survey study

Participants: HCPs with experience of caring for adults with TBI, who were not ENT specialists or audiologists.

Methods: The study was conducted from May 2022 to December 2022. The online survey consisted of 16 closed and open-text questions in English and Turkish about clinical experience, current approaches and awareness of audio-vestibular consequences following TBI. Frequencies of responses to closed questions and associations between variables were analysed using SPSS-v28. Open-text responses were summarised in Microsoft Excel.

Results: Seventy HCPs participated from 17 professions and 14 countries, with the majority from the UK (42.9%). HCPs stated that ‘some’ to ‘all’ of their patients had auditory problems such as ‘inability to understand speech-in-noise’ (66%), ‘tinnitus’ (64%), ‘hyperacusis’ (57%), and balance problems such as ‘dizziness’ (79%) and ‘vertigo’ (67%). Usually, HCPs asked about the balance status of patients at appointments and when they observed dizziness and/or balance disorder they used screening tests, most commonly Finger-to-Nose (53%). For auditory impairments, HCPs preferred referring TBI patients to Audiology/ENT services. However, 6% of HCPs felt that audio-vestibular conditions could be ignored upon referral because TBI patients struggled with many impairments. Additionally, 44% would suggest hearing aids to TBI patients with hearing loss ‘if they would like to use’ rather than ‘definitely’.

Conclusions: Many audio-vestibular impairments are observed by HCPs caring for TBI patients. The assessment and intervention opinions and awareness of HCPs for these impairments vary. However, non-expert HCPs may not be aware of negative consequences of untreated audio-vestibular impairments following TBI. Therefore, developing a simple framework for screening and indications of audio-vestibular impairments for referral may be helpful for non-audiological specialists regularly seeing these patients.

Keywords: Traumatic Brain Injury, Healthcare Professionals, Auditory, Vestibular, Balance, Survey

For peer review only

For peer review only - http://bmjopen.bmj.com/site/about/guidelines.xhtml

STRENGTHS AND WEAKNESSES OF THIS STUDY

1. The survey was conducted online which facilitated the participation of HCPs from diverse geographical regions worldwide.
2. The multidisciplinary approach to recruitment using established academic networks resulted in a range of respondents from different health disciplines.
3. The creation of two versions of the survey enhanced inclusivity and made it possible for respondents especially from Türkiye to participate who may not be proficient in English.
4. Despite our efforts to engage HCPs from across the world the majority of respondents were from the UK and as such findings may reflect the UK health system.

For peer review only

INTRODUCTION

Traumatic brain injury (TBI) is defined as a structural injury and/or physiological deterioration of brain function following trauma due to an external force [1]. It is estimated that 64-74 million people every year experience a TBI [2] and it is one of the leading causes of death and disability worldwide [3,4]. There are many different complications associated with TBI, such as cognitive, emotional, behavioural, and audio-vestibular impairments [5–8]. In addition, neck pain, balance disorders and tinnitus (ringing in the ear(s)) have been reported as some of the most common complaints after head and neck trauma [9,10]. Moreover, in a review investigating hearing loss in TBI without skull fracture, Chen et al. [11] found that 58% of TBI patients experienced hearing loss, sometimes temporarily, despite not having any skull fractures. This rate suggests that the percentage of audio-vestibular consequences related to TBI that are not reported and/or investigated worldwide may be much higher than expected.

Consequences of TBI vary greatly by nature, severity and duration. Some individuals may be left with minimal impairment and make a rapid recovery with/without specialist intervention, whilst others may require inpatient or outpatient neurorehabilitation [1]. This diversity of complications due to TBI necessitates a multidisciplinary approach. Accordingly, depending on the severity and nature of the complication, a patient with TBI may be treated by a wide range of healthcare professionals (HCPs) including physiotherapists, psychologists, occupational therapists. The emergence of hearing loss and/or vestibular disorders, tinnitus, or hyperacusis (sound sensitivity) following TBI further complicates the treatment of TBI and requires a sensitive and careful approach in the diagnosis and treatment of audio-vestibular disorders in this patient group. Untreated hearing loss for example can lead to additional conditions such as dementia [12] as well as many psychological problems such as communication difficulties and social isolation [13,14]. Therefore, assessment and treatment of audio-vestibular conditions should not be neglected when caring for adults with TBI as early detection of audio-vestibular impairments in patients after TBI can improve quality-of-life, minimise complications and prevent additional conditions such as dementia.

Patients struggling with many problems such as physical, cognitive, emotional and behavioural complications after TBI may not easily notice any audio-vestibular impairments immediately. However, HCPs who care for them should understand and assess their audio-vestibular conditions, even if it is not their field of expertise and, if necessary, arrange referrals to the ENT (ear nose throat) specialists or audiologists. A recent survey on audio-vestibular clinical

For peer review only - http://bmjopen.bmj.com/site/about/guidelines.xhtml

practice within Australian audiology departments found gaps in audiologist knowledge and inadequate resources and practice regarding the identification, diagnosis and management of patients with auditory and/or vestibular deficits following TBI [15]. However, as yet, the clinical experiences, current approaches, opinions and awareness of HCPs who specialise in managing patients with TBI have not been fully investigated. It is therefore important to understand the experiences of these HCPs in particular to understand their awareness and knowledge of the audio-vestibular consequences of TBIs, in order to understand and identify areas that could be improved in existing practices.

Accordingly, the aim of this study was to explore and gain an understanding of the clinical experiences, current approaches, opinions, and awareness of the HCPs caring for adults with TBI (except ENT specialists and audiologists) regarding the audio-vestibular consequences experienced by these patients.

METHODS

Design

This study used a cross-sectional international online survey to explore the clinical experiences, current approaches, opinions and awareness of HCPs regarding the audio-vestibular conditions experienced by patients who had a TBI. Research approval was obtained from the University of Nottingham, Faculty of Medicine and Health Sciences Research Ethics Committee (FMHS 462-0222, May 2022). Before initiating the survey questions, on the first page, an information page was provided detailing who the researchers are, the purpose of the survey, its duration, the advantages and disadvantages of participation, who would have access to the survey data, where and for how long the data would be stored. Following the information page, all participants were requested to give informed consent. This survey study is reported according to Checklist for Reporting Results of Internet E-Surveys (CHERRIES) [16].

Survey Development

The survey questions were iteratively developed and refined through discussion and consensus within the study team. The authors initially compiled a list of potential questions and relevant sections for inclusion in the survey. Questions were based on experience of the authors and evidence from research. To ensure the questions and response options were relevant and valid for HCPs, the surveys were piloted with two HCPs and one researcher who have experience of

For peer review only - http://bmjopen.bmj.com/site/about/guidelines.xhtml

audio-vestibular assessments. The questions were revised and distilled down to create a 16-question survey (over 10 pages, with a maximum of 3 questions on per page) which included closed questions with single-choice, multiple-choice options, or Likert scales and for 4 questions with a free-text "other" option. Sub-question (16a) of the last question used adaptive questioning, which was only displayed conditionally based on 'yes' responses to question 16. Respondents were asked to consider patients with TBIs that occurred more than 6 months ago and the reported or observed audio-vestibular symptoms at that time. It was necessary to answer each question and to complete the survey in one session. It was possible for respondents to review and edit their responses until the point they chose to submit the survey on the final page. In order to reach a range of HCPs, and since the lead author (KB) is bilingual, two versions of the survey were created: one in English and one in Turkish (Supplemental appendix 1). The survey was delivered online using JISC software maintained by the University of Nottingham (https://www.onlinesurveys.ac.uk/) which collected and stored responses from each respondent. Respondents accessed the open survey via a link (passwords were not provided).

Patient and Public Involvement

No patients were directly involved in the development, dissemination or reporting of this study. The study focused on the experiences of HCPs in the field of TBI, such as physiotherapists, neuropsychologists, rehabilitation nurses, therefore the survey was piloted with HCPs and a researcher who have experience of audio-vestibular assessments to gain their views and refine questions, and two of the authors are clinicians with relevant experience in the field.

Study Sample and Eligibility

The study population was identified as HCPs caring for adult patients with TBI worldwide. To be eligible to participate in the survey, respondents were required to be HCPs with clinical experience in caring for adult patients with TBI and to have the ability to understand and answer questions in English (except HCPs in Türkiye). ENT specialists and audiologists were excluded from the study as they were already experts in the field of audio-vestibular care.

Distribution

A number of recruitment strategies were used to ensure a broad, representative sample of HCPs with a range of experiences, current approaches, opinions and awareness regarding audio-vestibular conditions in TBI patients. The survey invitation and link were circulated via

For peer review only - http://bmjopen.bmj.com/site/about/guidelines.xhtml

professional organisations' contact lists including Headway UK, British Society of Physical and Rehabilitation Medicine, and Royal College of Speech and Language Therapists (see Supplemental appendix 2 for full list), promotion in blogs (e.g., Krysalis Neuro Occupational Therapy), and through social media channels (Facebook, Twitter, and Instagram). In addition, an invitation email with the survey link was sent to the HCPs identified via Expertscape website (https://expertscape.com/) as experts in the field of TBI. All of these distribution channels were selected taking into account the inclusion criteria of our study. Participants were not given a specific timeframe to complete the survey. Responses were obtained between May 2022 and December 2022. Participation in the survey was voluntarily and no incentives were provided.

Data analysis

All survey data were imported to Microsoft Excel 2016©. Prior to analysis, data were checked to ensure all respondents fully consented and responses were given to all questions as required. If any surveys were terminated early, they were not visible because they were not submitted. Only completed surveys were analysed. Descriptive statistics, in particular frequencies and percentages of responses to closed questions were calculated in SPSS (v28). Open-text responses (short sentences or lists) were reviewed and categorized in Microsoft Excel 2016©. In particular, similar responses were categorized together and then summarised.

Occupations, countries of residence, years of occupation, years of working with TBI patients, and hours per week working with TBI adults were analysed using non-parametric statistical methods to assess relations between these categorical variables. To ensure a meaningful statistical analysis in comparisons, countries of residence were divided into four groups (the UK, Europe, Asia and Others) and occupations into six groups as seen in Table 1 (Group 1 representing physiotherapists; Group 2 occupational therapists; Group 3 speech and language therapists; Group 4 physiatrist or rehabilitation medicine doctors and rehabilitation nurses; Group 5 doctors from other specialties, and Group 6 'other') due to the imbalance in participant numbers. The Kruskal-Wallis test was utilized for comparing more than two independent groups in each category. Following this measurement, to identify the groups that contribute to the observed differences in the data, pairwise subgroup comparisons were conducted using Mann-Whitney U test with Bonferroni's correction for country of residence groups: $p<0.05/(4*3/2)$, for occupation groups: $p<0.05/(6*5/2)$, for years working with TBI adults groups: $p<0.05/(4*3/2)$.

For peer review only

For peer review only - http://bmjopen.bmj.com/site/about/guidelines.xhtml

RESULTS

Characteristics of participants

The characteristics of respondents are reported in Table 1. A total of 70 HCPs completed the survey from 14 different countries. Of these, the majority were from the UK (n=30, 42.9%), followed by Türkiye (n=14, 20%) and New Zealand (n=8, 11.5%). Respondents reported a range of occupations, including physiotherapists (n=15, 21.4%), occupational therapists (n=11, 15.8%) and speech and language therapists (n=10, 14.2%). The majority (48/70, 69%) had been in their occupation for more than 10 years and over half (39/70, 56%) had been working with TBI patients between 5 and 20 years. Over half of the respondents (57%) spent more than 10 hours per week working with adult patients with TBI. There were no statistically significant differences in years of occupation and years working with TBI adults among participants based on groups of country of residence and occupations ($p>0.05$). However, statistically significant differences were observed in the working hours per week with TBI adults based on groups of country of residence and occupations in the Kruskal-Wallis test ($p<0.05$). Subgroup statistical analyses showed that participants from the UK (n=16, 23%) were more likely to spend more than 20 hours a week with TBI adults, but those from Asia (n=7, 10%) were more likely to spend less than 1 hour per week ($p<0.008$). Participants in Group 2 (occupational therapists; n=6, 9%) were reported to spend over 20 hours per week with TBI adults, whilst participants in Group 5 (doctors from other specialties; n=5, 7%) spent less than 1 hour per week with TBI adults ($p<0.003$). Exactly half of the respondents worked in private healthcare (35/70, 50%), and the other half worked in National Health Service (NHS) or state-owned hospitals.

Table 1. Demographic information of participants

Characteristics	n	%	Statistical analysis groups
	**70**	**100**	
**Country of residence**			
United Kingdom	30	42.9%	UK
Türkiye	14	20.0%	Asia
New Zealand	8	11.5%	Others
Ireland	6	8.6%	Europe
Canada	2	2.9%	Others
Australia	2	2.9%	Others
Greece	1	1.4%	Europe

For peer review only - http://bmjopen.bmj.com/site/about/guidelines.xhtml

United States of America	1	1.4%	Others
Azerbaijan	1	1.4%	Asia
Italy	1	1.4%	Europe
Spain	1	1.4%	Europe
Croatia	1	1.4%	Europe
Venezuela	1	1.4%	Others
Tanzania	1	1.4%	Others
**Occupation**			
Physiotherapist	15	21.4%	Group 1
Occupational Therapist	11	15.8%	Group 2
Speech and Language Therapist	10	14.2%	Group 3
Physiatrist or Rehabilitation Medicine Doctor	6	8.6%	Group 4
Neuropsychiatrist	5	7.1%	Group 5
Neuropsychologist	4	5.8%	Group 6
Rehabilitation Nurse	4	5.8%	Group 4
Neurologist	3	4.2%	Group 5
Psychologist	2	2.9%	Group 6
Geriatrician	2	2.9%	Group 5
Neurosurgeon	2	2.9%	Group 5
Optometrist	1	1.4%	Group 6
Case Manager	1	1.4%	Group 6
Anaesthetist	1	1.4%	Group 5
Behavioural Scientist	1	1.4%	Group 6
Primary Care Doctor	1	1.4%	Group 5
Orthopaedic Surgeon	1	1.4%	Group 5
**Years in occupation**			
Less than 5 years	8	11%	Less than 5 years
5 – 10 years	14	20%	5 – 10 years
10 – 20 years	25	36%	10 – 20 years
More than 20 years	23	33%	More than 20 years
**Years working with TBI adults**			
Less than 5 years	16	23%	Less than 5 years
5 – 10 years	20	29%	5 – 10 years
10 – 20 years	19	27%	10 – 20 years
More than 20 years	15	21%	More than 20 years
**Hours per week working with TBI adults**			
Less than an hour	7	10%	Less than an hour

For peer review only - http://bmjopen.bmj.com/site/about/guidelines.xhtml

1 – 5 hours	13	19%	1 – 5 hours
5 – 10 hours	10	14%	5 – 10 hours
10 – 20 hours	15	21%	10 – 20 hours
More than 20 hours	25	36%	More than 20 hours

The clinical experiences of HCPs regarding audio-vestibular consequences in TBI patients

This section explores the reported and/or observed audio-vestibular symptoms in adult patients with TBI. According to the results, the majority of responses ranged across 'a few' to 'most of my patients' describe the symptom (Figure 1). In general, HCPs reported that 'some' to 'all' of their patients had auditory problems such as 'inability to understand speech-in-noise' (46/70, 66%), 'tinnitus' (45/70, 64%), 'hyperacusis' (40/70, 57%), and 'inability to capture conversations' (44/70, 63%) and balance problems such as 'dizziness' (55/70, 79%), and 'vertigo' (47/70, 67%). However, the majority of HCPs observed that 'none' to 'a few' patients reported problems with 'inability to walk in the dark without a physical problem' (31/70, 44%), 'inability to understand speech in quiet' (40/70, 57%) and 'hearing loss' (41/70, 58%). According to the Kruskal-Wallis test, no significant differences were found in the frequency of audio-vestibular symptoms reported by patients to HCPs, based on years of working with TBI adults and groups of country of residence ($p>0.05$). Statistically significant differences were observed for symptom of 'inability to capture conversations' based on groups of occupations ($p<0.05$). According to the Mann-Whitney U test with Bonferroni correction, a statistically significant difference was detected between Group 3 (speech and language therapists) and Group 1 (physiotherapists) for the symptom of 'inability to capture conversations' ($p<0.003$). Most speech and language therapists (n=7, 10%) reported that 'most' of their patients described 'inability to capture conversations', whilst most physiotherapists (n=10, 14%) reported that only 'a few' of their patients reported this symptom.

*** Insert Figure 1 here ***

The observed audio-vestibular symptoms by HCPs in patients with TBI (where trauma occurred more than 6 months ago) or reported to HCPs by patients' relatives are shown in Figure 2. HCPs observed that 'some' to 'all' of their patients 'asked to repeat their speech frequently during their interviews' (45/70, 64%), 'difficulty understanding them on the phone'

For peer review only - http://bmjopen.bmj.com/site/about/guidelines.xhtml

(38/70, 54%), and 'an inability to tolerate certain sounds' (44/70, 63%). In contrast, 37% of HCPs (26/70) did state that 'none' of their patients 'avoided coming to appointments because they were concerned, they would not understand what HCPs were talking about'. However, 17% (12/70) did not know whether there was such a concern for their patients. The details of the statistical analysis results regarding the associations between audio-vestibular symptoms observed by HCPs or reported by patients' relatives to HCPs, and the years working with TBI adults and groups of country of residence, are explained in Supplemental Appendix 3.

Statistically significant differences were observed in the Kruskal-Wallis test between groups of occupations regarding the symptoms of 'asking to repeat their speech frequently during their interviews', 'not understanding HCPs when they are not facing with them', 'difficulty understanding HCPs on the phone' ($p<0.05$). Subgroup statistical analyses demonstrated significant differences in communication-related symptoms between Group 1 (physiotherapists) and Group 3 (speech and language therapists) ($p<0.003$). Based on this, most speech and language therapists (n=7, 10%) reported that 'more than half' of their patients asked to repeat their speech frequently during interviews, whilst the majority of physiotherapists (n=12, 17%) indicated that 'some' to 'a few' of their patients asked to repeat their speech frequently. Additionally, most speech and language therapists (n=6, 9%) stated that 'some' of their patients did not understand them when they were not facing them, similar to physiotherapists where the same percentage (n=6, 9%) reported observing this problem in 'a few' of their patients. The majority of speech and language therapists (n=6, 9%) reported that 'some' of their patients had difficulty understanding them on the phone, whilst physiotherapists (n=4, 6% for each option) had an equal percentage distribution among the options 'some', 'a few' of their patients, and 'do not know'. The statistical differences in other symptoms observed among other groups of occupations are detailed in Supplemental Appendix 3.

Over 40% (30/70) of HCPs were unable to identify and generalise the aetiology of the TBI patients who frequently reported audio-vestibular complaints. Of the 21% (15/70) that were able, most reported that likely aetiologies were vehicle-related collisions (9/15, 60%), followed by falls (2/15, 13%) and sports injuries (1/15, 7%). Those who selected 'other' trauma option (3/15, 20%) indicated that audio-vestibular complaints could result from any TBI aetiology, including an object falling on the head, vehicle related collisions and/or sports related injuries.

*** Insert Figure 2 here ***

For peer review only - http://bmjopen.bmj.com/site/about/guidelines.xhtml

The current approaches to assess and manage audio-vestibular conditions of TBI patients

To understand the current approaches used by HCPs regarding the audio-vestibular conditions of their TBI patients, we asked questions about screening and assessment methods. Over half of respondents (52/70, 74%) indicated that they ask their TBI patients about their balance status (e.g., dizziness, vertigo), whilst the remaining stated that they only asked if their balance status was interfering with the conduct of the appointment (10/70, 14%), they did not ask if there are no complaints (5/70, 7%), or they preferred not to ask because it was not their expertise (1/70, 1%). When they noticed dizziness or balance disorder in TBI patients, the most commonly used screening methods were finger-to-nose (37/70, 53%), gait analysis (34/70, 49%), and eye movements (34/70, 49%) as seen in Figure 3. In addition to these tests, HCPs also reported using evaluation methods such as videonystagmography (VNG) and dynamic visual acuity in open-text responses. Again, several HCPs reported that they prefer not to use any screening method because it was not their area of expertise (23/70, 33%). In relation to checking the external ear condition of TBI patients, 13% (9/70) of HCPs, in particular physiatrists or rehabilitation medicine doctors reported that they evaluated 'the external ear' by themself with an otoscope. However, almost half of the respondents (34/70, 49%) stated that they would refer patients to the ENT and/or audiology department to check the external ear conditions.

*** Insert Figure 3 here ***

When asked how many TBI patients they overall referred to ENT and/or audiology, 78% (55/70) of HCPs said that they referred 'some' or 'a few' of their TBI patients. Only 4% (3/70) of HCPs referred 'all' of their TBI patients to ENT and/or audiology department. In the questions asked to understand the opinions and awareness of HCPs, HCPs stated that they considered referring TBI patients to the ENT specialist and/or audiology department, when their patients could not understand and/or hear their speech (37/70, 53%), when their patients reported tinnitus and hyperacusis (33/70, 47%) or when they observed vertigo and/or dizziness in their patients (29/70, 41%). Only 6% (4/70) felt that audio-vestibular conditions could be ignored because TBI patients struggled with many impairments (Figure 4). In contrast, when asked whether TBI patients should use a hearing aid if they have hearing loss, no one thought it was something that could be ignored. However, most of the respondents (31/70, 44%) stated that TBI patients with hearing loss should use hearing aids 'if they would like to', rather than 'definitely', whilst the remaining did not know (14/70, 20%). Among the participants who indicated not knowing the majority were physiotherapists (n=8, 11%). When asked if they could help their TBI patients using hearing aids at their appointments, interestingly whilst 40%

For peer review only - http://bmjopen.bmj.com/site/about/guidelines.xhtml

(28/70) stated that they did not know anything about hearing aids, 40% (28/70) also stated that they could change the battery of the hearing aid. The majority (32/70, 46%), however, did state that they would notice whether the hearing aid was working. Notably, speech and language therapists were not among the HCPs who reported not knowing anything about hearing aids.

*** Insert Figure 4 here ***

DISCUSSION

This study explored the clinical experiences, current approaches, opinions, and awareness of HCPs of audio-vestibular consequences in TBI patients. In general, the majority of HCPs had observed audio-vestibular impairments in their TBI patients in line with the literature [17–19]. However, most HCPs reported that hearing loss was only observed in 'a few' of patients. In contrast to this finding, a systematic review study which investigated peripheral auditory dysfunction secondary to TBI showed that sensorineural hearing loss was the most frequently observed auditory impairment at a rate of 37.3% in patients with non-blast related TBI [17]. Additionally, Knoll et al. [20] showed that following TBI, individuals can experience auditory symptoms even if their hearing thresholds are within the normal range (≤25 dB HL). Therefore, these studies suggest that hearing loss can be observed or reported more frequently by TBI patients than HCPs may observe. One potential explanation for these differences could be that the HCPs who participated in this study are not experts in audio-vestibular assessments and as such may not be looking for audiological impairments, particularly during appointments which may be addressing other concerns. However, recognition of hearing loss in TBI patients by non-audiological-specialist HCPs may result in more onwards referrals for detailed assessment and input, resulting in better outcomes.

We also observed that HCPs were more likely to assess their TBI patients' vestibular and/or balance-related conditions than their hearing-related conditions. One explanation for this is that different HCPs such as neurologists, physiotherapists and occupational therapists, who participated in this study, may be more likely to assess and treat balance and/or vestibular function, and less likely to be familiar with auditory assessments. Another explanation could be that these professional groups prioritize physical function. These factors might be a possible reason why patients with auditory problems were more likely to be referred. Additionally, the observation of communication-related symptoms more likely in individuals with TBI by speech and language therapists rather than physiotherapists can be associated with their field

For peer review only - http://bmjopen.bmj.com/site/about/guidelines.xhtml

of expertise in communication. These findings may also contribute to emphasizing the importance of differences in patient care among professional groups and a multidisciplinary approach.

However, in contrast, a number of HCPs did not refer to ENT or audiology services if the patient had not complained about auditory problems and/or if they felt that the audio-vestibular problems were an issue that were not a priority compared to other TBI-related impairments. This suggests that some HCPs may not be aware of the negative short-term and long-term consequences of untreated audio-vestibular impairments. Many HCPs reported that they would leave the use of a hearing aid to the discretion of individual patients. This is understandable, in that patients who do not want to use a hearing aid are unlikely to use one, even if it is provided. However, it may also reflect lack of training and education in hearing aids for HCPs. It is important that HCPs explore the patient's rationale and capacity [21] for making this decision as well as explaining the potential consequences of untreated hearing loss [12].

Finally, consistent with the studies in the literature [15,22], several HCPs reported that TBI patients with audio-vestibular complaints had the most vehicle-related collision aetiology. However, this was not universal and likely reflects the fact that audio-vestibular complaints are a common consequence of a wide variety of TBI aetiologies.

Interestingly, a recent Australian study [15] also showed that audiologists lacked knowledge about the assessment and management of audio-vestibular impairments of TBI patients in audiological services. Whilst there can be variation in understanding of audio-vestibular consequences of TBI across clinical services and/or centres around the world, this study demonstrated a similar lack of awareness regarding the audio-vestibular consequences of TBI patients in non-audiology HCPs. This highlights the evident need for greater awareness among HCPs working with this patient population, even if it is outside their area of expertise. For this purpose, it may be beneficial to prepare a practical audiology guide for all HCPs dealing with TBI in future studies.

Strengths and Limitations

To our knowledge, this is the first study to investigate the clinical experiences, current approaches, opinions and awareness of HCPs, other than ENT specialists and audiologists, regarding the audio-vestibular consequences of TBI patients. This international survey received responses from a total of 70 participants from 17 different departments and 14 countries and as such we have gained knowledge from a range of HCPs who are actively

For peer review only - http://bmjopen.bmj.com/site/about/guidelines.xhtml

working in clinical settings that assess and manage audio-vestibular consequences in TBI patients. However, we do recognise that the majority were from the UK and that there are low to no participation rates for some countries. This means that some of the findings may reflect the UK health system above others. For example, other countries may have audiologists/ENTs as part of their multi-disciplinary team. Open online surveys may have inherent issues such as the possibility of participants submitting multiple responses and leading to response fraud in recruitment through particularly social media. However, anonymity with this method allows respondents to provide honest and candid answers freely [23]. Although no tracking method was used to address these issues in our study, the shared social media accounts and links included individuals from the academic community and HCPs. In addition, responses were checked for inconsistencies or evidence of deception through data cleaning. Furthermore, the inclusion of HCPs such as physiotherapists working in the field of balance may have influenced the results, in particular the confidence in vestibular assessments. Finally, we wanted to understand the experience of HCPs who specialise in TBI therefore we did not include audiologists and/or ENT specialists in this survey and as such we do not know their experiences with this patient population, except for the findings from the Australia survey [15] which indicate similar experiences to our findings here. Future research should consider further investigation into the experiences of audiologists/ENT specialists in relation to this patient population, in particular the assessments that should be carried out.

Conclusion

HCPs who care for individuals who sustained TBI observe a wide range of audio-vestibular outcomes such as inability to understand speech-in-noise, tinnitus, hyperacusis, dizziness and vertigo in these patients. HCPs' current approaches, opinions and awareness around further assessment, investigation and treatment were variable in this study. HCPs may be more likely to assess vestibular and/or balance-related conditions than their hearing-related conditions in this patient group. In general, HCPs were referring only some or a few of their TBI patients to ENT and/or audiology department which may negatively impact the potential diagnosis of audio-vestibular (especially auditory) impairments in TBI patients. Untreated audio-vestibular impairments may have significant adverse consequences for patients and non-expert clinicians may be unaware of these. We suggest that standardised screening protocols may enable easier identification of audio-vestibular problems with signposting to appropriate specialties for further assessment and intervention. Thus, audio-vestibular impairments of TBI patients can be diagnosed and treated earlier, to improve quality-of-life of TBI patients.

For peer review only - http://bmjopen.bmj.com/site/about/guidelines.xhtml

ACKNOWLEDGEMENTS

The authors wish to acknowledge the clinicians who piloted the survey and all the respondents of the survey. We would like to express our gratitude to Professor David Baguley, who sadly passed away during this study. His knowledge and expertise helped develop the fundamental ideas for this research, for which we are very thankful.

AUTHOR STATEMENT

DB, LE, KB authors conceptualised the project and design of the survey questions. KB led the recruitment and data collection, supported by KF, DB, LE. KF, DB and LE informed methodology and interpretation of analysis. KB conducted initial analysis. KF and LE reviewed and refined analysis. KB drafted manuscript. KF, LE and KB reviewed and approved the final manuscript for submission.

FUNDING

This work was supported by the Ministry of National Education of the Republic of Türkiye and the National Institute for Health Research (NIHR) Nottingham Biomedical Research Centre (N/A for the award/grant number).

DISCLAIMER

KB was funded by the Ministry of National Education of the Republic of Türkiye to undertake this work as part of her PhD (N/A for the award/grant number). KF was funded by National Institute for Health Research (NIHR Post-Doctoral Fellowship, PDF-2018-11-ST2-003) at the time of completing this work. The views expressed in this publication are those of the author(s) and not necessarily those of the NIHR, the NHS or the Department of Health and Social Care.

COMPETING INTERESTS

None declared

PATIENT CONSENT FOR PUBLICATION

Not required

For peer review only

For peer review only - http://bmjopen.bmj.com/site/about/guidelines.xhtml

ETHICS APPROVAL

Research approval was obtained from the University of Nottingham, Faculty of Medicine and Health Sciences Research Ethics Committee (FMHS 462-0222, May 2022). All respondents gave informed consent at the beginning of the survey.

PROVENANCE AND PEER REVIEW

Not commissioned; externally peer reviewed.

DATA SHARING STATEMENT

Data are available upon reasonable request.

ORCID IDS

Kübra Bölükbaş https://orcid.org/0000-0002-7625-4758

Laura Edwards https://orcid.org/0000-0003-1392-2648

Kathryn Fackrell http://orcid.org/0000-0001-6529-8643

TWITTER

Kubra Bolukbas @_kubrabolukbas_, Kathryn Fackrell @FackrellKathryn

For peer review only - http://bmjopen.bmj.com/site/about/guidelines.xhtml

REFERENCES

1. NICE. Head injury briefing paper. 2014 [cited 2023 Jan 2];77. Available from: https://www.nice.org.uk/guidance/qs74/documents/head-injurybriefing- paper2.

2. Dewan MC, Rattani A, Gupta S, Baticulon RE, Hung YC, Punchak M, et al. Estimating the global incidence of traumatic brain injury. J Neurosurg. 2019 Apr 1;130(4):1080–97.

3. Maas AIR, Menon DK, Steyerberg EW, Citerio G, Lecky F, Manley GT, et al. Collaborative European NeuroTrauma Effectiveness Research in Traumatic Brain Injury (CENTER-TBI): A Prospective Longitudinal Observational Study. Neurosurgery [Internet]. 2015;76(1). Available from: https://journals.lww.com/neurosurgery/Fulltext/2015/01000/Collaborative_European_NeuroTrauma_Effectiveness.8.aspx

4. Rubiano AM, Carney N, Chesnut R, Puyana JC. Global neurotrauma research challenges and opportunities. Vol. 527, Nature. Nature Publishing Group; 2015. p. S193–7.

5. Ahmed S, Venigalla H, Mekala HM, Dar S, Hassan M, Ayub S. Traumatic brain injury and neuropsychiatric complications. Vol. 39, Indian Journal of Psychological Medicine. Medknow Publications; 2017. p. 114–21.

6. Konrad C, Geburek AJ, Rist F, Blumenroth H, Fischer B, Husstedt I, et al. Long-term cognitive and emotional consequences of mild traumatic brain injury. Psychol Med. 2011 Jun;41(6):1197–211.

7. Hoover EC, Souza PE, Gallun FJ. Auditory and cognitive factors associated with speech-in-noise complaints following mild traumatic brain injury. In: Journal of the American Academy of Audiology. American Academy of Audiology; 2017. p. 325–39.

8. Campbell KR, Parrington L, Peterka RJ, Martini DN, Hullar TE, Horak FB, et al. Exploring persistent complaints of imbalance after mTBI: Oculomotor, peripheral vestibular and central sensory integration function. Journal of Vestibular Research. 2021;31:519–30.

For peer review only

For peer review only - http://bmjopen.bmj.com/site/about/guidelines.xhtml

9. Kreuzer PM, Landgrebe M, Schecklmann M, Staudinger S, Langguth B. Trauma-Associated Tinnitus: Audiological, Demographic and Clinical Characteristics. PLoS One [Internet]. 2012 Sep 26;7(9):e45599-. Available from: https://doi.org/10.1371/journal.pone.0045599

10. Elzière M, Devèze A, Bartoli C, Levy G. Post-traumatic balance disorder. Vol. 134, European Annals of Otorhinolaryngology, Head and Neck Diseases. Elsevier Masson SAS; 2017. p. 171–5.

11. Chen JX, Lindeborg M, Herman SD, Ishai R, Knoll RM, Remenschneider A, et al. Systematic review of hearing loss after traumatic brain injury without associated temporal bone fracture. Am J Otolaryngol. 2018 May 1;39(3):338–44.

12. Livingston G, Huntley J, Sommerlad A, Ames D, Ballard C, Banerjee S, et al. Dementia prevention, intervention, and care: 2020 report of the Lancet Commission. The Lancet [Internet]. 2020 Aug 8;396(10248):413–46. Available from: https://doi.org/10.1016/S0140-6736(20)30367-6

13. Wilson BS, Tucci DL. Addressing the global burden of hearing loss. The Lancet [Internet]. 2021 Mar 13;397(10278):945–7. Available from: https://doi.org/10.1016/S0140-6736(21)00522-5

14. Monzani D, Galeazzi GM, Genovese E, Marrara A, Martini A. Psychological profile and social behaviour of working adults with mild or moderate hearing loss. Acta Otorhinolaryngologica Italica. 2008;28(2):61–6.

15. Šarkić B, Douglas JM, Simpson A. A cross-sectional survey of non-specialist Australian audio-vestibular clinical practice for traumatic brain injury and rehabilitation. Brain Impairment [Internet]. 2023 Jan 6 [cited 2023 Jan 13];1–18. Available from: https://www.cambridge.org/core/product/identifier/S1443964622000353/type/journal_article

16. Eysenbach G. Improving the quality of web surveys: The Checklist for Reporting Results of Internet E-Surveys (CHERRIES). Vol. 6, Journal of Medical Internet Research. JMIR Publications Inc.; 2004.

For peer review only - http://bmjopen.bmj.com/site/about/guidelines.xhtml

17. Šarkić B, Douglas JM, Simpson A. Peripheral auditory dysfunction secondary to traumatic brain injury: a systematic review of literature. Brain Inj [Internet]. 2019 Jan 28;33(2):111–28. Available from: https://doi.org/10.1080/02699052.2018.1539868

18. Roup CM, Ross C, Whitelaw G. Hearing difficulties as a result of traumatic brain injury. J Am Acad Audiol. 2020;31(2):137–46.

19. Šarkić B, Douglas JM, Simpson A, Vasconcelos A, Scott BR, Melitsis LM, et al. Frequency of peripheral vestibular pathology following traumatic brain injury: a systematic review of literature. Vol. 60, International Journal of Audiology. Taylor and Francis Ltd.; 2020. p. 479–94.

20. Knoll RM, Lubner RJ, Brodsky JR, Wong K, Jung DH, Remenschneider AK, et al. Auditory Quality-of-Life Measures in Patients With Traumatic Brain Injury and Normal Pure Tone Audiometry. Otolaryngology - Head and Neck Surgery (United States). 2020 Dec 1;163(6):1250–4.

21. Cotrena C, Branco LD, Zimmermann N, Cardoso CO, Grassi-Oliveira R, Fonseca RP. Impaired decision-making after traumatic brain injury: The Iowa Gambling Task. Brain Inj [Internet]. 2014 Jul 1;28(8):1070–5. Available from: https://doi.org/10.3109/02699052.2014.896943

22. Peeters W, van den Brande R, Polinder S, Brazinova A, Steyerberg EW, Lingsma HF, et al. Epidemiology of traumatic brain injury in Europe. Acta Neurochir (Wien) [Internet]. 2015;157(10):1683–96. Available from: https://doi.org/10.1007/s00701-015-2512-7

23. Teitcher JEF, Bockting WO, Bauermeister JA, Hoefer CJ, Miner MH, Klitzman RL. Detecting, Preventing, and Responding to "Fraudsters" in Internet Research: Ethics and Tradeoffs. The Journal of Law, Medicine & Ethics [Internet]. 2015 Apr 1;43(1):116–33. Available from: https://doi.org/10.1111/jlme.12200

FIGURE LEGENDS

Figure 1. The frequency of audio-vestibular symptoms reported by patients to HCPs.

For peer review only

For peer review only - http://bmjopen.bmj.com/site/about/guidelines.xhtml

Figure 2. The frequency of audio-vestibular symptoms reported by patients or their relatives to HCPs and/or observed by HCPs.

Figure 3. Percent of screening methods used by HCPs when they notice dizziness or balance disorders in patients with TBI.

Figure 4. Percent of reasons for HCPs to refer TBI patients to the ENT and/or audiology department.

SUPPLEMENTAL MATERIALS

Supplemental Appendix 1. English and Turkish versions of the survey

Supplemental Appendix 2. List of organisations that were contacted to share the surveys

Supplemental Appendix 3. The results of statistical analysis on associations between audio-vestibular symptoms observed by HCPs or reported by patients' relatives to HCPs, and the years working with TBI adults, groups of country of residence and occupations

For peer review only

For peer review only - http://bmjopen.bmj.com/site/about/guidelines.xhtml

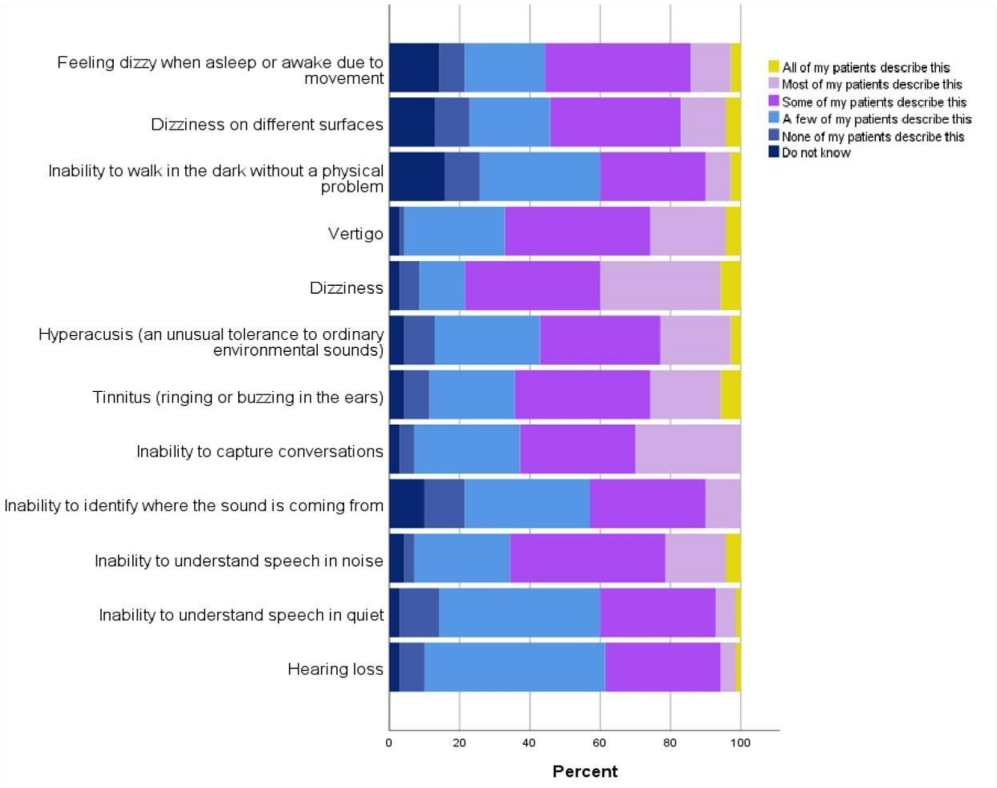

Figure 1. The frequency of audio-vestibular symptoms reported by patients to HCPs

52x41mm (600 x 600 DPI)

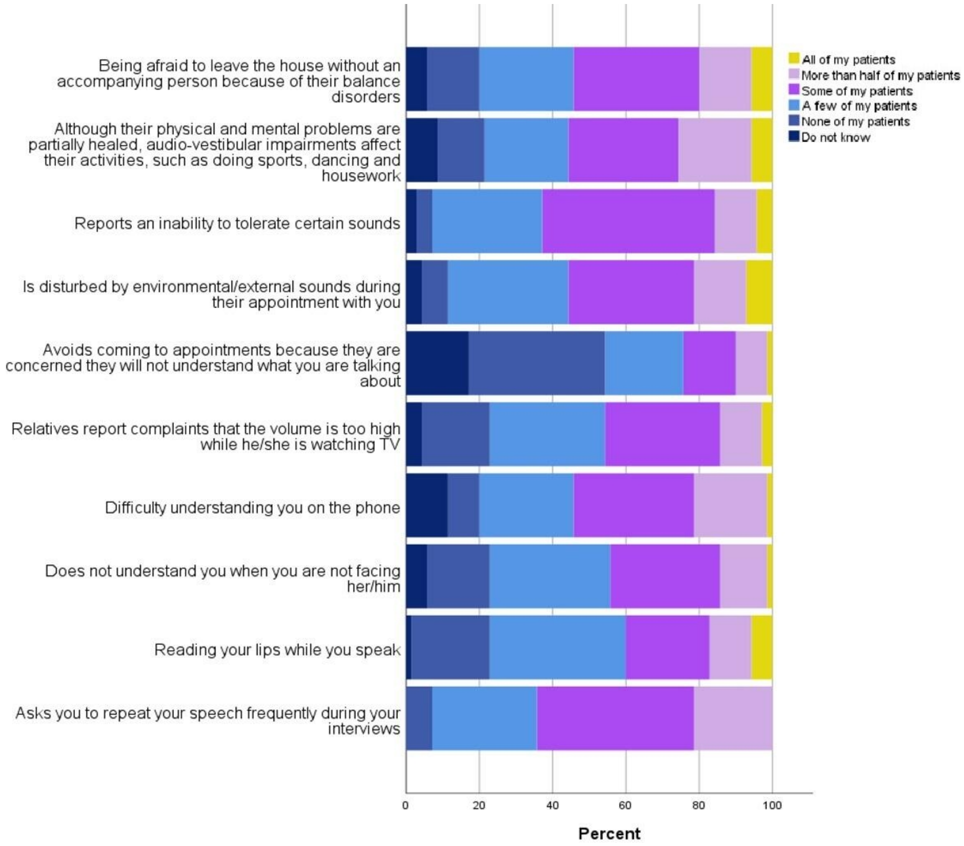

Figure 2. The frequency of audio-vestibular symptoms reported by patients or their relatives to HCPs and/or observed by HCPs

51x43mm (600 x 600 DPI)

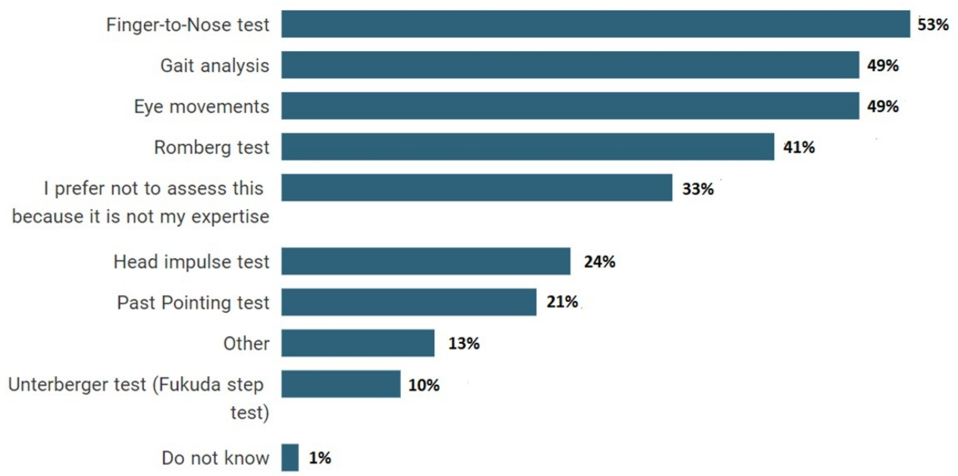

Figure 3. Percent of screening methods used by HCPs when they notice dizziness or balance disorders in patients with TBI

50x24mm (600 x 600 DPI)

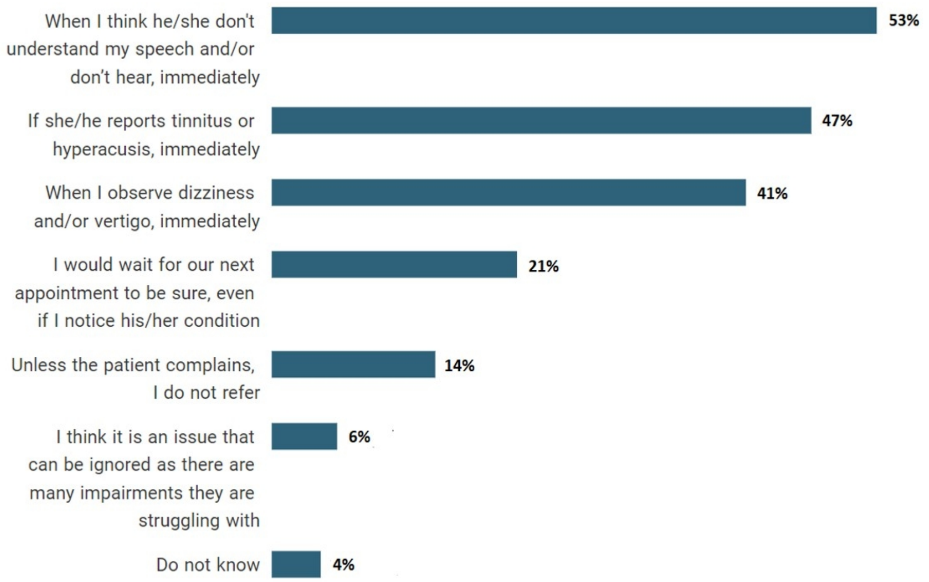

Figure 4. Percent of reasons for HCPs to refer TBI patients to the ENT and/or audiology department.

50x30mm (600 x 600 DPI)

Supplemental Appendix 1. English and Turkish version of the survey

English Version of Survey

Healthcare professionals and Audio- vestibular consequences of Traumatic Brain Injury

Page 1: Information and Consent Page for an OnlineSurvey/Questionnaire

Information and Consent page for an Online Survey/Questionnaire

Study Title: Experiences and opinions of healthcare professionals about the audio-vestibular findings of individuals with traumatic brain injury

Research Team:

Name of supervisors:

Kathryn Fackrell, Senior Research Fellow

Laura Edwards, Clinical Associate Professor in the Faculty of Medicine and Health Sciences

Co-investigator:

Kubra Bolukbas, PhD in Hearing Sciences

Faculty of Medicine and Health Sciences Research Ethics Ref: FMHS 462-0222

For peer review only

This study investigates the opinions, experiences and approaches of healthcare professionals ***(excluding ENT/audiology specialists)*** who deal with adults with traumatic brain injury, with a focus on the auditory and balance status of these patients.

For peer review only

Thank you for your interest. You are invited to take part because you are a healthcare professional ***(excluding ENT/audiology specialists)*** who works with adult traumatic brain injury patients. Please read through the following information before agreeing to participate. You can ask any questions before deciding by contacting the researchers (details below). Taking part is entirely voluntary.

What will I be asked to do?

After clicking the 'next' button at the end of this information page you will be asked to provide informed consent to participate in this study. You will then be asked to provide some basic demographic information related to your profession (i.e. profession, years of occupation, type of institution) and will also answer some questions about your experiences, opinions, and approaches in relation to audio-vestibular findings of traumatic brain injury patients. It should take you a maximum of 20 minutes to complete. We would like you to answer all questions as honestly and completely as possible. You can withdraw at any point during the questionnaire for any reason, before submitting your answers by clicking the Exit button/closing the browser. The data will only be uploaded on completion of the questionnaire by clicking the FINISH button on the final page. At this point, it will not be possible to withdraw your answers.

What are the disadvantages of taking part?

We are asking you to give up your valuable time to take part and this is appreciated.

What are the advantages of taking part?

Your contribution, along with others, will contribute to researchers' understanding of the awareness of healthcare professionals about the auditory and balance status of patients with traumatic brain injury. It will also contribute to the understanding of the international determination of the approaches to audio-vestibular problems in these patients. Future research may also be conducted based on the results of this study.

Who will know I have taken part in the study?

No one will know you have taken part in this study because we will not ask for your name or any other personal identification details (ID) during this survey. Your IP address will not be visible to or stored by the research team because an online survey platform is being used which receives and stores an IP address but enables this detail to be filtered out before it is transferred to the research team. As with any online related activity, the risk of breach is possible but this risk is being minimised by using a platform on an encrypted webpage.

For peer review only

What will happen to your data?

When you have clicked the submit button at the end of the questionnaire, it will be uploaded into a password-protected database with a code number. The research team will not be able to see who it is from and for this reason, it will not be possible to withdraw the data at this point. Your data (research data) will be stored in a password-protected folder on a restricted-access server at the university under the terms of its data protection policy. Data is kept for a minimum of 7 years and then destroyed.

This survey is for a PhD project and the answers received from all participants will be combined in a password-protected database ready for analysis. The results will be written up as a dissertation and may be used in academic publications and presentations. The overall anonymised data from this study may be shared for use in future research and teaching (with research ethics approval).

The only personal data we will receive is your e-mail if you contact us to ask further questions or need support. This will be received and handled separately from your completed questionnaire and it will not be possible to link the sets of data. Your e-mail address will only be kept as long as needed to resolve your problem. It will then be destroyed. For further information about how the university processes personal data please see: https://www.nottingham.ac.uk/utilities/privacy.aspx/

Who will have access to your data?

The University of Nottingham is the data controller (legally responsible for data security) and the Supervisor of this study (named above) is the data custodian (manages access to the data) and as such will determine how your data is used in the study. Your research and personal data will be used for the purposes of the research only. Research is a task that we perform in the public interest.

Responsible members of the University of Nottingham may be given access to data for monitoring and/or audit of the study to ensure it is being carried out correctly.

If you have any questions or concerns about this project, please contact:

Kubra Bolukbas, E-mail: kubra.bolukbas@nottingham.ac.uk or

if you have any concerns about any aspect of this study please contact the Research Supervisor:

Kathryn Fackrell, Email: kathryn.fackrell@nottingham.ac.uk;

Laura Edwards, Email: laura.edwards@nottingham.ac.uk

For peer review only

If you remain unhappy and wish to complain formally, you should then contact the FMHS Research Ethics Committee Administrator E-mail:

FMHS-ResearchEthics@nottingham.ac.uk

For peer review only

Page 2: Consent Form

Please confirm each box to continue. If you do not accept the consent form, please close the browser. ✱ *Required*

Please select at least 5 answer(s).

- [ ] I confirm that I have read and understood the information on the previous page
- [ ] I am a healthcare professionals dealing with adult traumatic brain injury patients (other than audiologist and/or ENT specialist)
- [ ] I understand that my participation is voluntary and I can end the study at any time and withdraw my data by clicking the EXIT button
- [ ] I understand that my answers are anonymous
- [ ] I understand the overall anonymized data from this study may be used in the future for research (with research ethics approval) and teaching purposes

For peer review only

Page 3: Experiences and opinions of healthcare professionals about the audio-vestibular findings of individuals with traumatic brain injury

1. What is your profession? * Required

- Neurologist
- Neuropsychiatrist
- Neurosurgeon
- General Surgeon
- Physiatrist or Rehabilitation Medicine Doctor
- Geriatrician
- Orthopaedic Surgeon
- Rehabilitation Nurse
- Neuropsychologist
- Speech and Language Therapist
- Occupational Therapist
- Physiotherapist
- Psychologist
- Other

1.a. If you selected Other, please specify:

2. How many years have you been in your occupation? * Required

- Less than 5 years
- 5-10 years
- 10-20 years

For peer review only - http://bmjopen.bmj.com/site/about/guidelines.xhtml

- More than 20 years

3. How many years have you been working with adult patients with traumatic brain injury? ✱ *Required*

- Less than 5 years
- 5-10 years
- 10-20 years
- More than 20 years

4. What country do you work in? ✱ *Required*

- Australia
- Azerbaijan
- Belgium
- Canada
- China
- Cyprus
- Denmark
- France
- Germany
- Greece
- India
- Ireland
- Israel
- Italy
- Japan
- Netherlands
- Poland

- Saudi Arabia
- Spain
- Sweden
- Switzerland
- Thailand
- Türkiye
- United Kingdom
- United States of America
- Other

4.a. If you selected Other, please specify:

5. Is your place of work private or state-owned? * Required

- Private
- State-owned

6. How many hours per week do you spend working with traumatic brain injury adult patients? * Required

- Less than an hour
- 1-5 hours
- 5-10 hours
- 10-20 hours
- More than 20 hours

For peer review only

7. The following symptoms may be reported by people who have had a traumatic brain injury. Please indicate how frequently your adult patients, who experienced trauma more than 6 months ago, describe these to you * *Required*

Please don't select more than 1 answer(s) per row.

Please select at least 12 answer(s).

	All of my patients describe this	Most of my patients describe this	Some of my patients describe this	A few of my patients describe this	None of my patients describe this	Do not know
Hearing loss	☐	☐	☐	☐	☐	☐
Inability to understand speech in quiet	☐	☐	☐	☐	☐	☐
Inability to understand speech in noise	☐	☐	☐	☐	☐	☐
Inability to identify where the sound is coming from	☐	☐	☐	☐	☐	☐
Inability to capture conversations	☐	☐	☐	☐	☐	☐
Tinnitus (ringing or buzzing in the ears)	☐	☐	☐	☐	☐	☐
Hyperacusis (an unusual tolerance to ordinary environmental sounds)	☐	☐	☐	☐	☐	☐
Dizziness	☐	☐	☐	☐	☐	☐
Vertigo	☐	☐	☐	☐	☐	☐

Inability to walk in the dark without a physical problem	◻	◻	◻	◻	◻	◻
Dizziness on different surfaces	◻	◻	◻	◻	◻	◻
Feeling dizzy when asleep or awake due to movement	◻	◻	◻	◻	◻	◻

8. The following symptoms may be reported and/or observed by people who have had a traumatic brain injury. Please indicate how frequently, adults who experienced trauma more than 6 months ago, describe these to you, *Required

Please don't select more than 1 answer(s) per row.

Please select at least 10 answer(s).

	All of my patients	More than half of my patients	Some of my patients	A few of my patients	None of my patients	Do not know
Asks you to repeat your speech frequently during your interviews	◻	◻	◻	◻	◻	◻
Reading your lips while you speak	◻	◻	◻	◻	◻	◻
Does not understand you when you are not facing her/him	◻	◻	◻	◻	◻	◻
Difficulty understanding you on the phone	◻	◻	◻	◻	◻	◻

For peer review only - http://bmjopen.bmj.com/site/about/guidelines.xhtml

Relatives report complaints that the volume is too high while he/she is watching TV	☐	☐	☐	☐	☐	☐
Avoids coming to appointments because they are concerned they will not understand what you are talking about	☐	☐	☐	☐	☐	☐
Is disturbed by environmental/external sounds during their appointment with you	☐	☐	☐	☐	☐	☐
Reports an inability to tolerate certain sounds	☐	☐	☐	☐	☐	☐
Although their physical and mental problems are partially healed, audio-vestibular impairments affect their activities, such as doing sports, dancing and housework	☐	☐	☐	☐	☐	☐
Being afraid to leave the house without an accompanying person because of their balance disorders	☐	☐	☐	☐	☐	☐

9. Do you ask your adult patients with traumatic brain injury (which occurred more than 6 months ago) about their balance status (dizziness, vertigo etc.) at your appointments? * *Required*

- Yes, I ask everybody
- If their balance status prevents me from performing our appointments and/or my job, I ask
- I prefer not to ask because it is not my expertise
- I do not ask anything if there are no complaints about it
- Do not know

10. If you notice dizziness or balance disorders in your adult patients with traumatic brain injury (which occurred more than 6 months ago), which of the screening methods would you use? You can choose multiple options *✱ Required*

- Past Pointing test
- Finger-to-Nose test
- Unterberger test (Fukuda step test)
- Romberg test
- Gait analysis
- Eye movements
- Head impulse test
- I prefer not to assess this because it is not my expertise
- Do not know
- Other

10.a. If you selected Other, please specify:

11. In which situation would you consider referring your traumatic brain injury patient (where the trauma occurred more than 6 months ago) to the ENT and/or audiology service? You can choose multiple options *✱ Required*

For peer review only - http://bmjopen.bmj.com/site/about/guidelines.xhtml

- When I think he/she don't understand my speech and/or don't hear, immediately
- When I observe dizziness and/or vertigo, immediately
- If she/he reports tinnitus or hyperacusis, immediately
- I would wait for our next appointment to be sure, even if I notice his/her condition
- Unless the patient complains, I do not refer
- I think it is an issue that can be ignored as there are many impairments they are struggling with
- Do not know

12. How many of your adult patients who have had a traumatic brain injury (which occurred more than 6 months ago) do you refer to the audiology or ENT department? * *Required*

- All of my patients
- More than half of my patients
- Some of my patients
- A few of my patients
- None of my patients
- Do not know

13. How do you check the external ear condition of your adult patients with traumatic brain injury (which occurred more than 6 months ago)? * *Required*

- Referring to the ENT and/or audiology service
- I prefer to evaluate it myself with otoscopy
- I do not do anything if there are no complaints about it
- I am not interested because it is not my area of expertise
- Do not know

For peer review only

14. Do you think that your traumatic brain injury patients (where the trauma occurred more than 6 months ago) should use hearing aids if they have hearing loss?
* Required

- Yes, definitely
- If they would like, yes
- No need
- I think it is an issue that can be ignored as there are many impairments they are struggling with
- Do not know

15. In which situations can you help your adult patients with traumatic brain injury, who use hearing aids, during their appointment with you? You can choose multiple options
* Required

- [ ] I can change the battery of hearing aids
- [ ] I can notice whether or not it is working
- [ ] I can notice any feedback problem
- [ ] I can check the hearing aid ear mould and/or dome functionality
- [ ] I do not know anything about hearing aids

16. Did your patient group, who frequently report auditory and/or vestibular complaints after trauma, have a specific aetiology of traumatic brain injury? Can you generalize the trauma aetiology of these patients with auditory and/or vestibular complaints?
* Required

- Yes
- No
- Do not know

For peer review only

16.a. If you answered yes to question 16, what was this aetiology?

- Falls
- Vehicle-related collisions
- Violence
- Sport Injuries
- Other Trauma (Please describe)

16.a.i. If you selected Other, please specify:

For peer review only

Turkish Version of Survey

Sağlık Çalışanları ve Travmatik Beyin Hasarının İşitsel-vestibüler Sonuçları

Page 1: Bilgilendirme Formu

Bilgilendirme ve Onam Formu

Final version 1.2: 04.07.2022

Araştırma Başlığı: Travmatik beyin hasarı olan bireylerin odyo-vestibüler bulgularına ilişkinsağlık profesyonellerinin deneyimleri ve görüşleri

Araştırma Ekibi:

Danışmanlar:

Dr. Kathryn Fackrell, İşitme Bilimleri

Dr. Laura Edwards, Tıp ve Sağlık Bilimleri

Araştırmacı:

Uzm Ody. Kübra Bölükbaş, İşitme Bilimleri Doktora Öğrencisi

Tıp ve Sağlık Bilimleri Fakültesü Araştırma Etiği Ref. No: FMHS 462-0222

Bu çalışma, ***KBB ve odyoloji uzmanlarının dışındaki***, travmatik beyin hasarı olan erişkinlerleilgilenen sağlık profesyonellerinin görüş, deneyim ve yaklaşımlarını bu hastaların işitsel ve denge durumlarına odaklanarak incelemektedir.

İlgiliniz için teşekkür ederiz. ***KBB/odyoloji uzmanları dışında*** travmatik beyin hasarı olan yetişkin hastalarla ilgilenen bir sağlık çalışanı olduğunuz için çalışmamıza katılmaya davetlisiniz. Lütfen katılımcı olmadan önce bilgilendirme ve onam formunu dikkatlice okuyunuz.Herhangi bir sorunuz varsa aşağıda yer alan iletişim kanalları

For peer review only

aracılığıyla araştırmacılara ulaşmaktan lütfen çekinmeyin. Araştırmaya katılım gönüllülük esasına dayanmaktadır.

For peer review only

Ne yapmanızı isteyeceğiz?

Bilgilendirme sayfasının sonundaki "Next (İleri)" butonuna tıkladıktan sonra, bu çalışmaya katılabilmeniz için onam formunu kabul etmeniz istenecektir. Daha sonra, mesleğinizle ilgili demografik bilgiler (mesleğiniz, çalışma süreniz, kurum türü gibi) içeren birkaç soru ve travmatikbeyin hasarı olan yetişkin hastalarınızın işitsel-vestibüler bulgularıyla ilgili deneyimleriniz, görüşleriniz ve yaklaşımlarınızı öğrenmek amacıyla hazırlanan soruları cevaplamanız istenecektir. Tüm anket sorularını cevaplamanız en fazla yirmi (20) dakikanızı alacaktır. Tüm soruları mümkün olduğunca dürüst ve eksiksiz olarak cevaplamanızı rica ediyoruz.

Cevaplarınızı göndermeden önce, "Çıkış" butonuna tıklayarak ve/veya tarayıcınızı kaparatarak herhangi bir noktada, herhangi bir nedenle anketten çekilebilirsiniz. Ancak, son

sayfada bulunan "Bitir (Finish)" butonuna tıkladıktan sonra cevaplarınız tarafımıza gönderilecekve cevaplarınızı geri çekmeniz mümkün olmayacaktır.

Katılımcı olmanızın dezavantajları nelerdir?

Katılımcı olmanız için kıymetli zamanınızdan birkaç dakikanızı anketimize ayırmanız gerekecektir.

Katılımcı olmanızın avantajları nelerdir?

Diğer katılımcılar ve sizin katkınız sayesinde araştırmacılar, travmatik beyin hasarı olan bireylerin işitsel ve vestibüler durumları hakkında KBB ve odyoloji uzmanları haricindeki sağlık uzmanlarının görüşleri, yaklaşımları ve farkındalıkları hakkında bilgi edineceklerdir. Ayrıca, bu bireylerdeki işitsel-vestibüler sorunlara yaklaşımın uluslararası düzeyde nasıl olduğunun tespit edilmesine katkı sağlanacaktır. Böylece, çalışmanın sonuçlarından yola çıkılarak konuya ilişkingelecek araştırmaların da önü açılabilir.

Araştırmacılar çalışmaya katıldığınızı bilecekler mi?

İsim, soyisim ve kimlik bilgisi gibi kişisel verileriniz sorulmadığı için hiç kimse araştırmaya katıldığınızı bilmeyecektir. Ayrıca, cevaplarınız araştırma ekibine iletilirken kullanılan platformunfiltreleme sistemi sayesinde IP adresiniz gözükmeyecek ve saklanmayacaktır. Tüm çevrimiçi platformlarda ihlal riski mümkün olduğu gibi bu platformda da mümkündür. Ancak kişisel veri alınmaması ve şifreli bir sistemin kullanılması sayesinde bu risk minimuma düşürülmüştür.

Verilerinize ne olacak?

Anketin sonundaki "Bitir (Finish)" butonuna tıkladığınızda, anket sonuçlarınız şifre korumalı birveri tabanına kod numarası ile yüklenecektir. Araştırma ekibi anketin kim tarafından cevaplandığını göremeyecek ve bu nedenle, bu noktada, verileri geri çekmeniz

For peer review only

mümkün olmayacaktır. Araştırma verileriniz, Nottingham Üniversitesinin veri koruma politikasışartlarına göre sınırlı erişimli bir sunucuda şifre korumalı bir klasörde saklanacaktır. Veriler enaz 7 yıl saklandıktan sonra imha edilecektir.

Bu anket bir doktora projesi için hazırlanmıştır. Tüm katılımcılardan elde edilen yanıtlar analizedilmeye hazır şifre korumalı bir veritabanında birleştirilecektir. Sonuçlar doktora kapsamındabir tez olarak yazılmasının yanısıra akademik yayınlarda ve sunumlarda kullanılabilir. Bu çalışmadan elde edilen anonimleştirilmiş veriler, etik onayı ile gelecekteki araştırma ve öğrenimde kullanılmak üzere paylaşılabilir.

Daha fazla soru sormak için veya desteğe ihtiyaç duyduğunuzda bizimle iletişime geçerseniz, alacağımız tek kişisel veri e-postanız olacaktır. Ancak bu, tamamlamış olduğunuz anketinizdenayrı olarak ele alınacaktır ve verilerinizi birbirine bağlamak mümkün olmayacaktır. E-posta adresiniz yalnızca sorununuzu çözmek için gerektiği kadar saklanacak ve daha sonra imha edilecektir. Üniversitenin kişisel verileri nasıl işlediği hakkında daha fazla bilgi için lütfen şu adresi ziyaret ediniz: https://www.nottingham.ac.uk/utilities/privacy.aspx/

Verilerinize kim erişebilecek?

Nottingham Üniversitesi veri denetleyicisidir (veri güvenliğinden yasal olarak sorumludur) ve buçalışmanın danışmanları (yukarıda adı geçen) veri sorumlusudur (verilere erişimi yönetmektedirler) ve bu nedenle verilerinizin çalışmada nasıl kullanılacağını belirleyeceklerdir. Yanıtlarınız ve verileriniz yalnızca araştırma amacıyla kullanılacaktır. Araştırmamız, kamu yararını gözeterek gerçekleştirdiğimiz bir projedir.

Nottingham Üniversitesi'nin sorumlu üyelerine, çalışmanın doğru bir şekilde yürütüldüğündenemin olmak için izleme ve/veya denetleme için verilere erişim izni verilebilir.

Bu projeyle ilgili herhangi bir sorunuz veya endişeniz varsa, lütfen iletişime geçiniz:

Kübra Bölükbaş, E-mail: kubra.bolukbas@nottingham.ac.uk or

Bu çalışmanın herhangi bir yönü hakkında bir endişeniz varsa, lütfen araştırma danışmanları ileiletişime geçiniz:

Kathryn Fackrell,

Email: kathryn.fackrell@nottingham.ac.uk

Laura Edwards,

Email: laura.edwards@nottingham.ac.uk

For peer review only

Resmi olarak şikayette bulunmak isterseniz, FMHS Araştırma Etik Kurulu Yöneticisi ile iletişimegeçiniz:

FMHS-ResearchEthics@nottingham.ac.uk

For peer review only

Page 2: Onam Formu

Please select at least 5 answer(s).

- ☐ Önceki sayfadaki bilgileri okuduğumu ve anladığımı onaylıyorum
- ☐ Odyolog ve/veya KBB uzmanı dışında, travmatik beyin hasarı olan yetişkinlerle ilgilenen bir sağlık çalışanıyım
- ☐ Katılımımın gönüllü olduğunu ve çalışmayı istediğim zaman sonlandırabileceğimi ve sayfayı kapatarak verilerimi geri çekebileceğimi anlıyorum
- ☐ Yanıtlarımın anonim olarak görüneceğini anlıyorum
- ☐ Bu çalışmadan elde edilen anonimleştirilmiş verilerin gelecekte etik onayı ile araştırma ve öğrenim amaçları için kullanılabileceğini anlıyor ve kabul ediyorum

Devam edebilmek için lütfen her bir kutucuğu onaylayın. Bilgilendirme ve onam formunukabul etmiyorsanız, lütfen tarayıcınızı kapatarak çıkış yapınız. ✱*Required*

For peer review only

Page 3: Travmatik Beyin Hasarı olan Bireylerin Odyo-vestibülerBulgularına İlişkin Sağlık Profesyonellerinin Deneyimleri ve Görüşleri

1. Mesleğiniz nedir? ✱ *Required*

- ○ Nörolog
- ○ Nöropsikiyatrist
- ○ Beyin Cerrahı
- ○ Genel Cerrahi Uzmanı
- ○ Fiziksel Tıp ve Rehabilitasyon Uzmanı
- ○ Geriatri Uzmanı
- ○ Ortopedi ve Travmatoloji Uzmanı
- ○ Hemşire
- ○ Nöropsikolog
- ○ Dil ve Konuşma Terapisti
- ○ Ergoterapist
- ○ Fizyoterapist
- ○ Psikolog
- ○ Diğer Sağlık Profesyoneli (Lütfen açıklayınız)

1.a. Diğer seçeneğini seçtiyseniz, lütfen açıklayın:

2. Kaç yıldır mesleğinizi yapıyorsunuz?✱ *Required*

- ○ 5 yıldan az
- ○ 5 ile 10 yıl arası
- ○ 10 ile 20 yıl arası
- ○ 20 yılı aşkın süredir

3. Travmatik beyin hasarı olan hastalarla kaç yıldır çalışıyorsunuz? * Required

- 5 yıldan az
- 5 ile 10 yıl arası
- 10 ile 20 yıl arası
- 20 yılı aşkın süredir

4. Hangi ülkede çalışıyorsunuz? * Required

- Almanya
- Amerika
- Avustralya
- Azerbaycan
- Belçika
- Birleşik Krallık
- Çin
- Danimarka
- Hindistan
- Hollanda
- İrlanda
- İspanya
- İsrail
- İsveç
- İsviçre
- İtalya
- Japonya
- Kanada
- Kıbrıs
- Polonya
- Suudi Arabistan
- Türkiye

- Yunanistan
- Diğer

4.a. Diğer seçeneğini seçtiyseniz, lütfen açıklayın:

5. İş yeriniz özel mi yoksa devlete mi ait?✱ *Required*

- Özel
- Devlet

6. Travmatik beyin hasarı olan yetişkin hastalarla haftada kaç saat çalışıyorsunuz? ✱ *Required*

- 1 saatten az
- 1 ile 5 saat arası
- 5 ile 10 saat arası
- 10 ile 20 saat arası
- 20 saatten fazla

7. Aşağıdaki şikayetler travmatik beyin hasarı geçirmiş kişiler tarafından bildirilebilir. Lütfen 6 aydan daha uzun süre önce kafa travmasına maruz kalmış hastalarınızın bunları size ne sıklıklatarif ettiğini belirtiniz. ✱ *Required*

Please don't select more than 1 answer(s) per row.

Please select at least 12 answer(s).

	Bütün hastaları mbunu bildiriyor	Hastalarımı nçoğu bunu bildiriyor	Bazı hastaları mbunu bildiriyor	Birkaç hastam bunu bildiriyor	Hiçbir hastam bunu bildirmiyor	Bilmiyorum
İşitme kaybı	☐	☐	☐	☐	☐	☐
Sessiz ortamda konuşmayı anlayamama	☐	☐	☐	☐	☐	☐
Gürültülü ortamda konuşmayı anlayamama	☐	☐	☐	☐	☐	☐
Sesin nereden geldiğini tespit edememe	☐	☐	☐	☐	☐	☐
Konuşmaları yakalayamama	☐	☐	☐	☐	☐	☐
Kulak çınlaması (Tinnitus)	☐	☐	☐	☐	☐	☐
Normal çevresel seslere karşı olağan dışı hassasiyet, tahammülsüzlük (Hiperakuzi)	☐	☐	☐	☐	☐	☐
Dengesizlik, Sersemlik (Dizziness)	☐	☐	☐	☐	☐	☐
Baş Dönmesi (Vertigo)	☐	☐	☐	☐	☐	☐
Fiziksel bir problemi olmaksızın karanlıkta yürüyememe	☐	☐	☐	☐	☐	☐

Farklı yüzeylerde yürürken dengesizlik (dizziness)	☐	☐	☐	☐	☐	☐
Harekete bağlı olarak uykuda veya uyanıkken baş dönmesi hissetmek	☐	☐	☐	☐	☐	☐

8. Aşağıdaki belirtiler travmatik beyin hasarı geçirmiş kişiler tarafından bildirilebilir ve/veyagözlemlenebilir. Lütfen 6 aydan daha uzun süre önce kafa travmasına maruz kalmış hastalarınızın bunları size ne sıklıkla bildirdiğini belirtiniz ✱*Required*

Please don't select more than 1 answer(s) per row.

Please select at least 10 answer(s).

	Hastalarımın hepsi	Hastalarımın yarısından fazlası	Bazı hastalarım	Birkaç hastam	Hastalarımın hiçbiri bunu bildirmiyor	Bilmiyorum
Görüşmeleriniz sırasında konuşmanızı sıksık tekrarlamanızı ister	☐	☐	☐	☐	☐	☐
Konuşurken dudaklarınızı okur	☐	☐	☐	☐	☐	☐
Yüzünüz ona dönük olmadığında sizianlamaz	☐	☐	☐	☐	☐	☐
Telefonda sizi anlamakta zorluk çeker	☐	☐	☐	☐	☐	☐

Hasta yakınları hastanın çok yüksek sesle televizyon izlemeye başladığından şikayet eder	☐	☐	☐	☐	☐	☐
Ne hakkında konuştuğunuzu anlayamamaktan endişelendiği için randevularınıza gelmekten kaçınır	☐	☐	☐	☐	☐	☐
Sizinle görüşmesi sırasında çevresel/dış seslerden rahatsız olur	☐	☐	☐	☐	☐	☐
Belirli seslere tahammül edemediğini bildirir	☐	☐	☐	☐	☐	☐
Fiziksel ve zihinsel sorunları kısmen iyileşmiş olsa da, işitsel-vestibüler bozukluklar spor yapma, dans etme ve ev işi yapma gibi aktivitelerini etkiler	☐	☐	☐	☐	☐	☐

Denge bozuklukları nedeniyle refakatçi olmadan evden çıkmaya korkar	☐	☐	☐	☐	☐	☐

9. 6 aydan daha uzun süre önce meydana gelen travmatik beyin hasarına maruz kalmış hastalarınıza randevularınızda denge durumlarının nasıl olduğunu (sersemlik, başdönmesi vb.) soruyor musunuz? ✱*Required*

- ○ Evet, tüm hastalarıma sorarım
- ○ Denge durumları randevularımızı etkiliyorsa ve/veya işimi yapmamı engelliyorsa sorarım
- ○ Bu konu uzmanlık alanım olmadığı için sormamayı tercih ediyorum
- ○ Bu konuda herhangi bir şikayeti yoksa hiçbir şey sormam
- ○ Bilmiyorum

10. 6 aydan daha uzun süre önce travmatik beyin hasarına maruz kalan hastalarınızda baş dönmesi ve/veya denge bozukluğu fark ederseniz, aşağıdaki tarama yöntemlerinden hangisiniveya hangilerini kullanırsınız? Birden fazla yanıt seçebilirsiniz. ✱ *Required*

- ☐ Past Pointing testi
- ☐ Parmak Burun testi
- ☐ Unterberger testi (Fukuda adım
- ☐ testi)Romberg testi
- ☐ Gait analysis (Yürüme analizi)
- ☐ Göz hareketleri
- ☐ Head impulse testi
- ☐ Uzmanlık alanım olmadığı için değerlendirmeyi tercih etmiyorum
- ☐ Bilmiyorum
- ☐ Diğer

10.a. Diğer seçeneğini seçtiyseniz, lütfen açıklayın:

11. 6 aydan daha uzun süre önce travmatik beyin hasarına maruz kalmış hastanızı hangidurum veya durumlarda KBB ve/veya odyoloji departmanına sevk etmeyi düşünürsünüz? Birden fazla yanıt seçebilirsiniz.* *Required*

- [ ] Konuşmamı anlamadığını ve/veya duymadığını düşündüğümde hemen
- [ ] Dizziness ve/veya baş dönmesi gözlemlediğimde hemen
- [ ] Kulak çınlaması veya seslere karşı hassasiyet bildirirse hemen
- [ ] Durumunu fark etsem bile emin olmak için bir sonraki randevuyu beklerim
- [ ] Hasta herhangi bir şikayet bildirmedikçe sevk etmiyorum
- [ ] Bu hasta grubunun uğraştığı çok fazla problemi olduğu için görmezden gelinebilecek bir konu olduğunu düşünüyorum
- [ ] Bilmiyorum

review

12. 6 aydan daha uzun süre önce travmatik beyin hasarına maruz kalmış hastalarınızdantahmini olarak kaç tanesini odyoloji ve/veya KBB departmanına yönlendiriyorsunuz? **Required*

- ○ Hastalarımın hepsini
- ○ Hastalarımın yarısından
- ○ fazlasınıBazı hastalarımı
- ○ Birkaç hastamı
- ○ Hastalarımın hiçbirini yönlendirmiyorum
- ○ Bilmiyorum

13. 6 aydan daha uzun süre önce travmatik beyin hasarına maruz kalmış hastalarınızın gerektiğinde, dış kulaklarının durumunu nasıl kontrol edersiniz? * *Required*

- ○ KBB hekimine ve/veya odyoloji departmanına konsülte ederim

- Otoskop ile kendim değerlendirmeyi tercih ederim
- Bu konuda şikayeti yoksa hiçbir şey yapmam
- Uzmanlık alanım olmadığı için ilgilenmiyorum
- Bilmiyorum

14. 6 aydan daha önce travmatik beyin hasarına maruz kalan hastanızın işitme kaybı varsaişitme cihazı kullanması gerektiğini düşünüyor musunuz? ✱ *Required*

- Evet, kesinlikle kullanmalı
- İsterse, evet
- Gerek yok
- Çok fazla problemle uğraştıkları için görmezden gelinebilecek bir konu olduğunu düşünüyorum
- Bilmiyorum

15. İşitme cihazı kullanan travmatik beyin hasarına maruz kalmış hastalarınıza randevularısırasında işitme cihazlarıyla ilgili hangi durumlarda yardımcı olabilirsiniz? Birden fazla yanıt seçebilirsiniz. ✱ *Required*

- İşitme cihazlarının pilini değiştirebilirim
- Hastamın işitme cihazının çalışıp çalışmadığını anlayabilirim
- Herhangi bir feedback (ötme sesi) sorunu varsa fark edebilirim
- İşitme cihazının kulak kalıbını ve/veya dome (kubbe) işlevini kontrol edebilirim
- İşitme cihazları hakkında hiçbir şey bilmiyorum

16. Travma sonrası sıklıkla işitsel ve/veya vestibüler şikayetler bildiren hasta grubunuz,travmatik beyin hasarının spesifik bir etiyolojisine sahip miydi? İşitsel ve/veya vestibüler şikayetleri olan bu hastaların travma etiyolojisini genelleyebilir misiniz ? ✱ *Required*

- Evet

- Hayır
- Bilmiyorum

16.a. 17. soruya evet cevabı verdiyseniz, bu etiyoloji neydi?

- Düşme
- Trafik kazası
- Şiddet
- Spor yaralanmaları
- Diğer nedenler(Lütfen açıklaynız)

16.a.i. Diğer seçeneğini seçtiyseniz, lütfen açıklayın:

For peer review only

Supplemental Appendix 2. List of organisations that were contacted to share the surveys

Name of Organisations	Country
Speech Pathology Australia	Australia
Rehabilitation Medicine Society of Australia and New Zealand	Australia and New Zealand
the Royal Australian and New Zealand College of Psychiatrist	Australia and New Zealand
Speech-Language & Audiology Canada	Canada
Hellenic Neuropsychological Society	Greece
Logopedista	Italy
Association of Occupational Therapists of Ireland	Ireland
Irish Association of Speech & Language Therapists	Ireland
Headway The Brain Injury Association	United Kingdom
The British Neuropsychological Society	United Kingdom
British Psychological Society	United Kingdom
The British Neuropsychiatry Association	United Kingdom
Northampton Speech Therapy (Independent Therapist)	United Kingdom
British Society of Rehabilitation Medicine	United Kingdom
Royal College of Speech and Language Therapists	United Kingdom
Association of Speech and Language Therapists	Türkiye
World Federation of Occupational Therapists	

For peer review only

Supplemental Appendix 3. The results of statistical analysis on associations between audio-vestibular symptoms observed by HCPs or reported by patients' relatives to HCPs, and the years working with TBI adults, groups of country of residence and groups of occupations.

A statistically significant difference was observed in the years of working with TBI adults, in the case of 'being afraid to leave the house without an accompanying person because of their balance disorders' in the Kruskal-Wallis test ($p<0.05$). The statistical differences were identified between the groups working with adults with TBI for 5-10 years and 10-20 years (Appendix 3-Table 1), as well as between the groups working with adults with TBI for 5-10 years and more than 20 years in the Mann-Whitney U test with Bonferroni correction ($p<0.008$; Appendix 3-Table 2).

Ranks

	How many years have you been working with adult patients with traumatic brain injury?	N	Mean Rank	Sum of Ranks
Being afraid to leave the house without an accompanying person because of their balance disorders	5-10 years	20	14.88	297.50
	10-20 years	19	25.39	482.50
	Total	39		

Test Statistics[a]

	Being afraid to leave the house without an accompanying person because of their balance disorders
Mann-Whitney U	87.500
Wilcoxon W	297.500
Z	-2.970
Asymp. Sig. (2-tailed)	.003
Exact Sig. [2*(1-tailed Sig.)]	.003[b]

a. Grouping Variable: How many years have you been working with adult patients with traumatic brain injury?

Appendix 3-Table 1: The comparison of 5-10 years and 10-20 years groups in the case of 'being afraid to leave the house without an accompanying person because of their balance disorders' via the Mann-Whitney U test with Bonferroni correction

Ranks

	How many years have you been working with adult patients with traumatic brain injury?	N	Mean Rank	Sum of Ranks
Being afraid to leave the house without an accompanying person because of their balance disorders	5-10 years	20	13.75	275.00
	More than 20 years	15	23.67	355.00
	Total	35		

Test Statistics[a]

	Being afraid to leave the house without an accompanying person because of their balance disorders
Mann-Whitney U	65.000
Wilcoxon W	275.000
Z	-2.934
Asymp. Sig. (2-tailed)	.003
Exact Sig. [2*(1-tailed Sig.)]	.004[b]

a. Grouping Variable: How many years have you been working with adult patients with traumatic brain injury?

Appendix 3-Table 2: The comparison of 5-10 years and more than 20 years groups in the case of 'being afraid to leave the house without an accompanying person because of their balance disorders' via the Mann-Whitney U test with Bonferroni correction

For peer review only

Majority of HCPs (n=13, 19%) who work with TBI adults for 5-10 years stated that ‘more than half' and ‘some’ of their patients reported ‘being afraid to leave the house without an accompanying person because of their balance disorders’, whilst most of the participants who work with TBI patients for 10-20 years (n=12, 17%) and more than 20 years (n=11, 16%) indicated that this case was observed in ‘some’ and ‘a few’ of their patients.

Furthermore, there were statistically significant differences for symptoms of ‘asking to repeat their speech frequently during their interviews’, ‘relatives reporting complaints that the volume is too high while he/she is watching TV’ and ‘avoiding coming to appointments because TBI patients are concerned they will not understand what HCPs are talking about’ based on groups of country of residence in the Kruskal-Wallis test ($p<0.05$). Statistically significant differences were noted between the UK and Asia for all three symptoms as seen in Appendix 3-Table 3.

Ranks

	What country do you work in?	N	Mean Rank	Sum of Ranks
Asks you to repeat your speech frequently during your interviews	Asia	15	15.00	225.00
	United Kingdom	30	27.00	810.00
	Total	45		
Relatives report complaints that the volume is too high while he/she is watching TV	Asia	15	12.87	193.00
	United Kingdom	30	28.07	842.00
	Total	45		
Avoids coming to appointments because they are concerned they will not understand what you are talking about	Asia	15	15.80	237.00
	United Kingdom	30	26.60	798.00
	Total	45		

Test Statistics[a]

	Asks you to repeat your speech frequently during your interviews	Relatives report complaints that the volume is too high while he/she is watching TV	Avoids coming to appointments because they are concerned they will not understand what you are talking about
Mann-Whitney U	105.000	73.000	117.000
Wilcoxon W	225.000	193.000	237.000
Z	-3.109	-3.803	-2.694
Asymp. Sig. (2-tailed)	.002	<.001	.007

a. Grouping Variable: What country do you work in?

Appendix 3-Table 3: The comparison of the UK and Asia groups in the cases of ‘asking to repeat their speech frequently during their interviews’, ‘relatives reporting complaints that the volume is too high while he/she is watching TV’ and ‘avoiding coming to appointments because TBI patients are concerned they will not understand what HCPs are talking about’ via the Mann-Whitney U test with Bonferroni correction

Similarly, differences were observed between Asia and Others for the symptoms ‘asking to repeat their speech frequently during their interviews’ and ‘relatives reporting complaints that the volume is too high while he/she is watching TV’ ($p<0.008$; Appendix 3-Table 4).

Ranks

	What country do you work in?	N	Mean Rank	Sum of Ranks
Asks you to repeat your speech frequently during your interviews	Asia	15	10.57	158.50
	Others	15	20.43	306.50
	Total	30		
Relatives report complaints that the volume is too high while he/she is watching TV	Asia	15	10.53	158.00
	Others	15	20.47	307.00
	Total	30		
Avoids coming to appointments because they are concerned they will not understand what you are talking about	Asia	15	12.33	185.00
	Others	15	18.67	280.00
	Total	30		

Test Statistics[a]

	Asks you to repeat your speech frequently during your interviews	Relatives report complaints that the volume is too high while he/she is watching TV	Avoids coming to appointments because they are concerned they will not understand what you are talking about
Mann-Whitney U	38.500	38.000	65.000
Wilcoxon W	158.500	158.000	185.000
Z	-3.197	-3.187	-2.045
Asymp. Sig. (2-tailed)	.001	.001	.041
Exact Sig. [2*(1-tailed Sig.)]	.001[b]	.001[b]	.050[b]

a. Grouping Variable: What country do you work in?

b. Not corrected for ties.

Appendix 3-Table 4: The comparison of the UK and Others groups in the cases of ‘asking to repeat their speech frequently during their interviews’, ‘relatives reporting complaints that the volume is too high while he/she is watching TV’ and ‘avoiding coming to appointments because TBI patients are concerned they will not understand what HCPs are talking about’ via the Mann-Whitney U test with Bonferroni correction

For peer review only - http://bmjopen.bmj.com/site/about/guidelines.xhtml

Additionally, a statistical difference was observed regarding the symptom ‘relatives reporting complaints that the volume is too high while he/she is watching TV’ between Europe and the UK in the Mann-Whitney U test with Bonferroni correction (p<0.008; Appendix 3-Table 5).

Ranks

	What country do you work in?	N	Mean Rank	Sum of Ranks
Asks you to repeat your speech frequently during your interviews	Europe	10	14.60	146.00
	United Kingdom	30	22.47	674.00
	Total	40		
Relatives report complaints that the volume is too high while he/she is watching TV	Europe	10	12.05	120.50
	United Kingdom	30	23.32	699.50
	Total	40		
Avoids coming to appointments because they are concerned they will not understand what you are talking about	Europe	10	15.75	157.50
	United Kingdom	30	22.08	662.50
	Total	40		

Test Statistics[a]

	Asks you to repeat your speech frequently during your interviews	Relatives report complaints that the volume is too high while he/she is watching TV	Avoids coming to appointments because they are concerned they will not understand what you are talking about
Mann-Whitney U	91.000	65.500	102.500
Wilcoxon W	146.000	120.500	157.500
Z	-2.013	-2.776	-1.542
Asymp. Sig. (2-tailed)	.044	.006	.123
Exact Sig. [2*(1-tailed Sig.)]	.067[b]	.007[b]	.140[b]

a. Grouping Variable: What country do you work in?

b. Not corrected for ties.

Appendix 3-Table 5: The comparison of the UK and Others groups in the cases of ‘asking to repeat their speech frequently during their interviews’, ‘relatives reporting complaints that the volume is too high while he/she is watching TV’ and ‘avoiding coming to appointments because TBI patients are concerned they will not understand what HCPs are talking about’ via the Mann-Whitney U test with Bonferroni correction

Most of participants in the UK (n=27, 39%) and Others (n=10, 14%) groups reported that the symptom of ‘asking to repeat their speech frequently during their interviews’ was observed in ‘some’ to ‘a few’ of their patients, most participants in Asia (n=13, 19%) reported that this symptom was observed in ‘more than half’ to ‘some’ of their patients. Most participants from Asia (n=7, 10%) and Europe (n=6, 9%) reported that ‘some’ of their patients’ relatives complained about the volume being too high while they watch TV, whilst the majority of participants from the UK (n=15, 21%) mentioned that ‘a few’ of their patients’ relatives reported this issue. Conversely, the majority of participants from the Others group (n=10, 14%) stated that ‘a few’ to ‘none’ of their patients’ relatives reported this case. Most participants from Asia (n=6, 9%) reported that ‘a few’ of their patients avoided appointments because they were concerned that they would not understand what the HCPs were talking about. In contrast, most participants from the UK (n=11, 16%) reported that ‘none’ of their patients avoided appointments for this reason.

Another significant difference was observed between Groups 1 (physiotherapists) and 5 (doctors from other specialties) for the symptoms of ‘relatives reporting complaints that the volume is too high while he/she is watching TV’, ‘avoiding coming to appointments because TBI patients are concerned they will not understand what HCPs are talking about’, and ‘an inability to tolerate certain sounds’ in the Mann-Whitney U test with Bonferroni correction (p<0.003-Appendix 3-Table 6).

For peer review only

Test Statistics[a]

	Asks you to repeat your speech frequently during your interviews	Does not understand you when you are not facing her/him	Difficulty understanding you on the phone	Relatives report complaints that the volume is too high while he/she is watching TV	Avoids coming to appointments because they are concerned they will not understand what you are talking about	Reports an inability to tolerate certain sounds
Mann-Whitney U	76.500	73.000	45.000	44.500	34.000	46.000
Wilcoxon W	196.500	193.000	165.000	164.500	154.000	166.000
Z	-1.588	-1.713	-2.902	-2.949	-3.419	-2.996
Asymp. Sig. (2-tailed)	.112	.087	.004	.002	<.001	.002
Exact Sig. [2*(1-tailed Sig.)]	.137[b]	.106[b]	.004[b]	.004[b]	<.001[b]	.005[b]

a. Grouping Variable: What is your profession?

b. Not corrected for ties.

Appendix 3-Table 6: The comparison of the Groups 1and 5 in the cases of 'relatives reporting complaints that the volume is too high while he/she is watching TV', 'avoiding coming to appointments because TBI patients are concerned they will not understand what HCPs are talking about', and 'an inability to tolerate certain sounds' via the Mann-Whitney U test with Bonferroni correction

Most of the participants in Group 5 (n=8, 11%) stated that 'some' of their patients' relatives complained about the volume being too high while they watch TV, whilst the most participants of Group 1 (n=5, 7%) reported that 'none' of their patient relatives reported this problem. The majority of participants in Group 1 reported that 'none' of their TBI patients avoided coming to appointments because they were concerned, they would not understand what the HCPs were talking about. However, participants in Group 5 (n=4, 6% for each option) had equal percentages among the options of 'some', 'a few' and 'none' of their patients. The majority of participants in Group 1 (n=13, 19%) reported observing that 'some' to 'a few' of their patients reported an inability to tolerate certain sounds, whilst 17% of HCPs (n=12) in Group 5 reported observing this symptom in 'more than half' to 'some' of their patients.

For peer review only

Checklist for Reporting Results of Internet E-Surveys (CHERRIES)

Checklist Item	***Explanation***	***Page Number***
Describe survey design	Describe target population, sample frame. Is the sample a convenience sample? (In “open” surveys this is most likely.)	Page 5/ Lines 120-122,<br>Page 6/ Lines 159-164
IRB approval	Mention whether the study has been approved by an IRB.	Page 5/Lines 122-124<br>Page 17/Line 431-434
Informed consent	Describe the informed consent process. Where were the participants told the length of time of the survey, which data were stored and where and for how long, who the investigator was, and the purpose of the study?	Page 5/ Lines 124-128
Data protection	If any personal information was collected or stored, describe what mechanisms were used to protect unauthorized access.	Not collected any personal information
Development and testing	State how the survey was developed, including whether the usability and technical functionality of the electronic questionnaire had been tested before fielding the questionnaire.	Pages 5-6/ Lines 131-140, Lines 155-157
Open survey versus closed survey	An “open survey” is a survey open for each visitor of a site, while a closed survey is only open to a sample which the investigator knows (password-protected survey).	Page 6/ Line 150
Contact mode	Indicate whether or not the initial contact with the potential participants was made on the Internet. (Investigators may also send out questionnaires by mail and allow for Web-based data entry.)	Pages 6-7/ Lines 168-175
Advertising the survey	How/where was the survey announced or advertised? Some examples are offline media (newspapers), or online (mailing lists – If yes, which ones?) or banner ads (Where were these banner ads posted and what did they look like?). It is important to know the wording of the announcement as it will heavily influence who chooses to participate. Ideally the survey announcement should be published as an appendix.	Pages 6-7/ Lines 168-175 and Supplemental Appendix 2
Web/E-mail	State the type of e-survey (eg, one posted on a Web site, or one sent out through e-mail). If it is an e-mail survey, were the responses entered manually into a database, or was there an automatic method for capturing responses?	Page 6/ Lines 147-150
Context	Describe the Web site (for mailing list/newsgroup) in which the survey was posted. What is the Web site about, who is visiting it, what are visitors normally looking for? Discuss to what degree the content of the Web site could pre-select the sample or influence the results. For example, a survey about vaccination on a	NA

For peer review only

	anti-immunization Web site will have different results from a Web survey conducted on a government Web site	
Mandatory/voluntary	Was it a mandatory survey to be filled in by every visitor who wanted to enter the Web site, or was it a voluntary survey?	Page 7/ Line 177
Incentives	Were any incentives offered (eg, monetary, prizes, or non-monetary incentives such as an offer to provide the survey results)?	Page 7/ Line 177
Time/Date	In what timeframe were the data collected?	Page 7/ Lines 175-177
Randomization of items or questionnaires	To prevent biases items can be randomized or alternated.	No randomization of items
Adaptive questioning	Use adaptive questioning (certain items, or only conditionally displayed based on responses to other items) to reduce number and complexity of the questions.	Page 6/ Lines 140-141
Number of Items	What was the number of questionnaire items per page? The number of items is an important factor for the completion rate.	Page 6/ Line 138
Number of screens (pages)	Over how many pages was the questionnaire distributed? The number of items is an important factor for the completion rate.	Page 6/ Line 138
Completeness check	It is technically possible to do consistency or completeness checks before the questionnaire is submitted. Was this done, and if “yes”, how (usually JAVAScript)? An alternative is to check for completeness after the questionnaire has been submitted (and highlight mandatory items). If this has been done, it should be reported. All items should provide a non-response option such as “not applicable” or “rather not say”, and selection of one response option should be enforced.	Page 5-6/ Lines 135-137; Page 6/ Lines 155-157; Page 7/ Lines 179-182. Versions of survey: Supplemental Appendix 1
Review step	State whether respondents were able to review and change their answers (eg, through a Back button or a Review step which displays a summary of the responses and asks the respondents if they are correct).	Page 6/ Lines 143-145
Unique site visitor	If you provide view rates or participation rates, you need to define how you determined a unique visitor. There are different techniques available, based on IP addresses or cookies or both.	NA
View rate (Ratio of unique survey visitors/unique site visitors)	Requires counting unique visitors to the first page of the survey, divided by the number of unique site visitors (not page views!). It is not unusual to have view rates of less than 0.1 % if the survey is voluntary.	NA

For peer review only

Participation rate (Ratio of unique visitors who agreed to participate/unique first survey page visitors)	Count the unique number of people who filled in the first survey page (or agreed to participate, for example by checking a checkbox), divided by visitors who visit the first page of the survey (or the informed consents page, if present). This can also be called “recruitment” rate.	NA
Completion rate (Ratio of users who finished the survey/users who agreed to participate)	The number of people submitting the last questionnaire page, divided by the number of people who agreed to participate (or submitted the first survey page). This is only relevant if there is a separate “informed consent” page or if the survey goes over several pages. This is a measure for attrition. Note that “completion” can involve leaving questionnaire items blank. This is not a measure for how completely questionnaires were filled in. (If you need a measure for this, use the word “completeness rate”.)	NA
Cookies used	Indicate whether cookies were used to assign a unique user identifier to each client computer. If so, mention the page on which the cookie was set and read, and how long the cookie was valid. Were duplicate entries avoided by preventing users access to the survey twice; or were duplicate database entries having the same user ID eliminated before analysis? In the latter case, which entries were kept for analysis (eg, the first entry or the most recent)?	NA<br>Page 15/ Lines 380-386
IP check	Indicate whether the IP address of the client computer was used to identify potential duplicate entries from the same user. If so, mention the period of time for which no two entries from the same IP address were allowed (eg, 24 hours). Were duplicate entries avoided by preventing users with the same IP address access to the survey twice; or were duplicate database entries having the same IP address within a given period of time eliminated before analysis? If the latter, which entries were kept for analysis (eg, the first entry or the most recent)?	NA<br>Page 15/ Lines 380-386
Log file analysis	Indicate whether other techniques to analyze the log file for identification of multiple entries were used. If so, please describe.	NA
Registration	In “closed” (non-open) surveys, users need to login first and it is easier to prevent duplicate entries from the same user. Describe how this was done. For example, was the survey never displayed a second time once the user had filled it in, or was the username stored together with the survey results and later eliminated? If the latter, which entries were kept for analysis (eg, the first entry or the most recent)?	It was an open survey
Handling of incomplete questionnaires	Were only completed questionnaires analyzed? Were questionnaires which terminated early (where, for example, users did not go through all questionnaire pages) also analyzed?	Page 7/ Lines 179-182

For peer review only

Questionnaires submitted with an atypical timestamp	Some investigators may measure the time people needed to fill in a questionnaire and exclude questionnaires that were submitted too soon. Specify the timeframe that was used as a cut-off point, and describe how this point was determined.	NA<br>Page 7/ Lines 175-176
Statistical correction	Indicate whether any methods such as weighting of items or propensity scores have been used to adjust for the non-representative sample; if so, please describe the methods.	NA

This checklist has been modified from Eysenbach G. Improving the quality of Web surveys: the Checklist for Reporting Results of Internet E-Surveys (CHERRIES). J Med Internet Res. 2004 Sep 29;6(3):e34 [erratum in J Med Internet Res. 2012; 14(1): e8.]. Article available at https://www.jmir.org/2004/3/e34/; erratum available https://www.jmir.org/2012/1/e8/. Copyright ©Gunther Eysenbach. Originally published in the Journal of Medical Internet Research, 29.9.2004 and 04.01.2012.

This is an open-access article distributed under the terms of the Creative Commons Attribution License (https://creativecommons.org/licenses/by/2.0/), which permits unrestricted use, distribution, and reproduction in any medium, provided the original work, first published in the Journal of Medical Internet Research, is properly cited.

For peer review only
